# Organic and inorganic nanomaterials: fabrication, properties and applications

**DOI:** 10.1039/d3ra01421e

**Published:** 2023-05-05

**Authors:** Basmah H. Alshammari, Maha M. A. Lashin, Muhammad Adil Mahmood, Fahad S. Al-Mubaddel, Nasir Ilyas, Nasir Rahman, Mohammad Sohail, Aurangzeb Khan, Sherzod Shukhratovich Abdullaev, Rajwali Khan

**Affiliations:** a Department of Chemistry, College of Science, University of Hail Hail 81451 Saudi Arabia; b Department of Electrical Engineering, College of Engineering, Princess Nourah bint Abdulrahman University P.O. Box 84428 Riyadh 11671 Saudi Arabia; c Department of Physics, University of Lakki Marwat Lakki Marwat 28420 KP Pakistan rajwalipak@zju.edu.cn adilaaryan403@gmail.com; d Department of Chemical Engineering, College of Engineering, King Saud University Riyadh 11421 Saudi Arabia; e King Abdullah City for Renewable and Atomic Energy: Energy Research and Innovation Center, (ERIC) Riyadh 11451 Saudi Arabia; f School of Optoelectronic Science and Engineering, University of Electronic Science and Technologyof China Chengdu 611731 P.R. China; g Department of Physics, Abdul Wali Khan University Mardan 23200 KP Pakistan; h Researcher, Faculty of Chemical Engineering, New Uzbekistan University Tashkent Uzbekistan; i Researcher of Scientific Department, Tashkent State Pedagogical University Named After Nizami Tashkent Uzbekistan; j School of Physics and Optoelectronic Engineering, Shenzhen University Nanshan 518000 Shenzhen Guangdong China

## Abstract

Nanomaterials and nanoparticles are a burgeoning field of research and a rapidly expanding technology sector in a wide variety of application domains. Nanomaterials have made exponential progress due to their numerous uses in a variety of fields, particularly the advancement of engineering technology. Nanoparticles are divided into various groups based on the size, shape, and structural morphology of their bodies. The 21st century's defining feature of nanoparticles is their application in the design and production of semiconductor devices made of metals, metal oxides, carbon allotropes, and chalcogenides. For the researchers, these materials then opened a new door to a variety of applications, including energy storage, catalysis, and biosensors, as well as devices for conversion and medicinal uses. For chemical and thermal applications, ZnO is one of the most stable n-type semiconducting materials available. It is utilised in a wide range of products, from luminous materials to batteries, supercapacitors, solar cells to biomedical photocatalysis sensors, and it may be found in a number of forms, including pellets, nanoparticles, bulk crystals, and thin films. The distinctive physiochemical characteristics of semiconducting metal oxides are particularly responsible for this. ZnO nanostructures differ depending on the synthesis conditions, growth method, growth process, and substrate type. A number of distinct growth strategies for ZnO nanostructures, including chemical, physical, and biological methods, have been recorded. These nanostructures may be synthesized very simply at very low temperatures. This review focuses on and summarizes recent achievements in fabricating semiconductor devices based on nanostructured materials as 2D materials as well as rapidly developing hybrid structures. Apart from this, challenges and promising prospects in this research field are also discussed.

## Abstract 2

ZnO nanomaterials and nanoparticles are a burgeoning field of research and a rapidly expanding technological sector in a wide variety of application domains.

## Graphical abstract

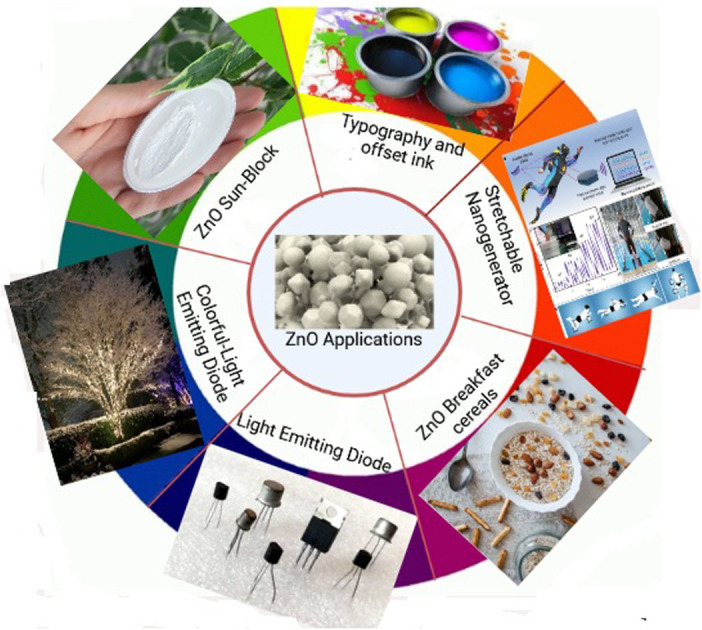

## 1. Nanomaterials

Nanomaterials (NMs) and nanoparticles (NPs) are a fast increasing technical industry and a blossoming topic of study in a wide range of application disciplines. Due to their adjustable physicochemical properties such as wettability, scattering, light absorption, thermal and electrical conductivity, catalytic activity and melting point.^1^ NPs and NMs have acquired significance in technological breakthroughs. A nanometer is a SI (System international of units, SI) unit of length equal to 10^−9^ metres. NMs are characterised in principle as materials with at least one dimension length of 1–1000 nm; nonetheless, they are frequently defined as having a diameter of 1–100 nm. Today, various articles of legislation in the United States of America (USA) and the European Union (EU) make specific mention to NMs. However, there is no universally accepted definition of NMs. Different organisations have divergent views on how to define NMs.^2^ “NMs can display behaviour unrelated to chemical components of comparable size”, according to the Environmental Protection Agency.^3^ Nanomaterials are also known as “materials with at least one dimension in the 1–100 nm range that exhibit dimension dependent behaviours”, according to the US Food and Drug Administration.^4^ The International Organization for Standardization defines Nanomaterials as “any exterior nanoscale dimension or surface structure with an internal nanoscale dimension”,^5^ This ISO definition has been used to define quantum dots, nanowires, nanoplates, nanofibers and other related words.^6^ According to the EU Commission, a nanomaterial is “a man-made or naturally occurring substance that can be unbound, aggregated, or agglomerated and has particles whose sizes on the surface range from 1 to 100 nanometers”.^7^

Since the last century, nanotechnology has been a well-known field of study. Richard P. Feynman is credited with coining the phrase “nanotechnology” in the year 1959, when he delivered the famous lecture “There is Plenty of Room at the Bottom”.^9^ Nanoscale technology has been used to create a variety of materials. Nanoparticles are divided into several categories. Nanoparticles are particles with a diameter of 1 to 100 nanometers.^10^ Depending on the shape, nanoparticles can be 0D, 1D, 2D, or 3D.^11^

## 2. Classification of nanomaterials

Nanoparticles are categorized into their respective categories based on their morphology, which refers to their structure, as well as their size and shape. The nanoparticles listed below are some of the most significant types currently known.

### 2.1 Organic nanomaterials

**Fig. 1:**
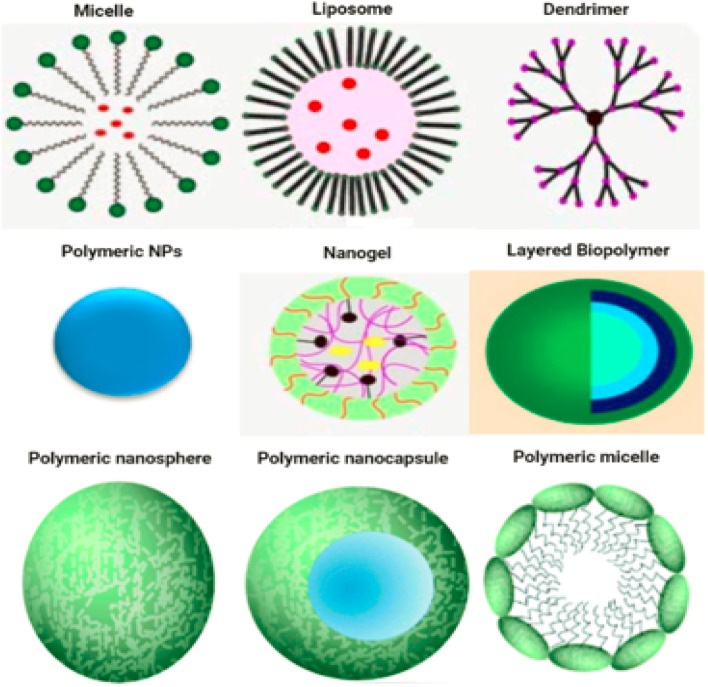
The Schematic diagram of organic nanoparticles.

Fig. 1 displays a number of different types of organic nanoparticles, some of which are micelles, dendrimers, liposomes, nanogels, polymeric NPs, and layered biopolymer. Certain organic nanoparticles, such as micelles and liposomes, have a hollow sphere, and they are non-toxic and biodegradable. Organic nanoparticles can also be broken down naturally. This name is also used to refer to nanocapsules, which are extremely sensitive to light and heat.^12^ Due to the fact that organic nanoparticles exhibit these properties, they are an excellent option for the transportation of pharmaceuticals. Nanoparticles are also frequently used in the process of transporting target medications to their intended locations. Organic nanoparticles are also sometimes referred to by the label polymeric nanoparticles. The nanosphere or nanocapsule is the most famous form of polymeric or organic nanoparticles.^13^ The matrix particles have a solid sphere of mass and adsorb other molecules at the outer boundary of spherical surface. Particles encapsulated the solid mass in the later case.^14^Fig. 1 displaying the organic nanoparticles.

### 2.2. Inorganic nanomaterials

Inorganic nanoparticles do not contain carbon. Inorganic nanoparticles have the advantages of being hydrophilic, non-toxic, and biocompatible with living systems. The stability of inorganic nanoparticles is superior to that of organic nanoparticles.

**Fig. 2:**
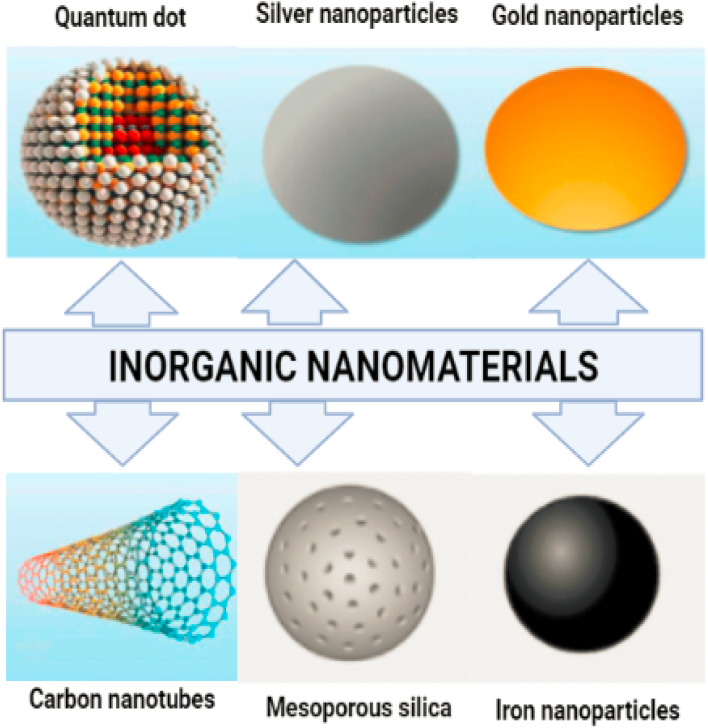
Inorganic nanoparticles, metal and metal oxide nanoparticles are categorized as inorganic nanoparticles.

Magnetic nanoparticles (mNPs) are one of the most significant inorganic nanomaterials.^15^ A magnetic core (*e.g.* maghemite (g-Fe_2_O_3_) or magnetite (Fe_3_O_4_)) is generally present.^16^ Other metals, such as nickel and cobalt, are also employed, although their applications are limited due to their toxicity and oxidation vulnerability.^17^ Ferritin, a type of protein, is where the vast majority of iron is kept in the human body. Iron oxide mNPs have the ability to digest excess iron and restore the supply in the human body. There is a continuous presence of these cationic mNPs in the endosomes for a considerable amount of time. This continues to be the case over and over again.^18^ After that, during the postcellular absorption process that takes place in the endosome and the lysosome, elemental components like iron and oxygen are brought into the body's storage, where hydrolytic enzymes either digest them or cause their destruction. In the human body, homeostasis is the process through which iron levels are maintained and adjusted. Adsorption, excretion, and storage are all processes that contribute to this process. Iron oxide nanoparticles help the body digest any excess iron that may be present.^19^ Iron is essential in almost all biological tissues, yet it has a low bioavailability. In certain circumstances, it can damage the cells when they are in the form of free iron or when it is not associated with haemoglobin. Additionally, it can be harmful to cells when it is present alone. Fig. 2 displaying the inorganic nanomaterials.

#### 2.2.1. Metal nanomaterials

**Fig. 3:**
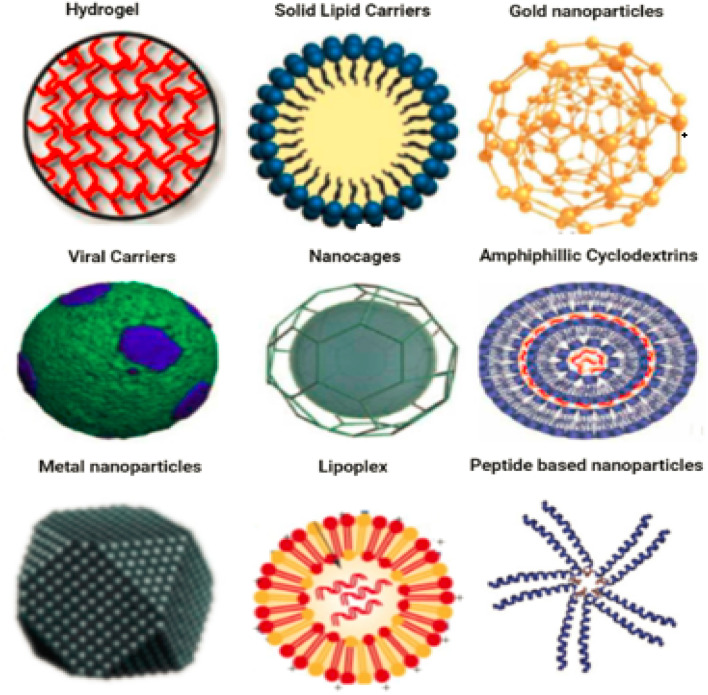
Metal nanomaterials.

Nanoparticles of metallica can be synthesized from metals through either constructive or destructive mechanisms. In order to create pure metal nanoparticles, metal precursors are required for the production process. Metal nanoparticles have distinctive optoelectrical properties, which can be attributed to their plasma on resonance characteristics.^20^ The size, shape, and surface of the metal nanoparticles all play a role in the synthesis process in their own unique ways.^21^ All metal nanoparticles can be synthesised.^22^ Nanoparticles of the metals cadmium, aluminium, copper, silver, lead, cobalt, zinc, gold, and iron are all examples of well-known nanoparticles of the element. Nanoparticles can be identified by their smaller size (10 to 100 nm), their surface properties such as pore size, surface charge, environmental factors, surface area to volume ratio, structure (amorphous and crystalline), shapes (irregular, rod, spherical, cylindrical, tetragonal and hexagonal), surface charge density and colour. Nanoparticles have been shown to have a variety of applications in a variety of fields (air, heat, moisture and sunlight). In the field of medicine, nanoparticles made of zinc, gold, silver, platinum, iron and copper, as well as those made of other metals, have garnered a lot of attention. Metallic nanoparticles can occur in solution, as Sathyanarayanan (2013)^23^ demonstrated. Later onwards, Salas *et al.* (2019)^24^ conducted research on the colour and shape of metallic nanoparticles. Changing the chemical groups that help antibodies bind to nanoparticles may now be done during the manufacturing process, which also allows for improvements. Noble metal nanoparticles have been utilized in a diversity of biomedical applications, including those that treat cancer, diagnose diseases, improve the effectiveness of radiotherapy, fight germs and fungi, perform thermal ablation, deliver drugs and transport genes (Au, Pt, Ag). Nanoparticles made of noble metals have a number of unique properties, all of which contribute to their increased value. In order to target a wide variety of cell types, metal nanoparticles can be functionalized with a wide variety of functional groups, including antibodies, peptides, DNA, and RNA, as well as biocompatible polymers, such as polyethylene glycol. (Prasanna *et al.*, 2019).^25^Fig. 3 displaying the metal nanomaterials.

#### 2.2.2. Metal-oxide nanomaterials

**Fig. 4:**
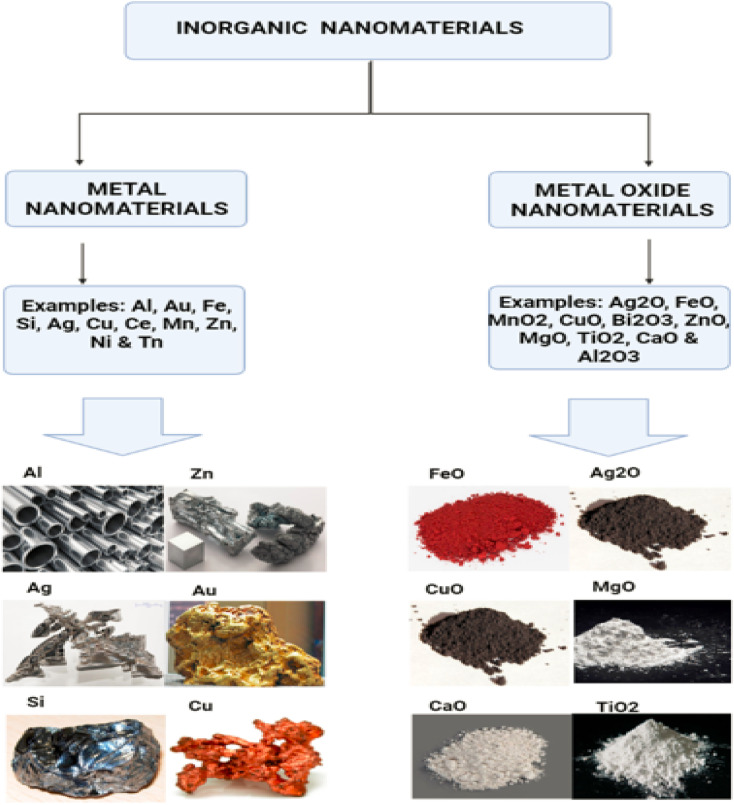
Different types of metal and metal oxide-based nanomaterials.

The major goal in the manufacture of metal oxide nanomaterials is the alteration of the properties of the relevant metal nanomaterials. One example of this would be the transformation of iron nanoparticles into iron oxide nanoparticles. When compared to the reactivity of nanoparticles made of iron, the reactivity of nanoparticles made of iron oxide is substantially higher. Nanoparticles of metal oxide are produced when the efficiency and reactivity of metal oxide are increased. This results in the production of the nanoparticles.^26^ Zinc oxide, iron oxide, silicon dioxide, magnetite, titanium oxide, aluminium oxide and cerium oxide are examples of metal oxide nanomaterials. Nanoparticles made of metal oxides have shown promising outcomes in studies conducted in the field of biomedicine. Antibacterial activity has been demonstrated for a wide variety of metal oxide nanoparticles, including but not limited to MnO_2_, FeO, Ag_2_O, ZnO, Bi_2_O_3_, CuO, CaO, Al_2_O_3_, MgO, and TiO_2_. Researchers Sigmund *et al.* (2006)^27^ looked into the impacts of Ag_2_O nanoparticles and came to the conclusion that they could be a potential new source of antibiotics. Thomas *et al.* (2015)^28^ also revealed that Ag_2_O nanoparticles had antibacterial capabilities against *E. coli*. Zinc oxide nanoparticles demonstrated strong bactericidal efficacy both Gram-negative and Gram-positive bacteria and spores. High pressure and temperature have little effect on these bacteria and spores. In addition, Fadeel *et al.* (2010)^29^ looked at the antibacterial characteristics of ZnO at a range of different particle sizes. According to the findings, the bactericidal effectiveness of ZnO nanoparticles increased in direct proportion to the decrease in particle size. Akif *et al.* (2022)^30^ investigated the antibacterial effects of ZnO, Fe_2_O_3_ and CuO nanoparticles on Gram-negative bacteria such as *P. aeruginosa*, *E. coli*, and others, as well as Gram-positive bacteria such as Bacillus subtilis and *Staphylococcus aureus*. They found that all three nanoparticles inhibited the growth of the bacteria. According to these data, ZnO has a potent antimicrobial impact, whereas Fe_2_O_3_ nanoparticles have the weakest effect against bacteria. Fig. 4 displaying the metal & metal oxide nanomaterials.

### 2.3. Ceramics nanomaterials

Ceramic nanoparticles are also referred to as nonmetallic solids in some circles. The process of synthesising ceramic nanoparticles involves periodically heating or cooling the material. Ceramic nanoparticles can have a variety of different structures, including amorphous, polycrystalline, dense, hollow or porous.^32^ These nanoparticles are of interest to the researchers due to the vast range of applications that can be achieved with their utilisation, including photocatalysis, dye photodegradation, imaging and catalysis.^33^

The research and development of innovative ceramic materials with potential uses in biomedicine is currently advancing at a rapid speed. In order to reduce the cytotoxicity of nanoscale ceramics such titanium oxide (TiO_2_), hydroxyapatite (HA), alumina (Al_2_O_3_) silica (SiO_2_), and zirconia (ZrO_2_) in biological systems, new synthetic techniques were utilized to optimize the physical-chemical characteristics of these nanoscale ceramics. Nonetheless, when novel ceramic materials were used, the host had negative reactions (in a number of organs, including the immune system). When it comes to the applications of ceramic nanoparticles in biomedicine, regulated drug release is one of the sectors that has received the most attention. In this field, the size and the dose are extremely significant factors. Nanoparticles are a promising technique for managing drug delivery due to a number of properties, including their load capacity and high stability, as well as their ease of absorption into both hydrophilic and hydrophobic systems. Additionally, nanoparticles can be administered *via* a variety of different routes (inhalation, oral, *etc.*). A wide variety of organic groups that are capable of being functionalized on its surfaces also make it possible to achieve a specific effect. Titanium dioxide is a photocatalytic substance with a wide range of dielectric and optical properties due to its various crystalline structures.

Titanium dioxide nanoparticles are stable in anatase at the nanoscale; nonetheless, they also have the maximum level of cytotoxicity in the region of 3 to 10 nm, which is more than 100 times the scale in the rutile phase. Nanoparticles like this are regularly put to use as drug-eluting carriers or excipient formulations in the field of pharmacology. In point of fact, they are being used in photodynamic therapy due to the fact that they photooxidize oxygen quite well. In addition, the cytotoxic properties of nanoparticles are lessened when they are combined with other substances for example, hydroxyapatite. The pharmaceutically active mesoporous silica molecules have a number of key properties, some of the most important of which are the automatic release of prospective drugs, the ease with which they can be dissolved, and their availability in the organism.

**Fig. 5:**
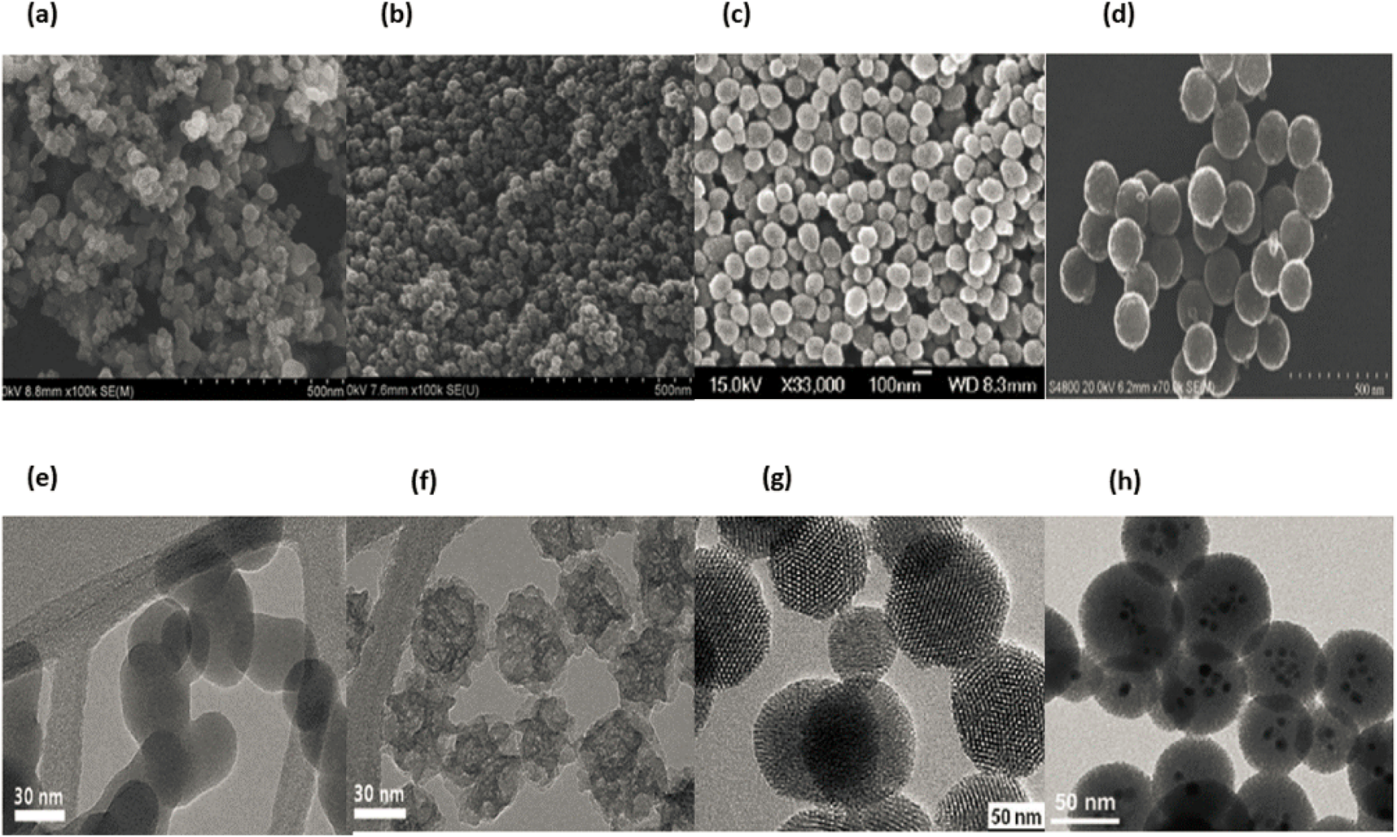
SEM and TEM micrograph of SiO_2_ nanoparticles. (a) and (b) reproduced with permission. Copyright 2011, Nature,^30^ (c) reproduced with permission. Copyright 2012, Royal Society Chemistry.^31^ (d) Reproduced with permission. Copyright 2012, Royal Society of Open Science,^32^ (e) and (f) reproduced with permission. Copyright 201, Nature,^30^ (g) and (h) reproduced with permission. Copyright 2012, Royal Society Chemistry.^31^

Because even a minor shift in the conditions of the synthesis can result in variable forms, sizes, and subsequent physicochemical properties, it is challenging to develop strategies that can combine biocompatibility and minimise the harmful effects that these nanoparticles may exhibit in biological systems. This is because it is difficult to create methods that can combine biocompatible materials with nanoparticles. Because of this, it is difficult to develop techniques that can combine biocompatibility and minimise the bad effects that these nanoparticles may exhibit in biological systems. This is due to the fact that it is difficult to create methods that can combine biocompatibility with nanoparticles. This makes it difficult to find methods that can combine biocompatibility and physicochemical properties.^34^Fig. 5a–d displaying SEM & Fig. 5e–h displaying TEM images of SiO_2_ nanoparticles.

### 2.4. Bionanomaterials

**Fig. 6:**
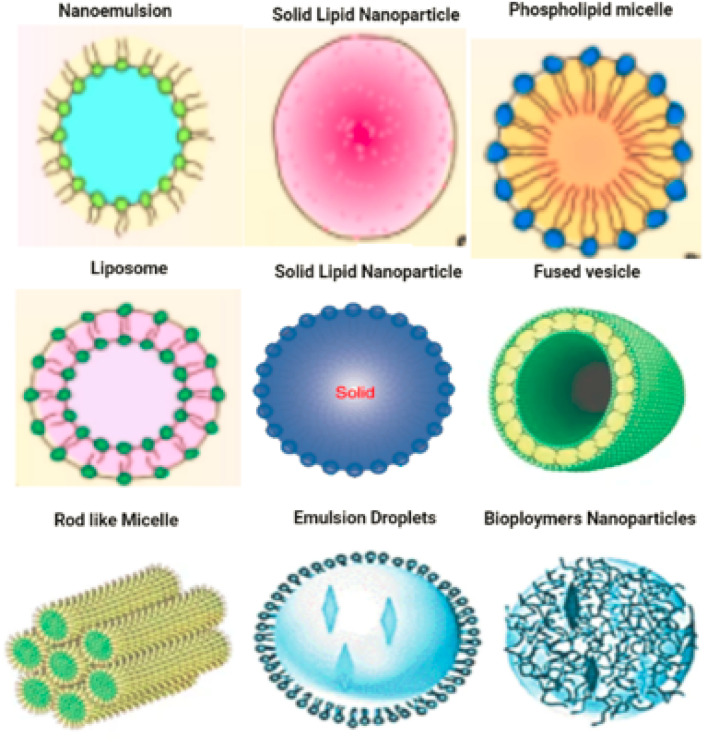
Bionanoparticles include exosomes, magnetosomes, lipoproteins, viruses and ferritin.

A biological or bio-nanomaterials is an assembly of atoms or molecules that is produced in a biological system and has at least one dimension that falls within the range of 1 to 100 nm.^35^ Other terms for this type of particle include bio-nanomaterial and biological nanomaterial. Nanoparticles that can be found in nature are referred to as bionanoparticles. These nanoparticles can either have an extracellular or an intracellular structure, depending on their location. Magnetosomes are an example of an internal structure, whereas viruses and lipoproteins are examples of structures that are found outside of cells. Bionanoparticles include exosomes, magnetosomes, lipoproteins, viruses and ferritin.^36^Fig. 6 displaying the bionanomaterials.

### 2.5. Carbon based nanomaterials

Carbon-based materials have been debated as valuable treasure in recent times owing to the presence of a variety of allotropes of carbon, which range from well-known allotropic phases like amorphous diamond, graphite and carbon to recently discovered allotropes like opportune graphene quantum dots (GQDs), fullerene, graphene oxide (GO) and carbon nanotubes (CNTs). Amorphous carbon is one of the most common forms of carbon.^37^ Single-walled carbon nanotubes (SWCNTs) and multi-walled carbon nanotubes (MWCNTs) are the two categories that can be used to categorise carbon nanotubes. A carbon nanotube is a hollow cylinder that is constructed of graphitic sheets. After rolling out a single graphitic sheet with a high aspect ratio, single-walled carbon nanotubes with a cylindrical nanostructure were produced. Multi-walled carbon nanotube is composed of a few graphitic layers arranged in a rolling pattern with a gap of 3.4 angstroms between each layer.^38^ Graphene possesses a wide array of exceptional qualities, any one of which could make it an asset for use in bio-applications. Simple functionalization has the ability to result in a rise in the number of functional groups on the surface of the material, which then permits the precise and selective detection of a variety of biological components. In addition, it is an excellent option for the delivery of pharmaceuticals due to the exceptionally wide surface area it contains, the chemical purity it possesses, and the free electrons it possesses. This makes it a great alternative for the administration of pharmaceuticals.^39,40^ Another appealing biomaterial from the carbon family that has recently been developed is graphene quantum dots, it has lateral dimensions of less than 100 nm and is comprised of a single layer or a few layers and is described as a zero-dimensional graphene sheet.^41^ As a result of the quantum confinement that takes place when two-dimensional graphene sheets are converted into graphene quantum dots, the photoluminescence properties of the graphene quantum dots are enhanced to an exceptional standard.^42^ Surprisingly, graphene quantum dots exhibits excellent biocompatibility and photo-bleaching durability relative to conventional fluorochromes or semiconductor quantum dots. This is because graphene quantum dots are made from natural materials. In addition, graphene quantum dots have important graphene properties, such as accessible electrons and a large surface area. These properties make graphene quantum dots a smart nanomaterial for a variety of biomedical applications, including biomolecule sensing, cancer therapy, imaging, targeted drug delivery, and so on. Graphene quantum dots have been shown to be useful in these applications. In addition, graphene quantum dots have a large surface area and accessible electrons.^43,44^

**Fig. 7:**
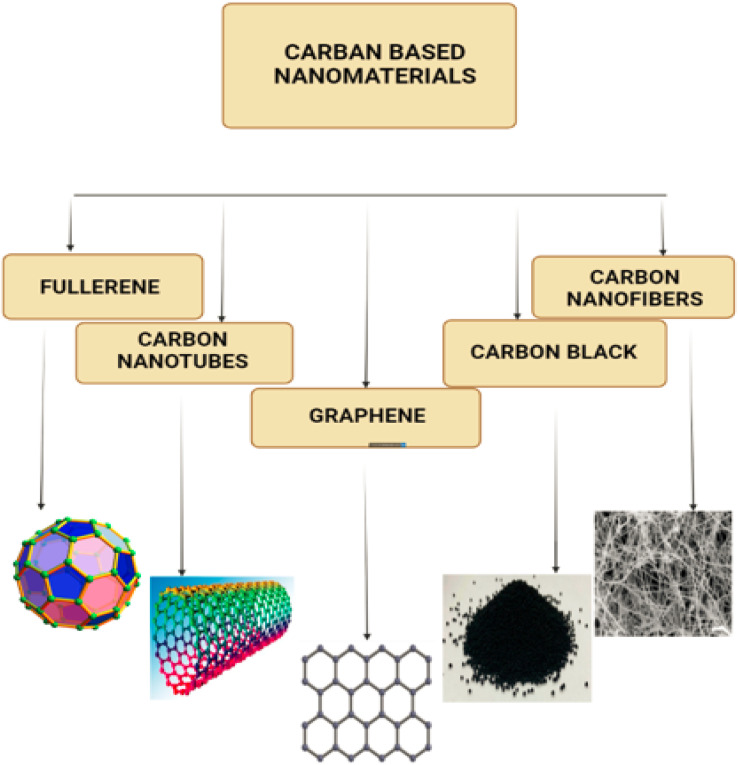
Carbon based nanomaterials. Carbon-based nanoparticles include carbon nanotubes, graphene, carbon nanofibers, fullerenes and carbon black.

Carbon-based nanomaterials are formed entirely of carbon. Carbon-based nanoparticles include carbon nanotubes, graphene, carbon nanofibers, fullerenes and carbon black.^45^Fig. 7 displaying the carbon based nanomaterials.

#### 2.5.1. Fullerene

**Fig. 8:**
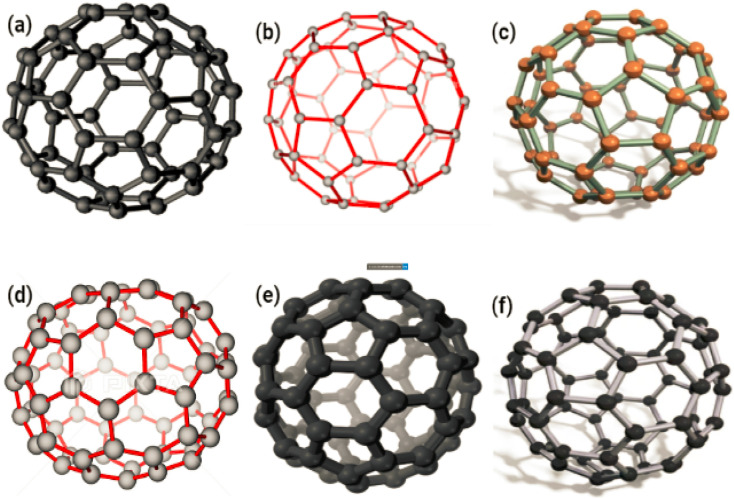
(a–c) Shows the Fullerenes in different forms, C_60_ and (d–f) shows the C_70_ carbon nanotubes.

Fullerene has a highly symmetric cage that can range in size from C_60_, C_70_, and beyond due of its unusual structure of sp^2^ carbons. In the as-synthesized formulation, C_60_ is the most prevalent fullerene, and its molecular structure, which can be shown in Fig. 8 together with that of C_70_, can be seen to be very similar.^46^ C_60_ is composed of 60 carbon atoms bonded to one another by single C_5_–C_5_ bonds, which result in the formation of 12 pentagons, and double C_5_–C_6_ bonds, which result in the formation of 20 hexagons.^47^ In reality, a ‘*n*’ hexagon is present in every fullerene that has 2*n* + 20 carbon atoms.^48^ Fullerene C_60_ has the shape of a football, and according to Yadaf and colleagues, the diameter of the earth is 12.75 × 10^6^ metres, the diameter of a soccer ball is 2.2 × 10^1^ metres, and the diameter of a fullerene molecule is 7.0 × 10^10^ metres. According to the findings of the study, the proportion of a fullerene molecule to a soccer ball is analogous to the proportion of a soccer ball to the earth.^49^

In terms of crystallographic properties, the presence of symmetric elements such as 20 tripled axes, 12 fivefold axes and 30 twofold axes has made fullerene the most symmetrical molecule that is regulated by the Golden Mean rule (as was previously stated). Fullerene also possesses 12 fivefold axes and 20 tripled axes.^49,50^ C_60_ has a structure that is sufficiently stable that it may be described as having face-centered cubic lattices in its solid phase where fullerene cage disintegration occurs at temperatures more than 10008 °C. To analyse fullerene, many spectroscopic approaches such as FTIR, NMR, UV-vis, and Raman spectroscopy could be used.^46^ Furthermore, Biomolecules, particularly those structured by the Fibonacci sequence and exhibiting Golden Mean properties, have discovered fullerene to be a promising nanomaterial.^51^ C_60_ is an outstanding candidate for photodynamic therapy due to the fact that, among its many properties, it possesses the capacity to generate oxygen species when it is illuminated by visible light.^52^ The puzzling behaviour of fullerene in solutions is evidence of a newly known interaction between solvents and solute. Because of this interaction, the fullerene molecule has not changed conformationally or in a way that is dependent on the solvent, nor has it taken on the shape of a hexagon. Fullerenes have sp^2^ hybridized carbon atoms that connect them together. Fullerenes constructed of C_70_ or C_60_ have diameters of 7.648 and 7.114 nm,^53^ respectively. A single layer of fullerene or a multilayer of fullerene can be used. Fig. 8a–c displaying the fullerene C_60_ & Fig. 8d–f displaying the fullerene C_70_.

Carbon may bond in a variety of ways to develop structures with vastly diverse properties. Carbon sp^2^ hybridization produces a layered structure with strong inplane limitations and modest out-of-plane van der Waals bonding. Multi walled carbon nanotubes are produced by surrounding a standard core hollow with a few to a few tens of concentric cylinders with regular periodic interlayer spacing. An interlayer spacing range was discovered during realspace evaluation of multiwall nanotube images (0.34–0.39 nm).^56^ MWCNTs can have an interal diameter anywhere from 0.4 nm to a few nanometers, while their outside diameters can range anywhere from 2 nm to 20 to 30 nm, dependent on the number of layers they are composed of. Both of the normally closed tips of the MWCNT have dome-shaped half-fullerene molecules capping them. These defects, which are also referred to as pentagonal defects, cap the normally closed points. The axial sizes of these defects range from one metre to just a few centimetres in width. The purpose of the half-fullerene molecules, which are also referred to as a pentagonal ring defect, is to make the operation of capping off both ends of the tube more easy. On the other side, single-wall carbon nanotubes (SWCNTs) have diameters that can be anywhere from 0.4 to 2 to 3 nanometers, while their lengths typically fall somewhere in the micrometre zone. In most cases, SWCNTs are able to create bundles by joining together (ropes). Within a bundle structure, the SWCNTs are organised in a hexagonal pattern to create a structure similar to a crystal.^57^

**Fig. 9:**
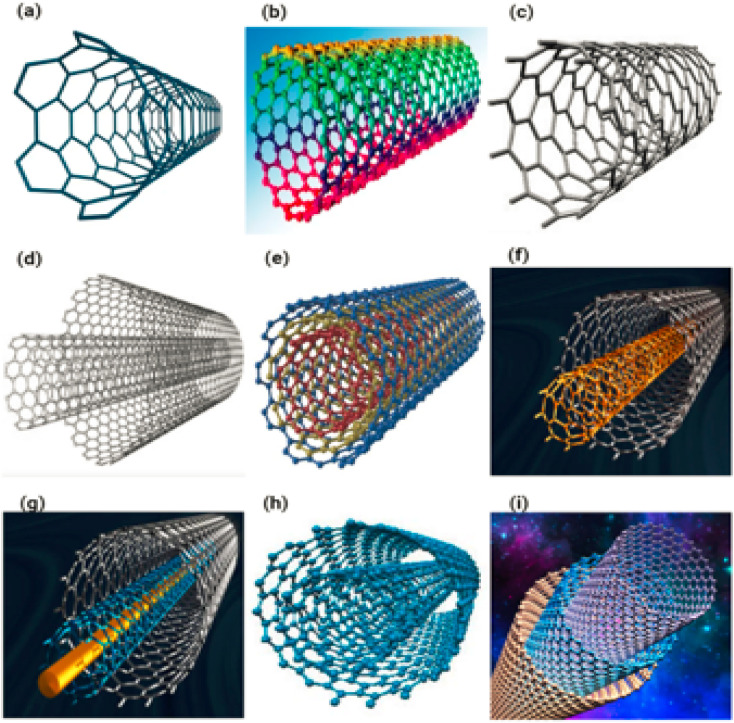
Single wall nanotubes are shown in (a–c), double wall nanotubes are shown in (d–f) and multiple wall nanotubes are shown in (g–i) which are made from graphene sheet.

Carbon nano tubes are elongated tubular structures with a diameter of 1 to 2 nm.^58^ Based on diameter, a carbon nanotube can be classified as semiconducting or metallic.^59^ CNT has a structure that looks like a graphite sheet rolling on itself. Fig. 9 shows how (a–c), (d–f) and (g–i) single-walled nanotubes (SWNTs), double-walled nanotubes (DWNTs) and multi-walled nanotubes (MWNTs) looks like. Which is further classified based on their rolling properties.

##### 2.5.1.1. Single walled carbon nanotubes

Carbon has a ground state structure of 2s^2^2p^2^ with a valence shell of four electrons. Both graphite and diamond are naturally occurring crystalline forms of pure carbon. Graphite is the more common of the two. Unlike graphite, which has sp^2^ hybridization, diamond possesses sp^3^ hybridization, which gives it its amazing rigidity. Graphite, on the other hand, has sp^2^ hybridization. Along the *x*–*y* plane, each carbon atom in graphite forms C–C bonds with three more carbon atoms at an angle of 120°, while a – bond is formed along the *z* axis.^54^ In sp^2^ hybridization, the C–C bond length is 1.42 nm and the spacing between carbon layers is 3.35 nm.^55,56^ Graphite is an exceptional electrical conductor because it possesses delocalized electrons that are free to flow throughout the structure of the graphite. This makes graphite a wonderful material. Carbon nanotubes, also known as CNTs, are hexagonal networks made up of carbon atoms. The diameter of a carbon nanotube can range from one to one hundred metres, and it has a diameter of one nanometer. CNTs are cylindrical structures that are made up of sheets of graphene that have been rolled up to form a continuous tube.^55^ In the middle of the 1970s, Endo was able to take the very first high resolution transmission electron microcopy (HRTEM) pictures of carbon nanotubes.^57^ Later, Iijima^58^ found helical carbon microtubules, now known as nanotubes, using HRTEM and electron diffraction in the Arc-Discharge Fullerene Reactor.^58^ Single-walled carbon nanotubes, also known as SWNTs, are cylinders with a nanometer-scale diameter that are composed of a single sheet of graphene that has been wrapped around to form a tube. Nanowires can be metallic or semiconducting based on the chirality of SWNTs.^59–62^ The level of twist in the graphene sheet is the primary factor that influences the electrical conductivity of carbon nanotubes.

A single rolled sheet is used to make single-walled nanotubes. Single-walled nanotubes have a diameter of 0.7 nanometers. The length varies depending on the method used to prepare it.^63^Fig. 9a–c displaying the single walled Carbon nanotubes.

##### 2.5.1.2. Double walled carbon nanotubes

Carbon nanotubes, also known as pure carbon polymer chains, are nanometer-sized cylindrical structures made up of single or concentric multilayers of graphene sheets. Many scientists have been interested in double walled carbon nanotubes in recent years because their intrinsic coaxial topologies give birth to novel electrical and mechanical properties that have not been previously observed. We were able to determine whether or not double-walled carbon nanotubes of a particularly high purity behave as quantum wires and whether or not there is a symmetric relationship between concentric tubes during the growing process by painstakingly preparing them. Also investigated was the shell influence on the electrical conductivity as well as the adsorption characteristics of a coaxial nanotube wire. When compared to single walled carbon nanotubes (SWCNTs), double walled carbon nanotubes (DWCNTs) and multi walled carbon nanotubes (MWCNTs) are preferred materials for bi-cables, atomic force microscopy tips, hydrogen storage materials, electrochemical electrodes, nanocomposites, field emission display sources, nanotube and various electrical devices.^64^

Double-walled nanotubes are made up of two layers of rolled sheet. Fig. 9d–f displaying the double walled carbon nanotubes.

##### 2.5.1.3. Multi walled carbon nanotubes

Multiple rolled sheets make up Multiwalled Nanotubes (MWNTs). Multi-walled nanotubes have a minimum diameter of 100 nm. A graphene nanofoil with a hexagonal carbon lattice is coiled into a cylindrical shape to make nanotubes. Carbon tubes range in length from a few micrometres to many millimetres. CNT is a strong material.^65^ When CNT is bent, it returns to its original shape without becoming brittle. CNT has a variety of structures and shapes, as well as varied thicknesses, lengths, and layers,^66^ but its properties are based on graphene sheets. Fig. 9g–i displaying the multi walled carbon nanotubes.

#### 2.5.2. Graphene

**Fig. 10:**
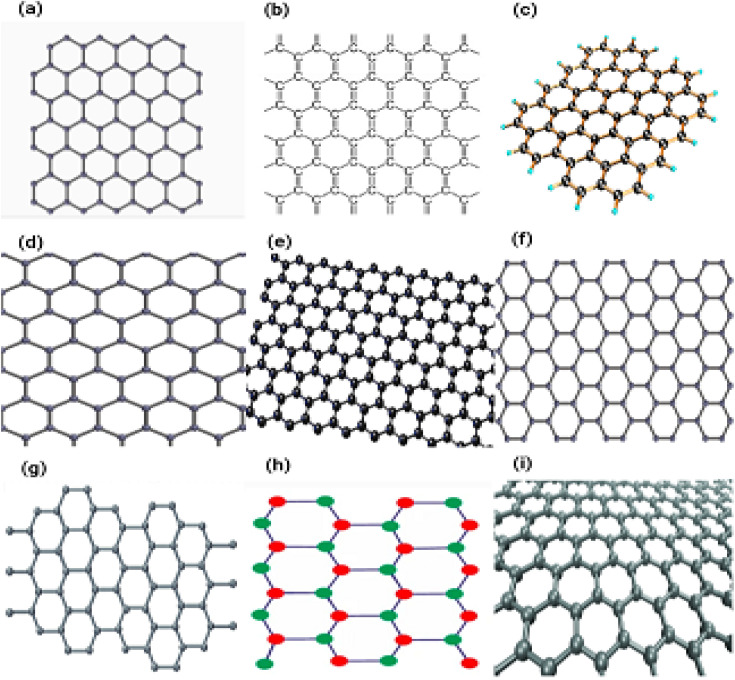
(a–i) Displaying the different types of graphenes.

The first two-dimensional atomic crystal to be synthesised in a laboratory is graphene. They are employed in a wide number of applications due to the remarkable chemical and physical qualities, such as elasticity, mechanical stiffness and strength, as well as extraordinarily high thermal and electrical conductivity, that they possess.^67,68^ Graphene is a carbon allotrope. It's a planar hexagonal honeycomb carbon atom lattice with a two-dimensional hexagonal honeycomb carbon atom lattice. The graphene layer is 1 nm thick. Graphene is made up of a single layer of carbon atoms that are sp^2^-bonded to one another in a honeycomb lattice. Since Novoselov *et al.* initial's synthesis at Manchester University in 2004,^69^ Because of its excellent physical properties, graphene has attracted considerable attention and scientific curiosity.^69^ Graphene is a semiconducting material that has no effective mass and a band gap that is equal to zero.^67,70,71^ At ambient conditions, it exhibits a significant ambipolar electric field effect with a high charge carrier mobility (up to 10 000 cm^2^ V^−1^ s^−1^).^67,69^ Graphene is the strongest material that has ever been examined, as evidenced by its breaking strength of 42 Nm^−1^ and its Young's modulus of approximately equal 1.0 TPa.^72^ Due to these fascinating features, graphene has demonstrated promise in a variety of applications, including electrical and photonic devices,^73,74^ sensing platforms,^75,76^ and clean energy applications.^77^Fig. 10a–i displaying the different types of graphenes.

#### 2.5.3. Carbon nanofibres

Carbon is a chemically unique element. Because of its unique electrical structure, it is able to make covalent bonds, either in the form of rings or long chains, with other chemical elements such as hydrogen, as well as with itself. Carbon nanofibers, also known as CNFs, are a type of nanomaterial that only exists in one dimension and have a more intricate structure than carbon nanotubes (CNTs). Because of their one-of-a-kind properties, CNFs are well-suited for a diverse range of applications, such as hydrogen storage, electrochemical catalysis, polymer reinforcing and selective adsorption.^78–80^ The direction in which carbon layers in CNFs are arranged has an effect on their mechanical properties. Carbon nanofibres are structured as discontinuous linear filaments based on sp^2^, having an average aspect ratio that is larger than 100 : 1.^81^ The identical graphene nanofoils that are used to make carbon nanotubes are transferred into carbon nanofiber; however, rather than being twisted into long cylindrical tubes, the nanofoils are formed into a cup or cone.

**Fig. 11:**
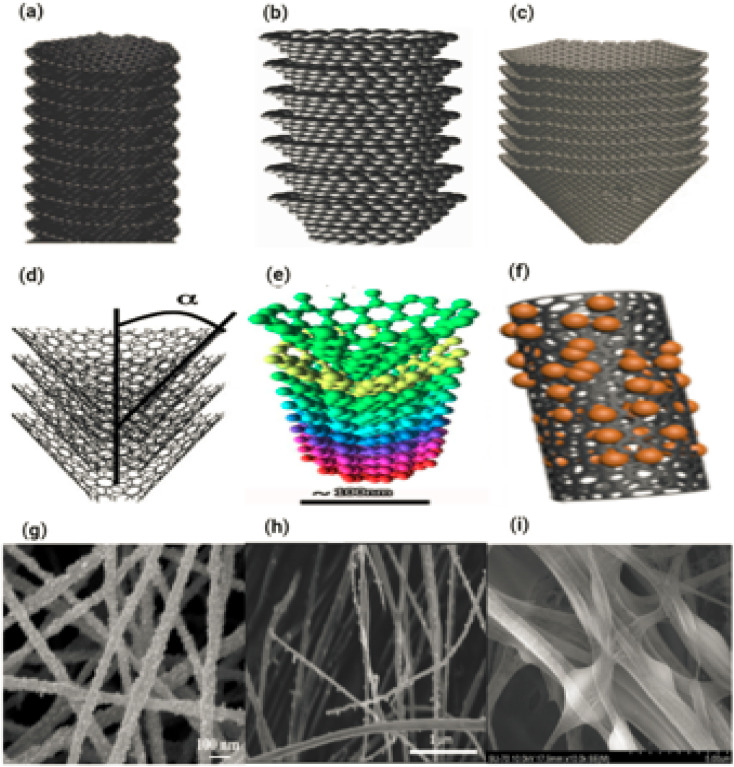
(a–i) Displaying the different type of carbon nanofibers.

In the majority of carbon nanofibers, subsequent examinations revealed that the layers of graphitic planes are not adjusted along the axis of the fibre, as the name suggests.^82^ Carbon nanofibers can take on a variety of morphologies, as illustrated in Fig. 11, controlled by the angle of the graphene layers that comprise the filament.^82^ In addition to platelet carbon nanofibers (shown in Fig. 11a–e) and ribbon or tubular carbon nanofibers (also known as carbon nanotubes) (shown in Fig. 11f–i), there is another type of carbon nanofiber known as fishbone carbon nanofibers. In fishbone carbon nanofibers, the graphene layers are arranged at an angle to the primary and perpendicular axes of the nanofiber. Fig. 11a–i displaying the different type of carbon nanofibers.

#### 2.5.4. Carbon black

**Fig. 12:**
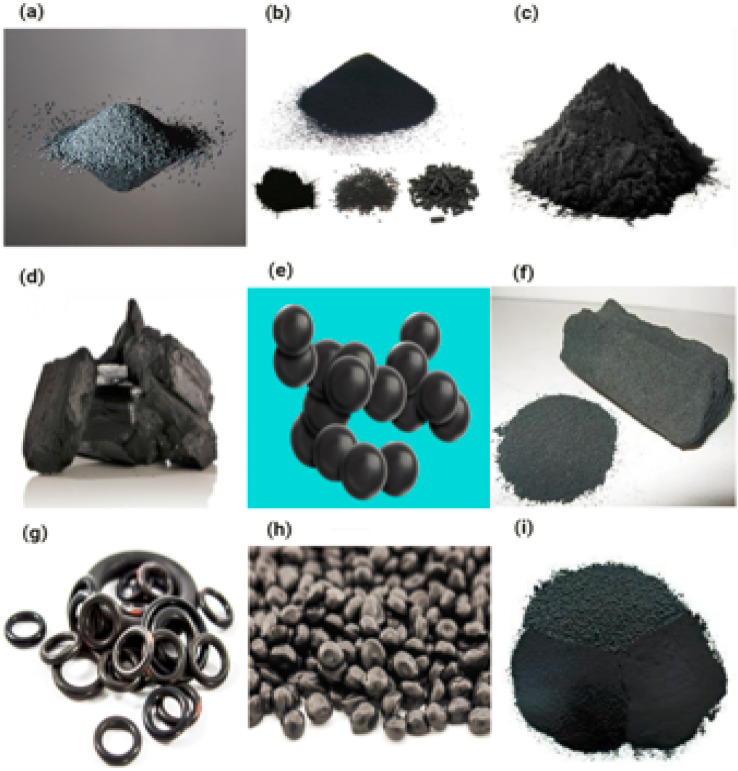
(a–i) Displaying the carbon black.

Carbon black is the brand name for a variety of produced fine-particle materials that are also sold under a wide range of other business names and have a range of different physicochemical features, but almost entirely consist of EC. These products are referred to as “carbon black” because they are sold under this brand name. CB has been produced on a commercial scale for more than a century, and with a global production that totaled around 9.8 million metric tonnes in 2008, it has been recognised as one of the top fifty industrial chemicals produced anywhere in the world.^83,84^ Rubber applications account for approximately 90% of CB use in the Japan, United States and Western Europe. These applications include rubber automotive products (*e.g.*, hoses, belts, and miscellaneous), tire-related automobiles applications and non-automotive industrial rubber products.^83,84^ The remaining 10% is allocated to various specialty CB applications, such as UV absorber, pigment and conductor in polymers, inks, and coatings.^85,86^ It is made up of carbon and is an amorphous substance. Carbon black has a spherical form. The diameter varies between 20 and 70 nanometers. Fig. 12a–i displaying the carbon black.

## 3. Classification of nanomaterials on the basis of dimensions

One-dimensional nanomaterials, two-dimensional nanomaterials, and three-dimensional nanomaterials are the three types of nanomaterials.

### 3.1. One dimension nanomaterials

**Fig. 13:**
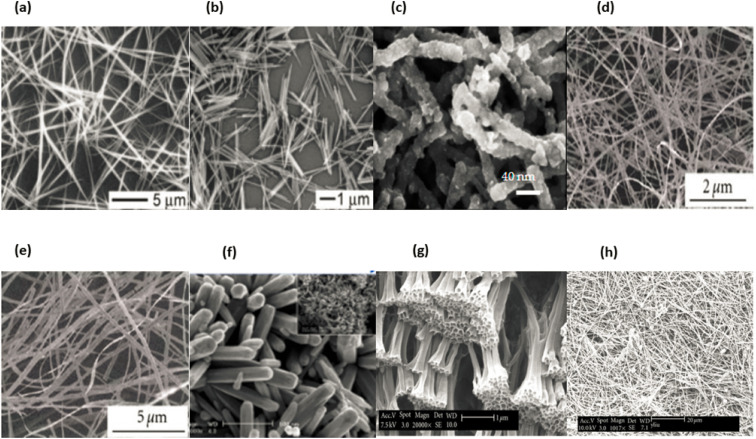
Different types of 1D nanomaterials SEM images (a) and (b) nanorods & nanowires, reproduced with permission. Copyright 2003, WILEY-VCH,^99^ (c) nanofibers,^100^ (d) and (e) nanowires and nanoribbons,^101^ (f) nanosheets,^102^ (g) nanotubes and (h) nanowires.^103^

The preceding ten years have witnessed a rise in interest for one-dimensional nanostructured materials (1D NSMs), which may be attributed to the significance of 1D nanomaterials in research and development as well as the breadth of their probable applications. It is generally agreed upon that one-dimensional nanomaterials are suitable systems for investigating a wide variety of one-of-a-kind nanoscale phenomena as well as the dependence of functional features on size and dimensions. In addition to this, it is anticipated that they will play an important part in the construction of nanoscale EED, electrical and optoelectronic devices by acting as interconnects and fundamental units. In the wake of the groundbreaking work done by Iijima, the field of 1D nanomaterials, which includes nanotubes, has attracted a significant amount of interest.^87^ 1D NSMs have significant implications for alternative energy sources, nanodevices and systems, national security, nanoelectronics, and nanocomposite materials.^88^ Nanowires, nanoribbons, nanobelts, nanotubes, nanorods, and hierarchical nanostructures are some examples of 1D nanomaterials that we provide in Fig. 13. These 1D nanomaterials have been made in laboratories operated by others.^89,89^ The number 10^−9^^90^ appears in the word nano, it is the equivalent of one billionth of any unit and outcomes in the fabrication of one-dimensional nanomaterials just like thin films. Nanoparticles have a wide range of uses in many different scientific disciplines, including chemistry, engineering, electronics, and pharmaceutics.^91^ The thickness of the monolayers or thin films might range anywhere from one to one hundred nanometers. Nanomaterials like these are utilized extensively in research and also play a role in the production of nanoscale LEDs, electronic devices and storage systems,^92^ optoelectronic, chemical, and biosensing,^93^ magnetooptics,^94^ fibre optic systems, and optical devices. One-dimensional nanomaterials are used to make essential nanoscale materials such nanotubes, nanobelts, nanowires,^95^ nano-ribbons,^96^ and hierarchical nanostructures.^97,98^Fig. 13a–h displaying the one dimension nanomaterials.

### 3.2. Two dimension nanomaterials

**Fig. 14:**
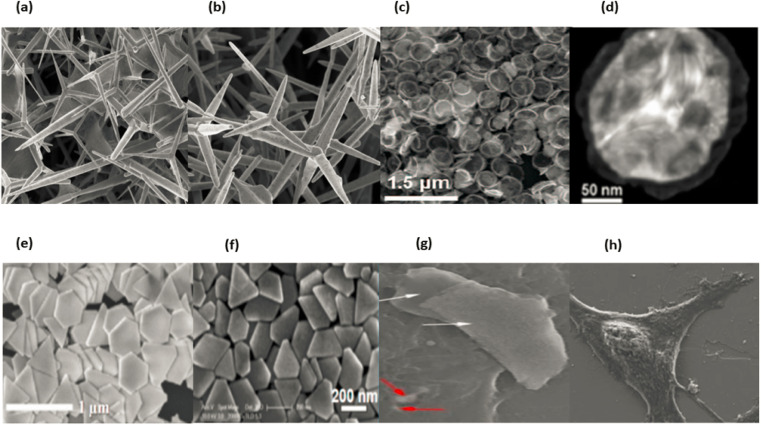
Different types of 2D Nanomaterials SEM & TEM images, (a) and (b) ref. 109, (c)–(f) ref. 110, (g) and (h) ref. 111.

Outside of the nanoscopic range of sizes, there is the possibility of two-dimensional nanostructures. In recent years, synthetic 2D nanomaterials have emerged as a primary focus of research in the field of materials science due to the various low-dimensional properties that differentiate them from the volume properties of traditional materials. This is because of the numerous advantages that these nanomaterials offer. Over the past few years, a significant amount of attention in scientific research has been directed toward the production of 2D nanomaterials in an effort to obtain 2D NSMs. Certain geometries of 2D NSMs exhibit unique shape-dependent features, which enables them to be subsequently utilized as building blocks for the construction of important parts of nanodevices.^104–106^ Additionally, 2D NSMs are particularly attractive for investigating and creating new applications in nanoreactors, templates, photocatalysts, nanocontainers and sensors for 2-dimensional structures of other materials.^107,108^ In Fig. 14, we illustrate the two-dimensional nanomaterials known as nanodisks, nanosheets, nanoprisms, nanowalls, branched structures, nanoplates and junctions (continuous islands).^89^ 2-Dimensional nanostructures are characterised by their singular form and the presence of two dimensions that lie outside of the nanometric size range. Nanomaterials with a two-dimensional structure are utilized as the fundamental building blocks for the essential parts of nanodevices.^97^ Nanocontainers, templates, nanoreactors and sensor photocatalysts for 2D structures are all examples of two-dimensional nanomaterials. Two-dimensional nanoparticles include carbon nanotubes. Fig. 14a–h displaying the two dimensional nanoparticles.

### 3.3. Three dimension nanomaterials

**Fig. 15:**
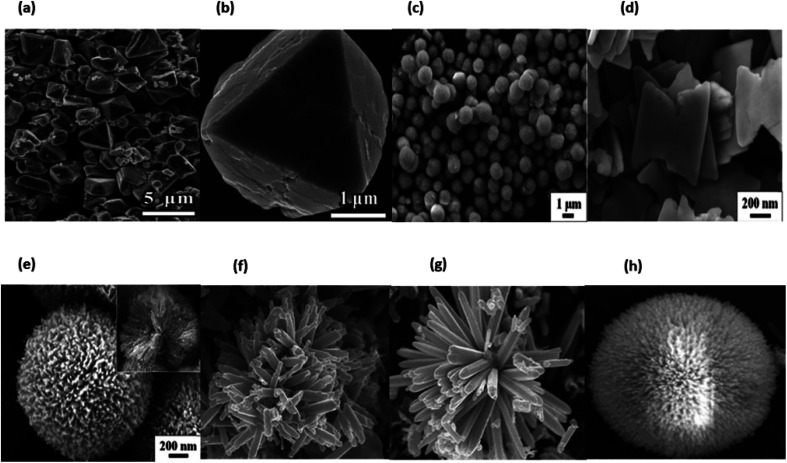
Different types of 3D Nanomaterials SEM & TEM images (a) and (b) ref. 115, (c)–(e) ref. 116, (f) and (g) ref. 117 and (h) ref. 118.

Due to the quantum size effect and other factors, 3D nanomaterials have gained a substantial amount of research attention because of their enormous specific surface area. Additionally, 3D nanomaterials have various benefits over their bulk components as a result of the quantum size effect. As a result, numerous 3D nanomaterials have been produced over the course of the past decade.^89^ It is well established that the behaviours of nanomaterials are heavily dependent on their forms, sizes, morphologies, and dimensionality, all of which are critical considerations in determining their eventual performance and applications. As a consequence of this, the synthesis of three-dimensional NSMs that have a specified structure and shape is of the extreme significance. In addition, three-dimensional nanostructures are an important material because of the many different uses that can be discovered for them in the fields of magnetic materials, battery electrode materials and catalysis.^89^ As a result of increase in surface area of these materials and their ability to offer sufficient absorption sites for all molecules that are in demand within a constrained area, there has been a surge in recent times of interest in the study of three-dimensional nanomaterials. This is one of the reasons why there has been a surge in interest in the study of three-dimensional nanomaterials.^112^ On the other hand, such three-dimensional porous materials may facilitate the transit of molecules.^112,113^ We illustrate typical 3D NMSs in Fig. 15, including nanocoils, nanoflowers, nanopillers, nanocones, and nanoballs (dendritic structures).^89^ The behaviour of nanomaterials is determined by their size, shape, morphology and dimension, which are the fundamental parameters for nanostructure performance and application.^114^ Three-dimensional nanomaterials have aroused interest in research and medical science throughout the last decade. Nanoparticles like these have a wide variety of applications, including rechargeable batteries, catalysis, and the transport of reactants and products in magnetic materials. Nanoparticles with three dimensions can be represented by examples such as quantum dots, dendrimers, and fullerenes. Fig. 15a–h displaying the three dimensional nanomaterials.

## 4. Introduction of ZnO

The most important innovations of the 21st century are the design and fabrication of nanoscale materials made of metal oxides, metals, carbon allotropes and chalcogenides. These materials are used in a vast range of fields, such as energy storage, catalysis and biosensors, conversion devices and biomedical applications. In particular, the unique physiochemical properties of semiconducting metal oxides, such as SnO_2_, ZnO, and TiO_2_, which vary depending on size and shape, have been extensively researched and exploited. One of the most stable n-type semiconducting materials for chemical and thermal applications is ZnO, which is available in a variety of forms including pellets, bulk crystal and thin film for use in everything from luminescent materials to batteries, supercapacitors and solar cells to biomedical and photocatalysis sensors. Because of their non-toxicity, large specific area, high sensitivity, high isoelectric point and good compatibility, ZnO nanostructures (nanorods, nanowires, nanorings, nanospheres and nanotubes) have recently received attention. When compared to their macroscopic counterparts, nano-sized materials have faster dissolution rates and higher solubility.^119^

In the category of semiconductor metal oxides, semiconductors in the 2–6 group at nanoscale are widely recognized for their diverse and extensive uses in a variety of fields, including solar cells, diluted magnetic semiconductors (DMS), optoelectronic devices, field effect transistors and photoluminescence appliances, to name a few.^120^

**Fig. 16:**
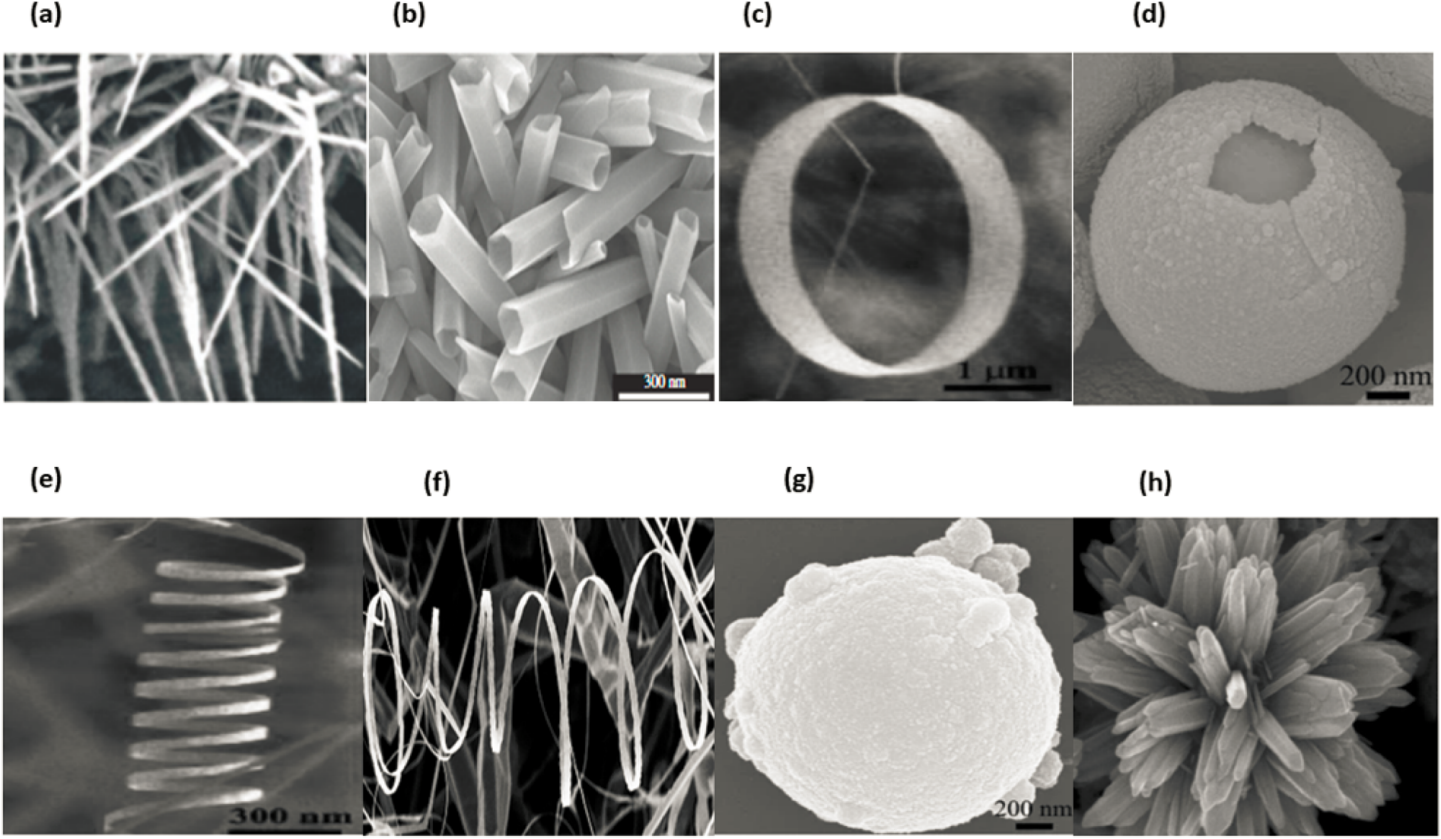
Zinc oxide structure examples: (a) wires,^146^ (b) tubes,^147^ (c) rings,^148^ (d) cages, (e) springs,^148^ (f) belts,^149^ (g) spheres^150^ and (h) flowers.^151^

In general, nanomaterials can be subdivided into one of three categories: zero-dimensional, one-dimensional, or two-dimensional. Nanostructures with zero dimensions, also known as nanoparticles with a near-unity aspect ratio or quantum dots, have found widespread application in the field of biological research.^121,122^ These nanoparticles have a two-dimensional structure and find widespread use in a variety of applications, including optical coatings and corrosion prevention. Nanomaterials can be utilised in a variety of ways, one of which is the production of thin films. One-dimensional semiconductor nanomaterials, such as nanorods, nanobelts, nanowires and nanofibres, have attracted a lot of attention in both academic research and industrial applications due to the fact that they can be used as building blocks for other types of materials. This is because of the fact that one-dimensional semiconductor nanostructures can be constructed from other types of structures.^123^ Materials with 1D nanostructures can be helpful for research into the interaction between thermal and electrical transport, dimensionality, mechanical characteristics and size reduction (or quantum confinement).^124^ In addition to this, they are extremely important in the production of nanodevices that are electromechanical, electrical, electrochemical and optoelectronic in nature, acting as interconnects and functional units respectively.^125^ It is possible to classify nano sized zinc oxide as a unique material because to the diverse structures that may be observed inside it. This material has the potential to be utilized in a broad variety of nanotechnology fields. Forms of zinc oxide can be categorised as either one-dimensional, two-dimensional, or three-dimensional. Nanorods,^126–128^ needles,^129^ helixes, rings and springs,^130^ ribbons,^131^ tubes,^132–134^ belts,^135^ wires^136–138^ and combs^139^ are among the most common one-dimensional structures. Zinc oxide can be found in the form of nanopellets, nanosheets and nanoplates, all of which are two-dimensional structures.^140,141^ Zinc oxide may produce a variety of three-dimensional structures, some of which resemble dandelions, snowflakes, flowers or even urchins on coniferous trees.^142–145^Fig. 16a–h displaying the zinc oxide structure.

ZnO possesses one of the most varied ranges of particle configurations of any material that is currently known. Due to their exceptional efficiency in photonics, electronics, and optics, ZnO nanowires are promising materials for a vast range of uses, including nanogenerators, ultraviolet lasers, light-emitting diodes, solar cells, photodetectors, photocatalysts and gas sensors. ZnO nanowires, when subjected to the appropriate light irradiation, are currently being utilized as photocatalysts for the purpose of inactivating viruses and bacteria, as well as for degrading environmental contaminants such as volatile organic compounds, dyes and insecticides.^8^ Furthermore, ZnO exhibits a vast morphological variation in nanomaterials such as nanobelts, nanotubes, nanowires, nanorods and other complex morphologies. These nanostructures can be fabricated quite easily at very low temperature, and a variety of different growth techniques for ZnO nanostructures have been documented, including chemical and physical techniques such as sol–gel deposition, cyclic feeding CVD, surfactant and capping agent-assisted growth, electrochemical deposition, hydrothermal and solvothermal growth, chemical vapour deposition (CVD) and thermal evaporation. Because of the growth procedures, disciplines and applications that were discussed above, ZnO has the potential to become one of the most significant candidates for use in future research and applications.^153^

### 4.1. Structure of ZnO

**Fig. 17:**
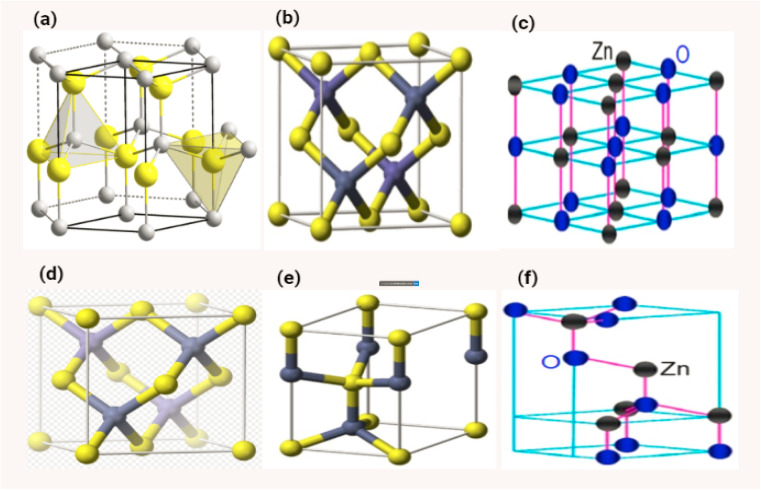
Shows the different structures of ZnO (a) and (d) ref. 158, (b) and (e) ref. 159, (c) and (f) ref. 160.

ZnO is typically hexagonal in structure. Four oxygen atoms are tetrahedrally coordinated to zinc atoms. The combination of these two ZnO structures produces perfect polar symmetry with the hexagonal axis of the zinc oxide crystal structure. These crystalline structures are responsible for ZnO-based piezoelectricity and spontaneous polarization.^154^ The cubic zinc blende structure and the hexagonal wurtzite structure are the two most common types of zinc oxide crystallisation. In typical conditions, the crystal structure of zinc oxide takes the form of the wurtzite, which has a hexagonal arrangement of its atoms. (JCPS card no. 36-1451) In order to evaluate whether or not ZnO is crystalline, one may examine the structure of hexagonal ZnO, which has the following dimensions: *a* = 0.32498 nm, *b* = 0.32498 nm, and *c* = 5.2066 nm. The value of *c*/*a*, which is approximately 1.60, is rather near to the perfect value of *c*/*a*, which is equal to 1.633 for a hexagonal cell. In Fig. 17b, the structure of ZnO can be described as a sequence of alternating planes made up of tetrahedrally connected oxygen and zinc ions that are stacked alternately along the *c*-axis. This sequence of alternating planes represents the structure of ZnO. These planes are arranged in a spiral pattern. As seen in Fig. 17e, the combination of O^2^ and Zn^2+^ results in the formation of a tetrahedral unit that lacks central symmetry.^152^ The wurtzite structure of crystalline ZnO features a hexagonal unit cell, and it either belongs to the C^4^_6v_ or *P*6_3_*mc* space group. Its lattice parameters are *a* and *c*. Lattice parameters for hexagonal unit cells are typically in the range of 3.2475 to 3.2501 for a, and 5.2042 to 5.2075 for c.^155–157^

**Fig. 18:**
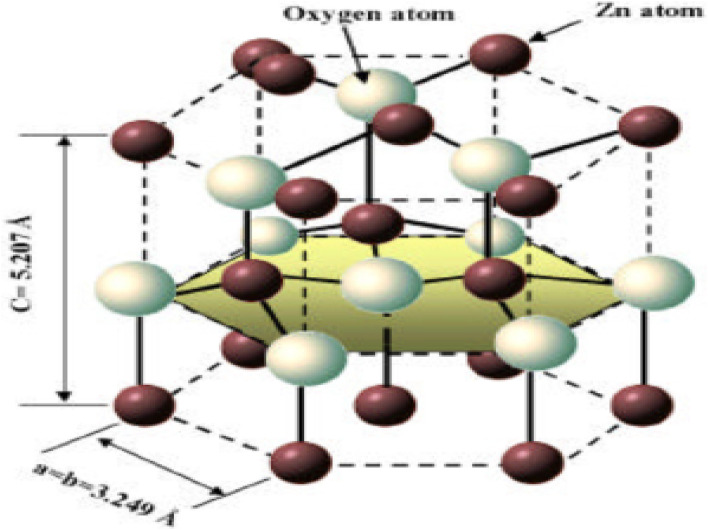
Shows a model of ZnO with a hexagonal wurtzite structure. Zn–O tetrahedral coordination is demonstrated. The atoms of oxygen are depicted as larger white spheres, while the atoms of zinc are depicted as smaller brown spheres.

ZnO has a density of 5.606 gram per cubic centimetre. As can be seen in Fig. 18, a single zinc atom is tetrahedrally connected with a total of four oxygen atoms. The piezoelectric nature of the material, which is an crucial property for the creation of micro-electromechanical systems consisting of transducers, sensors and actuators, is the cause of ZnO's noncentrosymmetric structure. This structure can be attributed to the material's piezoelectric nature.^161^ Due to the fact that it is noncentrosymmetric, it also possesses two polar surfaces on sides that are opposite one another. Each of these polar surfaces is terminated by a single type of ions (Table 1).

**Table 1:** A few basic parameters of ZnO structure.^162^

S. no	Basic parameters	Values
1	Melting point	1975 °C
2	Stable phase at 300 K	Wurtzite
3	Band gap	3.4 eV
4	Density	5.66 g cm^−3^
5	Lattice constants	*a* = *b* = 0.32495 nm, *c* = 0.52069 nm
6	Refractive index	2.01
7	Electron effective mass	0.24
8	Static dielectric constant	8.656
9	Exciton binding energy	60 meV
10	Hole effective mass	0.59

Polarity is referred to as zinc polarity when the bonds along the *c*-direction are from cation (Zn) to anion (O), whereas polarity is referred to as oxygen polarity when the bonds are from anion (O) to cation. Zinc polarity can refer to either Zn polarity or oxygen polarity; either one can be used interchangeably (Zn). This polarity is also the cause of a number of other properties that ZnO possesses, such as spontaneous polarisation and piezoelectricity. In addition to playing an important role in the creation of crystals, the formation of defects, plasticity, etching, and other processes, this polarity is also the cause of a number of other properties that ZnO possesses. It possesses both polar and non-polar surfaces, in addition to the polar ones it already has. The *c*-axis is the direction in which the polar Zn-terminated (0001) and O-terminated (0001) sides of wurtzite ZnO are oriented toward. Wurtzite is composed of ZnO, which has an equal number of atoms of both Zn and O on its non-polar (2110) (*a*-axis) and (0110) faces. The most common wurtzite ZnO crystals have these four faces arranged in a square. It has been demonstrated that the development of ZnO crystals into a wide variety of shapes is brought about by variations in the relative growth rates of different crystal facets as well as differences in the growth rates of various crystal planes. The years 1970 were the ones in which this discovery was made.^163,164^ Polar surfaces ought to be unstable from an electrostatic point of view, unless charge configurations and, as a consequence, opposite ionic charges on the surface result in spontaneous polarization and a normal dipole moment. In addition to this, it was discovered that both the surface with the coordinates (0110) and the polar surface are stable. On the other side, it has been determined that the (2110) face is less stable than its contemporaries and that it has a higher level of surface roughness than its rivals. This was found to be the case through extensive testing.^165^

### 4.2. Properties of ZnO

ZnO, once it has been developed, is considered to be a negative (n-type) semiconductor. Zinc oxide is a type of semiconductor that falls within groups 2–4 of the periodic table. The energy gap in zinc oxide is measured to be 3.37 eV. In addition, zinc oxide possesses a high binding energy. Zinc oxide has a binding energy of around 60 meV.^166^ ZnO possesses a high exciton binding energy and is very stable at high temperatures. In addition to that, it boasts a high optical gain.^167^ As a result of the characteristics that have been discussed thus far, ZnO has emerged as one of the most intriguing substances for the creation of electrical and optoelectronic devices. On the other side, due to the high binding energy of ZnO, a wide variety of photonic devices that are highly effective in their utilisation of light may be fabricated. This opens up a lot of opportunities for research and development in this area. Additionally, the large band gap of ZnO is being utilized in the research and development of short wavelength optoelectronic devices.^168^ ZnO is a type of optical material that is see-through and is optimized for use with visible wavelengths.^169^ Numerous research organisations have examined the ZnO's unique features. This results in an improvement of ZnO's electrical and optical characteristics. Numerous other features of ZnO enable a diverse range of uses. These include light-emitting diodes, photovoltaics, microelectromechanical systems and photodetectors.^170–173^ ZnO is an incredibly versatile material with semiconducting, pyroelectric and piezoelectric characteristics. ZnO is a material that exhibits a wide variety of nanostructures, significantly more than any other nanomaterial, including carbon nanotubes.^174–176^

#### 4.2.1. Optical properties of zinc oxide

The way in which a substance reacts when illuminated by light is what establishes its optical characteristics. Based on their physical characteristics, such as their vibrational and electrical states, as well as the presence and nature of impurities and defects in the material, semiconductor substances have contributed a great deal to our understanding of a wide range of topics, providing a wealth of information along the way. This knowledge has been gleaned from the study of semiconductor materials. The extrinsic and intrinsic qualities of a material are what designate it as a semiconductor. Excitonic causes related to the Coulomb attraction, in addition to the interaction between electrons in the conduction band and holes in the valence band, are the fundamental building blocks for the intrinsic properties of semiconductors. These characteristics are known as the electronic band structure. The dopants and defects that are injected into the semiconductor are what govern its extrinsic optical properties. These dopants and imperfections form discrete electronic states between valence band and conduction band.^177^ Investigations into an optical transition in a zinc oxide semiconductor have been carried out utilizing a variety of methodologies, including transmission photoluminescence, cathode luminescence, reflection, optical absorption, and others. The photoluminescence method is one of these methods that has seen widespread usage in the process of determining the optical behaviour of zinc oxide structures. The photoluminescence spectra of various zinc oxide nanostructures include UV emission in addition to one or two bands of visible emission that are brought about by vacancies, antisites, interstitials, defects, and complicated defects.^178,179^ The zinc oxide material has a band gap of 3.37 eV and an exciton energy of 60 meV when it is at ambient temperature. Due to the fact that this material has a higher exciton energy value than GaN, it is capable of emitting excitons in an effective manner at room temperature and below low excitation energy (25 meV). As a direct result of this, zinc oxide is currently considered to be one of the most promising photonic materials in the blue-ultraviolet region.^180^ The optical properties of zinc oxide nanorods have been studied using photoluminescence spectroscopy, which provides information about the band gap, defects, and crystal features.^180,181^ Zinc oxide nanorods show a near-band edge for UV emission and a broader band emission due to deep level defects when subjected to photoluminescence study at ambient temperature. A single emission at UV emission (from 3.236 to 3.307 eV) has been seen in zinc oxide nanorods with lower impurity concentrations, and deep level emissions have been seen in these nanorods as well.^182^ Near-band edge and deep level emissions band emissions in the UV and visible ranges are attributable to defects in the zinc oxide nanostructure such as oxygen vacancy, oxygen interstitial, zinc vacancy, zinc interstitial and extrinsic contamination.^180,182^ The optical quality of various zinc oxide systems can be assessed by comparing the relative intensity of near-band edge and deep level emissions emission. Thus, the optical quality of zinc oxide is given by the ratio of near-band edge emission intensity to deep level emissions emission intensity (*I*_NBE_/*I*_DLE_). The high (*I*_NBE_/*I*_DLE_) number indicates a deep level defect with lesser concentration.^182^ Different type of ZnO doped optical properties graphs are displayed in the Fig. 20 (a–f).

#### 4.2.2. Magnetic properties of zinc oxide

**Fig. 19:**
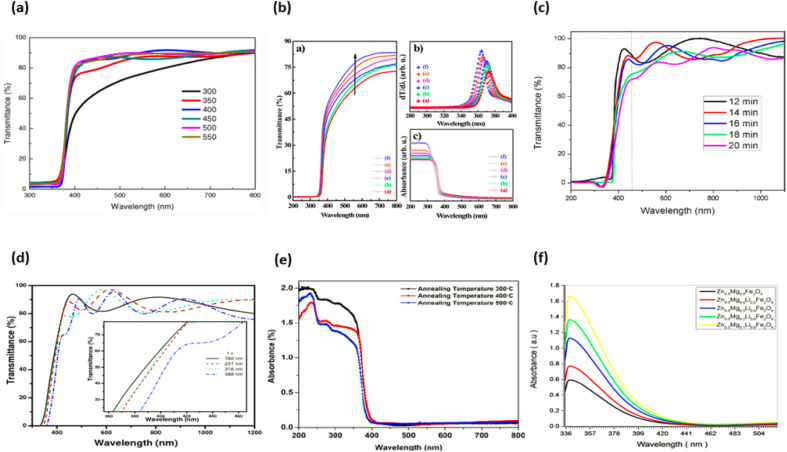
Typical ZnO doped optical properties graphs (a) ref. 183, (b) ref. 184, (c) ref. 185, (d) ref. 186, (e) ref. 187, (f) ref. 188.

The orientation of electron spins in the magnetic semiconductor host lattice is used to categorise the various types of magnetic semiconductors. On the basis of this alignment, semiconductors can be divided into the following three distinct categories: (a) magnetic semiconductor, (b) DMS semiconductors and (c) semiconductors that do not exhibit magnetic behaviour, as seen in Fig. 19a–c. As seen in Fig. 19a, magnetic semiconductors are constructed solely out of the periodic alignment of magnetic components. As can be seen in Fig. 19b, DMS are composite materials consisting of magnetic components and nonmagnetic semiconductors. As can be seen in Fig. 19c, nonmagnetic semiconductors do not have any magnetic impurities present in the host lattice of the semiconductor. Doping various transition or rare earth ions into nonmagnetic semiconductors, such as Cu, Ni, Sm, Co, Cr, Eu, Fe, Mn, Gd, and so on, is one method for producing DMS (Fig. 19b).

**Fig. 20:**
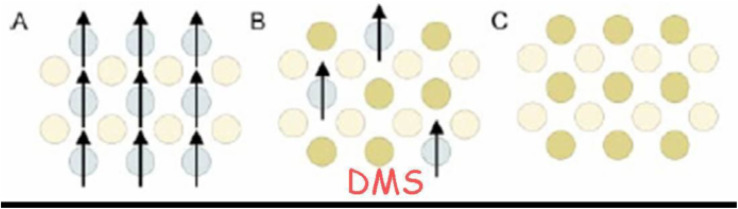
Types of semiconductor (a) a magnetic semiconductor, (b) a dilute magnetic semiconductor and (c) a non-magnetic semiconductor^162^.

Doping is the technique of inserting impurities on purpose into an intrinsic semiconductor in order to affect the material's physical properties. Doping is also known as doping an intrinsic semiconductor. A great number of research reports were distributed all at once *via* DMS^154–159,189^. There have also been reports of attempts to doped semiconductor nanocrystals^160,161^. There is a growing interest in studying the fundamental characteristics of DMS in various nanostructures for spintronics applications^158–160,162^. The introduction of transition or rare earth ions into semiconductors results in the formation of these materials. Because the d and f shells of transition or rare earth ions are only partially full, these doping elements have electrons that are not connected with another atom. This allows for greater doping efficiency. One of the bands in transition metals such as manganese, copper, cobalt, and nickel is only partially full or just over half-filled at most (up or down spins). The ions of the transition metal are almost always substituted for the cations that are originally present in the host semiconductor. Doping manganese into zinc oxide, for example, causes the element to offer its four s^2^ electrons to the s–p^3^ bonding and causes a Mn^2+^ charge state to be created in the tetrahedral bonding. This is because doping manganese causes the element to give its four s^2^ electrons to the s–p^3^ bonding. In order to determine the tetrahedral bonding, the d bands of the transition metal hybridise with the VB bands of the host (O-p bands in zinc oxide). Because of this hybridization, the interface between the locally organised carriers in the host valence band and the three-dimensional spins is replaced, which results in the sample having a local magnetic moment. When it comes to defining the magnetic properties of materials that have been doped with transition metals, the degree of doping in the carrier density, the crystal, and the quality of the crystal all play a part.

Dulub *et al.*^163^ provided the impetus for thinking about semiconductor oxides, specifically zinc oxide, in the context of spintronics. According to the predicted mean field theory, common diamagnetic semiconductors with five atomic percent Mn doped and a hole quantity of 3.5 × 10^20^ cm^3^ would have a high Curie temperature. In the case of zinc oxide and GaN, simulations show that the Curie temperature exceeds 300 K.^163^ Because the Zener model suggests that magnetic properties can be modified by modifying the carrier concentration in the materials, the character of the carrier is an important part of the model that must be taken into consideration. In the beginning, the substitution integral parameter suggested that p-type materials would be ideal candidates for high Curie temperatures. Additionally, the density of states in the valence band is higher than the density of states in the conduction band. Because it is impossible to produce p-type zinc oxide, Dietl's theory does not apply to zinc oxide, which is an element.

Li *et al.*^164^ proposed a pattern that demonstrates the dominance of defect states on DMS ferromagnetism properties.

They claim that donor defects are responsible for covering up a significant amount of doping substance as well as the establishment of a contaminated band. In the case of type zinc oxide, these donor defects can take the form of zinc interstitials or oxygen vacancies. This contaminated band is capable of interacting with the local magnetic moment if the bound magnetic polaron is made significantly larger. Within this radius, the magnetic dopants will interact with the bound carrier, and they will be able to align their spins in each bound magnetic polaron so that they are parallel to one another. In order to obtain both ferromagnetism and penetrating ferromagnetism in the DMS, the magnetic polarons that are coupled to the electrons in the material must be stacked one on top of the other to create a chain that runs the length of the material. MS nanocrystals are remarkable materials that incorporate quantum confinement effects as well as magnetic features due to the system's DMS composition. Some artificial problems are involved in the direct exchange of cations/anions of host material *via* dopant ions in nanocrystals.^165,166^ A significant barrier that needs to be surmounted is the creation of nanocrystals that have dopant ions incorporated continuously throughout the lattice of the host substance. The utilization of nanocrystals that have a high surface area to volume ratio has begun to promote impurity separation to the surface of the nanocrystals during a process known as “self annealing” in the core. This process takes place in the core. As a consequence of this, dopants are probably just sitting on the surface, which results in a high level of entrapment. Nevertheless, the production method for generating doped nanocrystals serves a crucial function in the overall process. Several papers^162,167,168^ propose successful dopant integration in host materials. Doping a very small amount of rare earth or transition atoms is the primary technique that is utilized in the process of initiating magnetism in ZnO. There are still some points of contention regarding the substitutional insertion of transition or rare earth elements in host materials and the attainment of ferromagnetism in doped host materials, both of which have been the subject of extensive research. The ferromagnetism that was explored in the host semiconductor could have been induced by the inherent magnetism of the semiconductor itself, as well as its precipitates, or the secondary magnetic phases of transition metals. If DMS is researched in a methodical manner by correlating all of its attributes, then the controversy over the presence of magnetic properties can be settled once and for all.

**Fig. 21:**
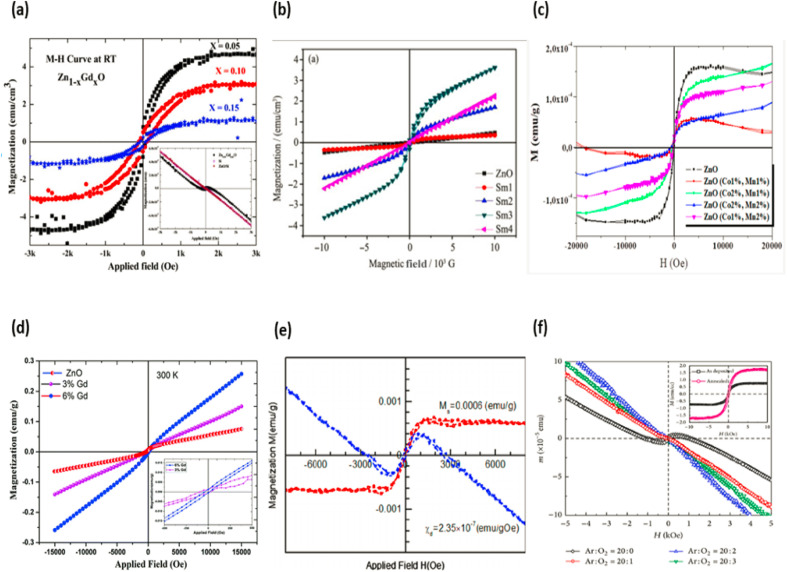
Typical ZnO doped magnetic properties graphs (a) ref. 190, (b) ref. 191, (c) ref. 192, (d) ref. 193, (e) ref. 194, (f) ref. 195.

A great deal of curiosity has been ignited as a result of the finding that metal oxide nanocrystals exhibit ferromagnetism at normal temperature. When compared to the equivalent bulk material, nanocrystals have a high surface-to-volume ratio; hence, changes in nanocrystal size have the greatest influence on the surface effects. The influence of voluntary surface spins on saturation magnetization provides evidence that this function plays a crucial role in magnetic properties. In their bulk and nanostructure forms, metal oxides such as HfO_2_, ZnO, and Al_2_O_3_ exhibit diamagnetic and ferromagnetic magnetic properties, respectively.^169^ Interactions between localised electron spin moments and oxygen vacancies at nanocrystal surfaces are thought to be the cause of ferromagnetism in nanocrystalline materials.^169^ Ferromagnetic behaviour was seen in chemically produced zinc oxide nanocrystals that were capped by a variety of capping agents when the samples were allowed to reach room temperature. Spin polarization can be facilitated by the alteration of the surface charge state by coupled ligand.^152^ According to the findings of this study, the magnetic properties of nanocrystals are not only associated with the presence of magnetic ions, but they are also highly supported by the presence of surface defects. Additionally, the presence of magnetic ions is associated with the magnetic properties of nanocrystals. In addition to this, the magnetic properties are connected with the presence of magnetic ions, which explains why they have these characteristics.^152^ Different type of ZnO doped magnetic properties graphs are displayed in the Fig. 21 (a–f).

### 4.3. ZnO and their structural properties

The variation of ZnO nanostructures is determined by the growth mechanism, the growth method, the synthesis conditions, and the type of substrate. Nanowires, nanorods, nanotubes, nanocolumns, nanorings, nanobelts, nanosheet networks, nanoribbons, nanoflowers, hollow micro- and nanospheres and nanocombs are among the nanostructures.

#### 4.3.1. ZnO nanorods and nanowires

**Fig. 22:**
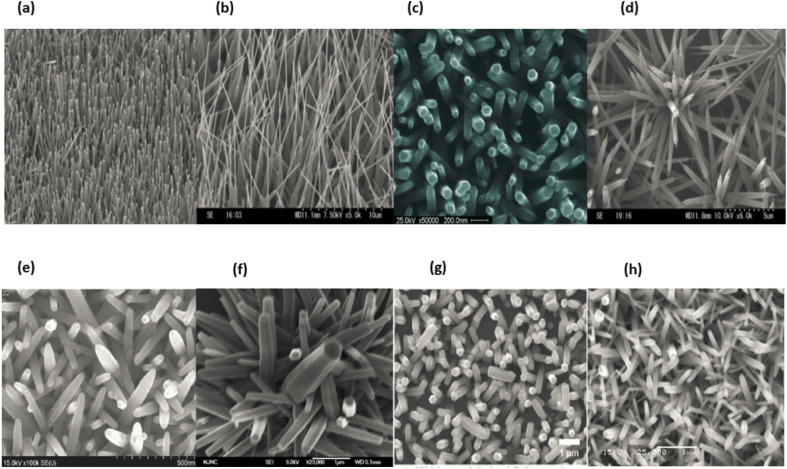
ZnO nanowires and rods SEM images (a) and (b) ref. 196, (c) ref. 197, (d) ref. 196, (f) ref. 198, (g) ref. 199, (h) ref. 200, (e) ref. 201.

One-dimensional nanostructures such as nanorods and nanowires are gaining popularity due to the multiple applications they have in photovoltaic systems, nanoelectronics, chemistry, and biosensors. These nanostructures are comprised of a single layer. In addition, the 1D nanostructures are a good candidate for the production of effective optoelectronic nanodevices due to their many important characteristics, such as a direct band-gap and a significant exciton binding energy. This makes the 1D nanostructures a good candidate for the production of efficient optoelectronic nanodevices. There have been a significant number of articles written about the synthesis of zinc oxide nanorods and nanowires, as well as their characteristics and the growth mechanisms behind them. There are two primary growth methods that have been described for the creation of zinc oxide nanowires and nanorods through a gas phase process. These approaches are as follows: vapor–liquid–solid,^170,171^ and vapor-solid.^172–174^ As nucleation sites for the generation of one-dimensional nanostructures, metal nanoclusters or metal nanoparticles have been utilized in the VLS mechanism, which is a process that is supported by a catalyst. Metal is the material that both of these nanoclusters and nanoparticles are composed of. The production of alloy liquid droplets takes place as a result of the gaseous reactants interacting with the catalytic particles throughout this process. The formation of 1D nanostructures is significantly aided by the participation of these droplets. The formation of precipitation begins when a droplet of liquid gets supersaturated with the medium from which it originated. If the conditions are favourable, the amount of precipitation that falls will continue to increase over the course of time, which will result in the construction of connected structures that are one-dimensional. During the process of creating 1D nanostructures, a number of different metal catalyst components, including gold, tin, copper, and cobalt, are utilised as catalysts. On the other hand, in order to successfully carry out the VS mechanism, the utilisation of a catalyst is not required in any way. When it comes to the process of generating 1D nanostructures, it is generally agreed upon that one of the most significant components is mastering the ability to regulate the level of supersaturation present. This is due to the fact that the degree of supersaturation is what is responsible for determining the primary growth morphology. For whiskers growth, a low degree of supersaturation is required; for bulk growth, a medium degree is necessary; and for powder growth, a high degree of supersaturation is necessary. The source materials are vaporised at a high temperature in the usual vapor solid process, and then they are quickly condensed onto the substrate in a zone that is characterized by a low temperature. This results in the formation of a vapor solid. This takes place in an area where the temperature is lower than the point at which vaporization can take place. Condensed molecules give rise to seed crystals after the first stage of the condensation process is complete. These seed crystals are put to use as nucleation sites for the later phases of the process of developing nanostructures. The specific vapor solid approach has been established for the purpose of manufacturing a wide variety of ZnO nanostructures in order to fulfil demand. Fig. 22a–h displaying the ZnO nanowires and rods.

#### 4.3.2. ZnO nanotubes

ZnO nanotube arrays are almost always created by dissolving the centre of previously made nanorods. Electrochemical dissolution is a viable option.,^202^ Chemical dissolution is more prevalent, though. Zinc oxide is amphoteric, which means it may dissolve chemically in either an acidic (HCl) or a basic (KOH) solution.^203^ The selective decomposition of the nanorods' centres while leaving the lateral faces unaffected by the process can be explained in two different ways. To begin, the metastable plans for (0001) zinc oxide have a higher surface energy than the lateral plans, which are thus more stable.^204,205^ Second, the (0001) designs have more defects, which makes them more likely to break.^204^ To the dissolving solution, Wang *et al.* added a surfactant (cetyl trimethylammonium bromide).^206^ This molecule is well known for being bound to the (1000) plans and safeguarding the wall of the 1D structure throughout the process of nanorods dissolving into nanotubes. This is why it has received so much attention.^207–210^ Ammonia solution is used as an etching agent.

All of the aqueous solutions were prepared with deionized water that had been acquired from Sigma-Aldrich. The resistivity of the water was 18 Ω cm. All of the chemicals that were utilised in this work were of analytical quality and did not require any additional purification prior to their usage. The manufacturing of ZnO nanotubes is a process that takes place over two stages. In the initial step of the process, the substrate, which was a piece of clean room paper measuring 50 mm by 50 mm, was washed in deionized water and then allowed to dry in the air. After that, the substrate was heated for an additional twenty minutes at a temperature of one hundred degrees Celsius in order to evaporate any trace amounts of moisture that could have been present in the paper. The paper substrate, on the other hand, has a large capacity for absorbing water, which makes it susceptible to wetting and limited in its ability to withstand low temperatures for extended periods of time. As a consequence of this, a wetting and chemical barrier layer is essential in order to shield the paper substrate from the effects of being exposed to water and chemicals.^211^ This layer needs to be able to act as a barrier against chemicals and moisture, in addition to having mechanical and dielectric properties that are satisfactory. The deposition of such a barrier layer on the substrate can be accomplished using a various of methods, including sputtering, evaporation, and chemical vapour phase deposition, to name a few.^211^ In addition, because these processes require a large number of intricate stages, we opted to avoid them in favour of a method that was both straightforward and highly effective. This method involved applying a protective layer to the paper substrate in order to achieve passivation or chemical resistance. For this purpose, we utilised the advanced electronics cyclotene 3022-46 resin that is manufactured by Dow Chemical Company USA. This resin is a polymer that can be used for wafer-level applications that require a thin layer, and it can do so successfully. During the synthesis process, the surface roughness and damage may be reduced thanks to this change of the surface, which may also help. In addition to this, it has the potential to assist in enhancing the alignment and homogeneity of ZnO nanotubes on the paper substrate.^212^ After applying a layer of cyclotene by spin coating to the surface of the paper substrate, we baked it in a vacuum for fifty minutes at a temperature of one hundred degrees Celsius. After that, the substrate was roasted in the oven for approximately thirty minutes at a temperature of one hundred sixty degrees Celsius. After that, a spin coater was used to apply a seed layer to the substrate at a speed of 2100 rpm for approximately one minute. This step served to provide nucleation sites for the creation of ZnO nanorods. This method was carried out a total of five times in order to ensure adequate coverage.

ZnO nanoparticles were used to construct the seed layer. These nanoparticles were produced by achieving a concentration of 0.01 M in methanol by diluting zinc acetate dehydrate, which has the chemical formula (C_4_H_6_O_4_Zn·2H_2_O). After that, the solution was brought up to a temperature of sixty degrees Celsius. A second solution of KOH in methanol with a concentration of 0.03 M was dropwise added to the first solution while it was continuously stirred at a temperature of 60 °C for two hours. ZnO nanoparticles have diameters that ranged anywhere from 5 to 10 nanometers.^213^ After maintaining a temperature of 180 °C on the substrate for a period of thirty minutes, it was eventually possible to consolidate the seed layer. This was made possible after the substrate was heated. Following that, the temperature of the substrate was permitted to gradually recover to that of the surrounding environment. We chose a method for developing the ZnO NRs that required a temperature that was on the lower end of the spectrum. Zinc nitrate hexahydrate [Zn(NO_3_)_2_·6H_2_O] and hexamethylenetetramine (C_6_H_12_N_4_) were mixed in equal amounts in DI water and kept under continuous magnetic stirring for 30 min in order to obtain a consistent growth solution. This was done in order to obtain a consistent growth solution. This was done in order to obtain a growth solution that was consistent throughout. After that, the paper substrate, which had been preheated, was immersed in the solution and heated at a temperature of eighty degrees for a period of five hours. Following that, it was cleaned with DI water to remove any residuals that may have been on the surface, and after that, it was dried at room temperature in the air.

**Fig. 23:**
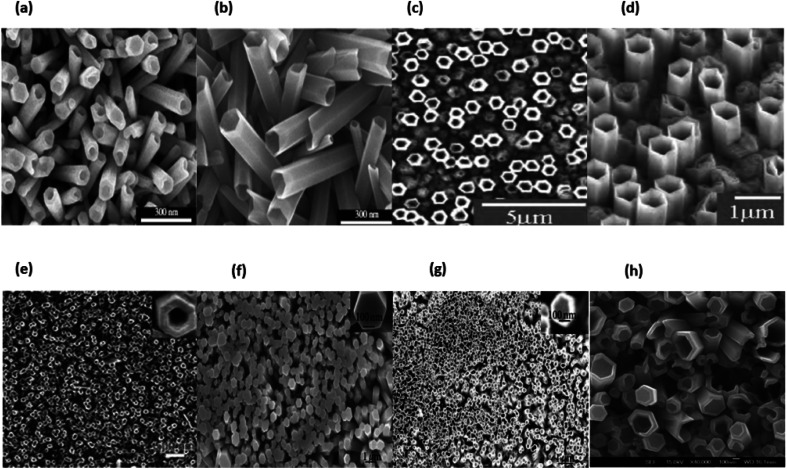
Typical SEM images of ZnO nanotubes (a) and (b) ref. 217 (c) and (d) ref. 218 (e) ref. 219 (f) and (g) ref. 220 (h) ref. 221.

The second step was to obtain the ZnO nanotubes, which we did using a process that involves chemical etching to convert ZnO NRs to NTs.^214^ This approach has been effectively used by many research groups to produce zinc oxide NTs.^215,216^ In order to do this, the ZnO NRs that were located on the paper substrate were chemically etched into ZnO NTs by placing them in an aqueous solution of KCL at a temperature of 80 °C for several hours. After that, the substrate was taken away and given a thorough cleaning with DI water in order to remove any residuals that might have been on the surface. The last step was to hang it up outside so it could dry. We used scanning electron microscopy with a 12 keV energy setting and transmission electron microscopy with a 200 keV energy setting to explore the surface morphologies and diameters of the ZnO nanotubes that were formed. An X-ray diffractometer was used with Cu K radiation, a wavelength of 1.54178 Å, 40 keV, and 100 mA for the aim of determining the crystal structure of the final products and classifying them into their respective phases. This was accomplished by using the instrument. An energy-dispersive X-ray spectroscopy that was linked to a scanning electron microscope and operated at 20 keV was used to investigate the evidence for the purity and elemental composition of the as formed ZnO nanotubes. The charge-coupled device camera, which was cooled using nitrogen, was used to carry out the measurements of CL. The luminescence was collected by a parabolic mirror, and it was then scattered by a monochromator of 0.55 metres in length and fitted with a grating measuring 600 mm^−1^. Fig. 23a–h displaying the ZnO nanotubes.

#### 4.3.3. ZnO nanobelts and nanorings

**Fig. 24:**
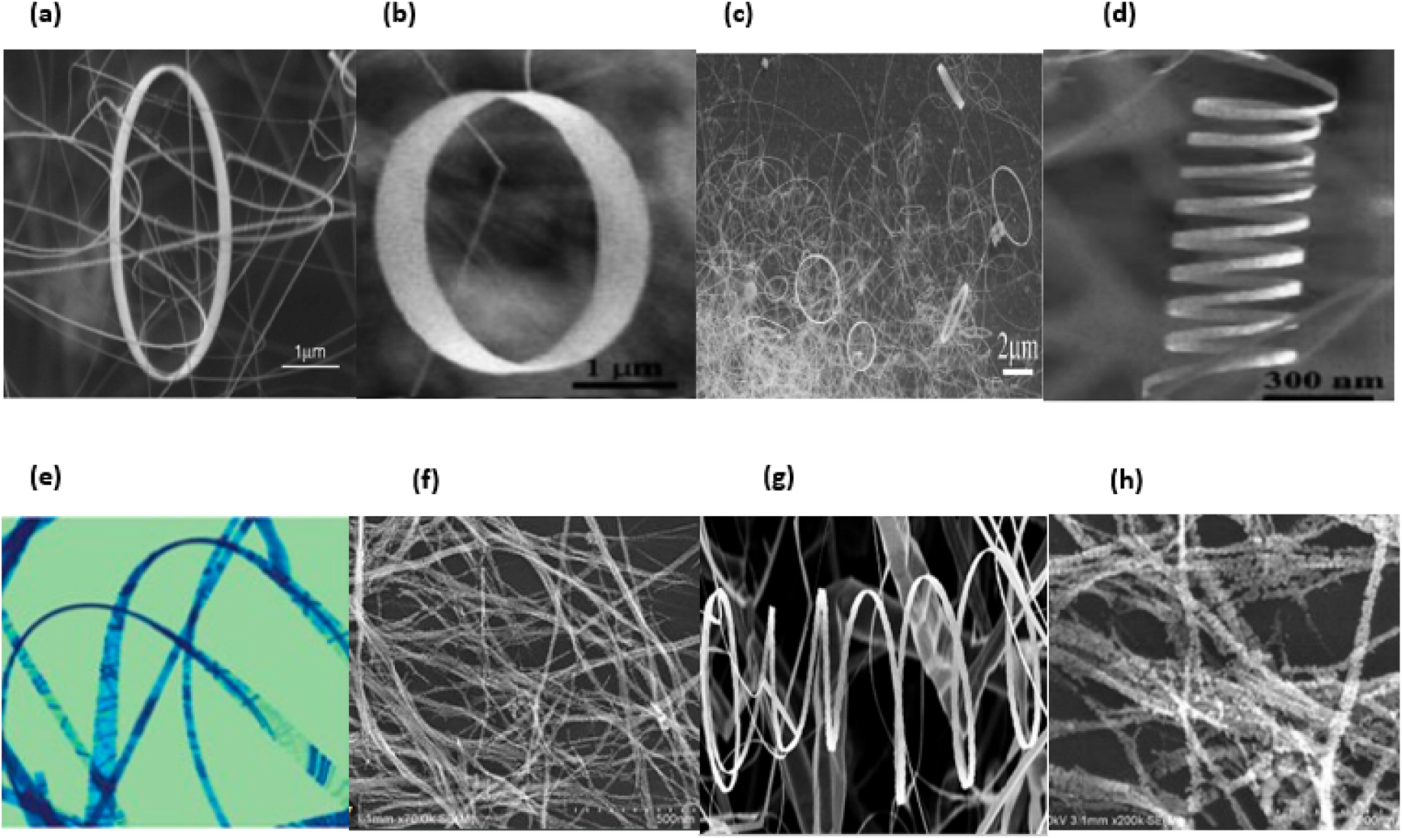
Typical SEM images of ZnO nanorings are shown in (a) ref. 229, (b) ref. 148, (c) ref. 230, (d) ref. 148 and ZnO nanobelts are shown in (e) ref. 231, (f) ref. 232, (g) ref. 149, (h) ref. 232.

The belt-like nanostructure morphology is a structural characteristic that is maintained by functional oxides with a variety of different crystallographic structures. In reality, three different architectures can be used to generate ZnO nanobelts (NBs).^222^ Planar defects can be used to grow one of these structures. ZnO NBs can be used in a variety of ways. ZnO nanorings are a remarkable type of ZnO morphology that can be produced, in addition to ZnO nanobelts, by ZnO nanobelts.^223^ Planar defects are required for the development of nanorings, according to microscopic research.^224^ Twins, interstitial stacking layers and conventional stacking faults created by impurity atoms are all examples of planar defects.^225^ Using indium as a dopant element, Wang's team was able to create ZnO nanorings.^222^ They also demonstrated that the introduction of planar defects within ZnO NBs, such as inversion domain borders, may be caused by the doping of indium ions. They came to the conclusion that the polarity of the NBs was in no way affected by the IDBs, regardless of whether they were coupled head-to-head or tail-to-tail. This was the finding that they came to. Because of the long-range electrostatic interaction between the surface polar charges on the two sides, the development of a nanoring was initiated by circularly folding a Nanobelt, and loop-by-loop winding of the nanobelt generated a full ring. This interaction was caused by the fact that the nanobelt had surface polar charges on both of its sides. This interaction was brought about as a result of the fact that the surface polar charges on both sides are charged in the opposite direction. The fact that a nanobridge can be folded into a nanotube allowed for the successful completion of this interaction. They came to the conclusion that indium would be the best material to use as the doping agent due to the significance of indium doping in the semiconductor industry. Furthermore, many research groups, including our own, have been focusing their efforts over the past few years on the development of indium-doped zinc oxide nanostructures. Tin is an important doping chemical that, in addition to indium, has the potential to open up new applications for zinc oxide.^226^ It is generally knowledge that the band gap of ZnO can be adjusted to a more desirable value by alloying the material with another substance that possesses a band gap that is different. This causes a change in the wavelength of exciton emission to occur as a result. Because the inclusion of tin oxide, which has a greater band-gap than ZnO (3.6–3.97 eV), results in a widened band-gap, the ZnO/SnO_2_ structure produced by alloying ZnO with SnO_2_ could be a possible contender for future optoelectronic devices. This is due to the fact that the inclusion of SnO_2_, which has a greater band-gap than ZnO. In addition to this, when compared to undoped zinc oxide nanowires, the field emission properties of Sn-doped zinc oxide nanowires are significantly improved, and the resistance decreases as the amount of Sn present in the nanowires increases. ZnO's band-gap ranges between 3.6 and 3.97 ev, whereas SnO2's band-gap is between 3.6 and 3.98 ev.^227^ Sn can act as a doping material in ZnO NBs, causing the production of planar defects, in addition to its impacts on the optical band gap and better electrical characteristics.^228^ As a direct consequence of this, it will be possible to produce zinc oxide nanorings using tin (Sn) as the dopant material, which is something that has never been accomplished previously. On the other hand, research has not yet been conducted to determine how the concentration of tin influences the formation of zinc oxide nanorings. In addition, the vapor–liquid–solid process, which is considered to be one of the most important ways for producing one-dimensional nanostructures, has not yet been employed to create zinc oxide nanorings. Fig. 24a–h displaying the ZnO nanobelts and nanorings.

#### 4.3.4. Hollow ZnO nano- and micro-spheres

Utilizing carbonaceous saccharide microspheres of variable diameters as templates has resulted in the development of a straightforward and generic process that is capable of producing zinc oxide hollow microspheres with a number of shells that can be adjusted. This method is also very successful. It was revealed that the triple-shelled zinc oxide hollow spheres with a large surface area displayed the highest levels of photocatalytic activity. This was discovered when the photocatalytic capabilities of the as-synthesized products were assessed by degrading methyl orange (MO) dye. In addition to this, research was conducted to determine the mechanism behind the production of multiple shelled zinc oxide hollow spheres and the rationale behind their high photocatalytic activity.^233^

Every one of the elements was of reagent-grade quality, and they were all utilized in their natural state. In this experiment, the metal precursors were zinc nitrate hexahydrate (Zn(NO_3_)_2_·6H_2_O) and carbonaceous saccharide microspheres served as the sacrificial templates. The following explanation provides an outline for the typical production of single-shelled ZnO hollow microspheres. In accordance with what was previously stated, carbonaceous microspheres were produced using the emulsion polymerization of sugar in hydrothermal circumstances.^234,235^ Adjusting the amount of sugar solution used and the amount of time the reaction is allowed to run can change the width of the carbon spherules that are formed. The microspheres of carbonaceous saccharide were given multiple washings in deionized water and ethanol at a concentration of one hundred percent until the filtrate became transparent.

**Fig. 25:**
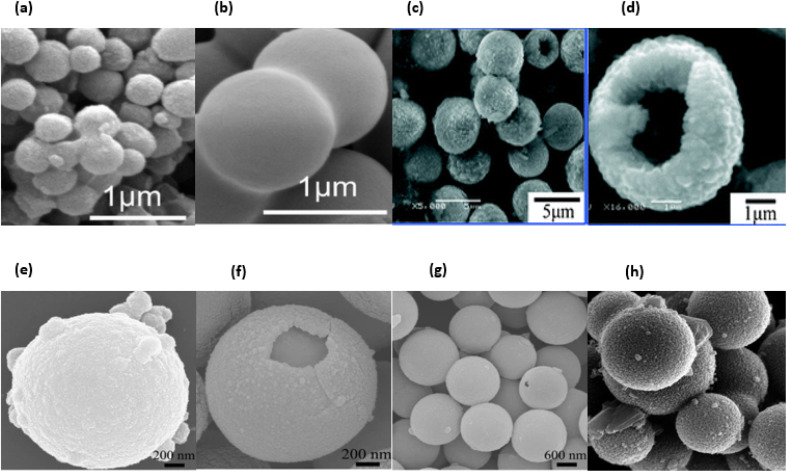
Typical SEM images of ZnO nanospheres and microspheres are shown in (a) and (b) ref. 236, (c) and (d) ref. 237, (e)–(g) ref. 150 and (h) ref. 238.

With the assistance of ultrasonication, freshly manufactured carbonaceous microspheres (0.5 g) with diameters of 500 nm were dispersed throughout a solution of 1.5 M zinc nitrate (water/ethanol = 1 : 3, v/v, 25 mL). Following the completion of a total of 0.5 h of ultrasonic dispersion, the suspension was then aged for 8 h in a water bath maintained at a temperature of 60 °C. After that, the suspension was dehydrated in an oven at a temperature of 80 °C for 12 h, after which it was vacuum filtered, rinsed several times with deionized water, and then filtered again. The resulting black composite microspheres were heated to 350 °C for one hour, then gradually brought up to 450 °C in air at a rate of one degree per minute, and eventually held at 450 °C for two hours. This was done so that the templates could be removed. The ZnO hollow microspheres with a single shell were produced as a white powder by-product when the tube furnace was allowed to naturally cool to room temperature. A procedure that is quite similar to this one was used in the production of double- and triple-walled ZnO hollow microspheres. Burning was one of the steps in the process that resulted in the production of ZnO nanoparticles. In a nutshell, 5 mL of deionized water were used to dissolve 3 g of Zn(CH_3_COO)_2_·2H_2_O and 1 g of CO(NH_2_)_2_, and then NH_3_ was added after that. The water was added in a very careful and methodical manner drop by drop until the solution became extremely thick gel precursors. This process took quite some time. The resultant viscous gel precursors were immediately heated to 500 °C, where they spontaneously ignited to generate white ZnO particles. This step was repeated until the desired amount of ZnO had been produced. Fig. 25a–h displaying the ZnO nano and microspheres.

#### 4.3.5. Star- and flower-shaped ZnO nanostructures

Using a method that is based on a solution and performed at a low temperature, sulphur doping of hexagonal ZnO nanowires using thiourea (SC(NH_2_)_2_) produces hexagram-shaped ZnO nanostructures (“nanostars”). According to scanning electron microscopy, the amounts of sulphur doping have a significant impact on the morphology of the nanostructure when viewed in cross-section (SEM). According to the calculations of the density functional theory, the transformation from hexagonal nanowires to nanostars takes place as a result of sulphur atoms preferentially bonding to the vertices of hexagonal structures while the structures are developing. The observed hexagram structure is most likely the result of the resultant shift in the local chemical environment. X-ray photoelectron spectroscopy and photoluminescence spectroscopy were utilized to demonstrate the presence of sulphur in the nanostars. The involvement of sulphur in the formation of nanostars was validated in control tests using the sulfur-free counterpart urea (OC(NH_2_)_2_).

The processes developed by Greene and Pacholski for making ZnO nanowires in solution were followed to make the nanowires. In the first step of this procedure, solutions of zinc acetate dihydrate [(CH_3_CO_2_)_2_Zn·2H_2_O (Fluka, assay 99.5%)] and sodium hydroxide [NaOH (Fisher Chemical, assay = 98.6%)] were prepared in methanol at concentrations of 0.01 mol L^−1^ and 0.03 mol L^−1^, respectively. The mixture of 13.68 mL of a solution containing 0.03 mol L^−1^ of NaOH and 26.32 mL of a solution containing 0.01 mol L^−1^ of (CH_3_CO_2_)_2_Zn·2H_2_O was then stirred for two hours at a temperature of 60 °C. ZnO seed crystals were produced by applying a drop-coating of the solution that had been produced to a silicon substrate, then rinsing the substrate with methanol and blow-drying it with air. This method of drop-coating was carried out a number of times. After that, the substrate was annealed for twenty minutes at a temperature of three hundred and fifty degrees Celsius in order to form ZnO seed crystals. After placing the substrate in an aqueous solution that contains 0.025 mol L^−1^ of zinc nitrate [Zn(NO_3_)_2_·*x*H_2_O (Alfa Aesar, assay = 99%)] and 0.025 mol L^−1^ of hexamine (hexamethylenetetramine) [(CH_2_)_6_N_4_ (Alfa Aesar, assay = 98%)], heat the mixture at 90–95 °C for The ZnO nanostars that were used for this study were produced by hydrothermally growing them with varying concentrations of a thiourea [SC(NH_2_)_2_ (Alfa Aesar, test = 99%)] doping solution. This method was used to explore the nanostars' properties. The amounts of thiourea were changed (0.025 mol L^−1^, 0.05 mol L^−1^, 0.1 mol L^−1^, 0.2 mol L^−1^, and 0.5 mol L^−1^), and a control experiment was performed using urea at a concentration of 0.1 mol L^−1^ [OC(NH_2_)_2_ (Acros Organics, assay = 99%)]. In every single experiment, 10 mL of each reactant solution were utilized.

**Fig. 26:**
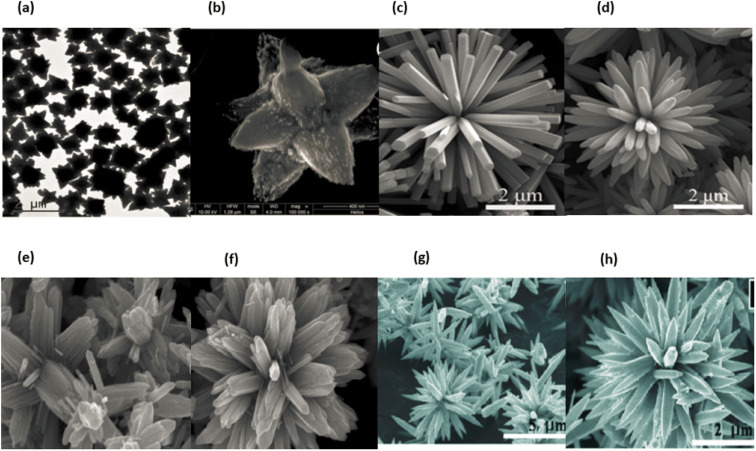
Typical SEM images of ZnO nanostars and nanoflowers (a) and (b) ref. 240, (c) and (d) ref. 241, (e) and (f) ref. 151, (g) and (h) ref. 242.

It is explained how each of the thiourea-infused growth treatments works. The entire sample occupied a space of 30 mL. In order to characterise each of the products, we utilized SEM (FEI XL 30), PL (Kratos Analytical Axis Ultra), and XPS (Kratos Analytical Axis Ultra). Measurements of photoluminescence (PL) were carried out at room temperature using a HORIBA Jobin Yvon LabRAM ARAMIS grating spectrometer in conjunction with the 325 nm line of a HeCd laser.^239^Fig. 26a–h displaying the ZnO nanostars and nanoflowers.

### 4.4. Fabrication technique

Many synthetic approaches were utilized to fabricate zinc oxide nanoparticles. They are primarily classified into three categories: chemical fabrication, physical fabrication, and biological fabrication.

#### 4.4.1. Chemical fabrication

The process of transforming the basic materials or reactant into a product through the utilization of one or more chemical processes is referred to as chemical fabrication. The process of chemical fabrication can be divided into two distinct phases: the gas phase and the liquid phase. The liquid phase can be further subdivided into precipitation/co-precipitation technique, colloidal technique, sol–gel technique, oil microemulsion technique, hydrothermal technique, and solvothermal technique, while the gas phase can be further separated into pyrolysis and gas condensation techniques.^243^

##### 4.4.1.1. Co-precipitation technique/precipitation technique

**Fig. 27:**
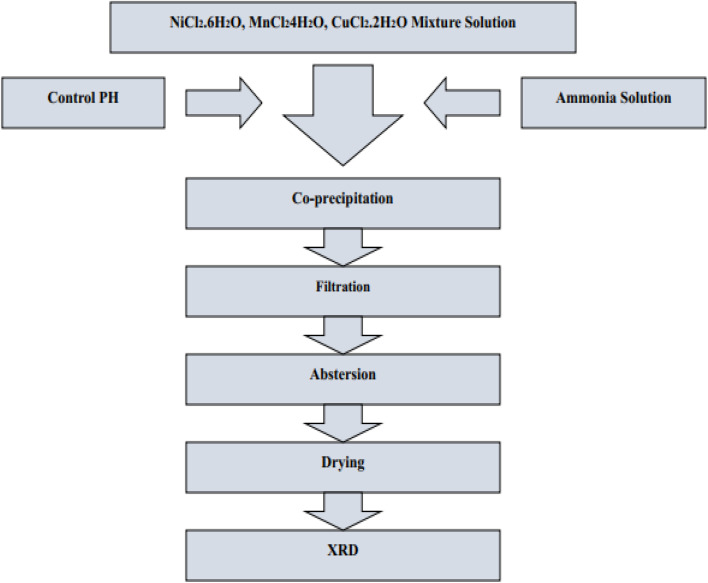
Flow chart of co-precipitation method.

In order to convert a solution into a solid using this method, either an insoluble form or a higher saturation level must be utilized. The treatment of zinc compounds begins with dilute hydrochloric acid, followed by dilute hydrochloric acid. The reaction is carried out at room temperature with gentle stirring, and a solution containing NaOH, KOH, and NH_4_OH is added drop by drop to act as a precursor. When the pH reaches a range between 8 and 10, the base solution addition process is stopped. The mixture described above is heated to 85 °C for six hours, then centrifuged, brought down to room temperature, and filtered. The white powder is formed as a result of precipitating the substance with distilled water in order to remove any impurities.^244–246^Fig. 27 displaying the flow chart of co-precipitation method.

##### 4.4.1.2. Sol–gel technique

**Fig. 28:**
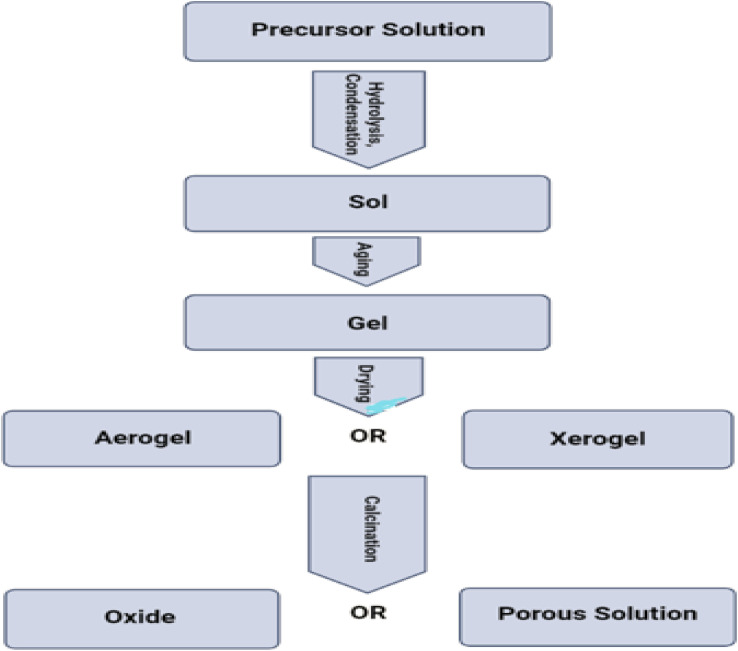
Flow chart sol–gel method.

The sol–gel method is a wet chemical procedure that can be used to produce a three-dimensional network. This approach is also known as the way of producing sol–gel materials. This process begins with the formation of a colloidal suspension, which is referred to as a sol, and is then followed by the gelation of the sol in a constant liquid phase, which is referred to as a gel. In this phase, the zinc compound is heated to 50 °C while being dissolved in double-distilled water. A magnetic stirrer is used throughout the process of gradually adding alcohol at a concentration of 100%, which is then followed by the dropwise addition of hydrogen peroxide until the solution becomes transparent. The solution was left to ferment for twenty-four hours before being dried at eighty degrees Celsius for a number of hours in order to generate white zinc oxide nanoparticles. In order to get rid of any traces of by products, wash many times in water that has been through two distillation processes, and then dry in an oven heated to 80 °C. During the drying process, zinc oxide is completely converted.^247^Fig. 28 displaying the flow chart of sol gel method.

##### 4.4.1.3. Microemulsion technique

**Fig. 29:**
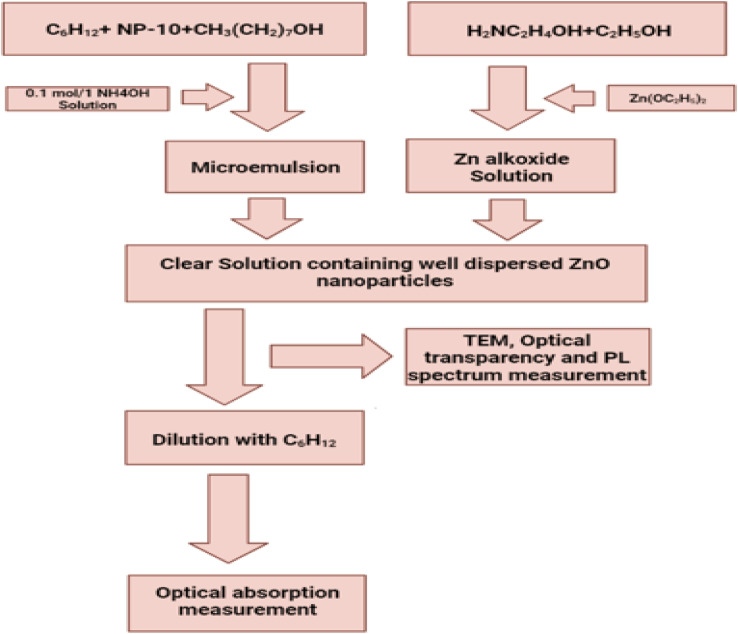
Flow chart ZnO microemulsion method.

Microemulsion is a liquid solution that is optically isotropic, thermodynamically stable, and is made up of water, oil, and amphiphile. In this particular investigation, zinc oxide nanoparticles were produced by a process known as reverse microemulsion. The substances *n*-heptane, glycerol and dioctyl sulfosuccinate sodium in that order, are utilised for the roles of surfactant, polar phase, and non-polar phase, respectively. The synthesis results in two different microemulsions, each of which has a different ratio of surfactants. Dissolving dioctyl sulfosuccinate sodium in *n*-heptane at room temperature while stirring continuously will result in the production of a microemulsion. After the ingredients have been combined, the solution should be cut into two equal parts and labelled solution A and solution B. The zinc compound is stirred into solution A while constantly being stirred while the other half of the glycerol is dissolved in the zinc compound. In the same manner, add some sodium hydroxide (NaOH) that has been dissolved in glycerol to solution B. The aforementioned two solutions were combined in a continuous mixing process at room temperature until they produced a solution that was clear. After that, gradually blend solution B with solution A while stirring constantly for twenty-four hours at a temperature between sixty and seventy degrees Celsius. Centrifuge the mixture for twenty minutes at a speed of 10 000 rpm to obtain a white solid powder. After being washed in a mixture of methanol and chloroform and centrifuged for ten minutes at ten thousand revolutions per minute, the product was dried for one hour at one hundred degrees Celsius in an open-air drying oven and then placed overnight in a vacuum drier at room temperature. This process took a total of twenty-four hours. Calcinated in an air atmosphere for three hours at temperatures ranging from 300 to 500 °C.^248,249^Fig. 29 displaying the flow chart of microemulsion method.

##### 4.4.1.4. Hydrothermal technique

**Fig. 30:**
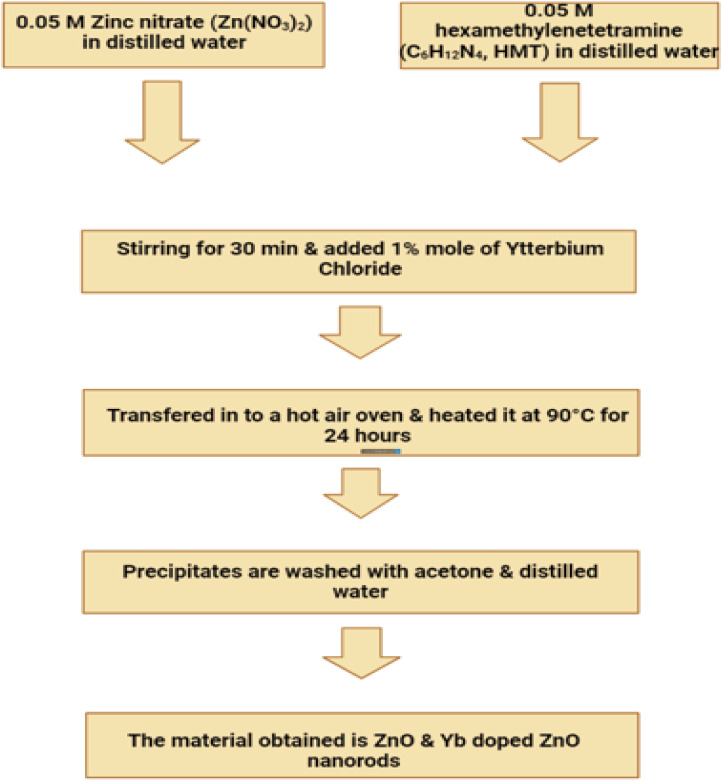
Flow chart ZnO hydrothermal method.

It is a method for the creation of single crystals that is predicated on the solubility of minerals in hot water that is subjected to intense pressure. To prepare stock solutions, zinc component is first stirred into methanol, then dissolved in the solvent. To modify the pH to a range between 8 and 11, NaOH that has been dissolved in methanol is added to the stock solution while it is being stirred continuously. After that, the solution was autoclaved in stainless steel autoclaves lined with Teflon for 6 and 12 h at temperatures ranging from 100 to 200 °C under autogenous pressure before being allowed to naturally cool down to ambient temperature. Following the completion of the reaction, the white solid product was extracted by washing it with methanol, filtering it, and then drying it in a laboratory oven at 60 °C.^250,251^Fig. 30 displaying the flow chart of hydrothermal method.

##### 4.4.1.5. Solvothermal technique

**Fig. 31:**
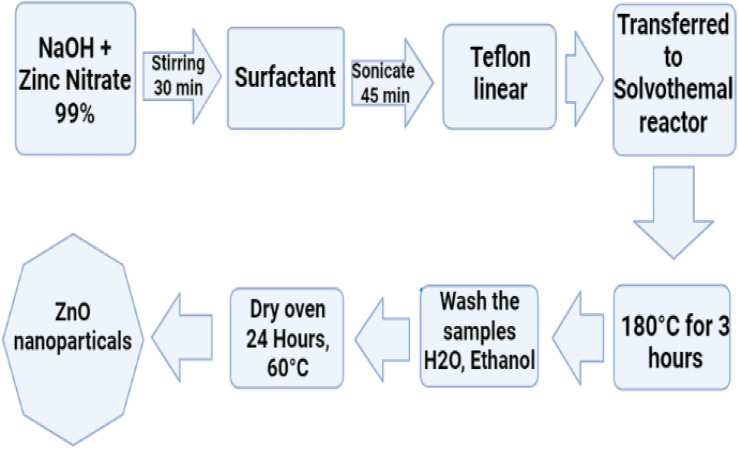
Flow chart ZnO hydrothermal method.

It is a method in which the solvent is added at a pressure and temperature ranging from moderate to high, which makes it easier for the precursors to interact with one another throughout the synthesis. In this particular experiment, ethylene glycol and ethanol were mixed together and used in the capacity of a solvent. For a period of twenty minutes, the zinc component should be mixed into the solvent solution. In order to reach the required temperature, the sealed chamber is kept inside a box furnace that has been preheated for a period of twelve hours. The experiment was carried out at a variety of temperatures, including 200 °C, 150 °C, and 135 °C, in order to calibrate the size of the nanoparticles. After that, the precipitate was collected, after that it was washed three times with ethanol and water, and finally it was dried in the air at room temperature.^252^Fig. 31 displaying the flow chart of solvothermal method.

##### 4.4.1.6. Pyrolysis technique

**Fig. 32:**
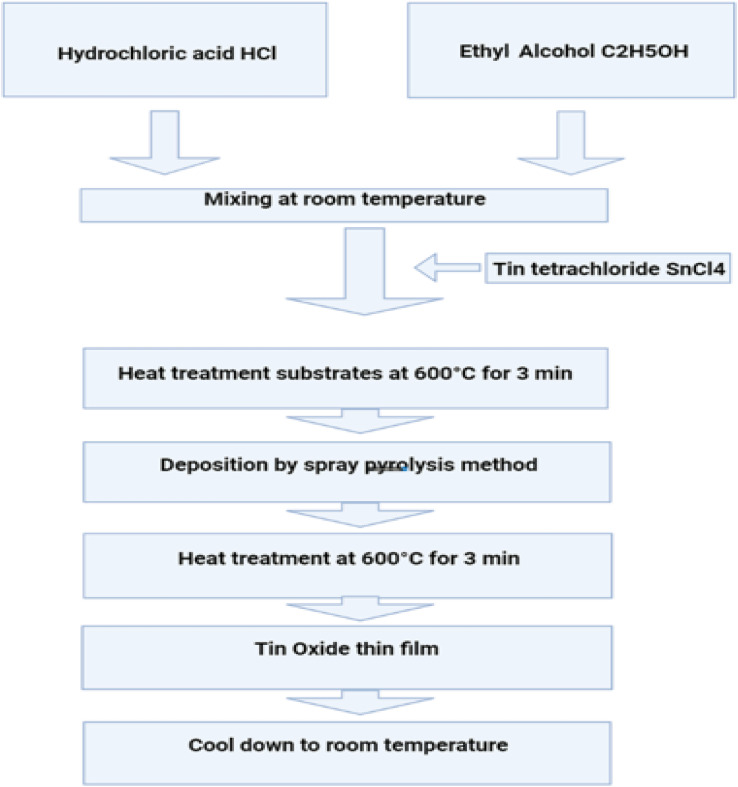
Flow chart SnO pyrolysis method.

The process known as pyrolysis begins with the atomization of a precursor solution, continues with the solution's evaporation, and concludes with the solution's decomposition into films and particles. In order to produce the precursor solution, the zinc component is first dissolved in the distillate water. Nebulization occurs in response to the pressure exerted by the surrounding air. In a reactor maintained at a temperature of 1200 °C, the droplets disintegrate. A cold precipitator is used to create nanoparticles, which are subsequently collected and dried in an oven at a temperature of 100 °C. Washing the product in water helped get rid of any unreacted zinc compound that was present in it.^253,254^Fig. 32 displaying the flow chart of pyrolysis method.

##### 4.4.1.7. Gas condensation technique

**Fig. 33:**
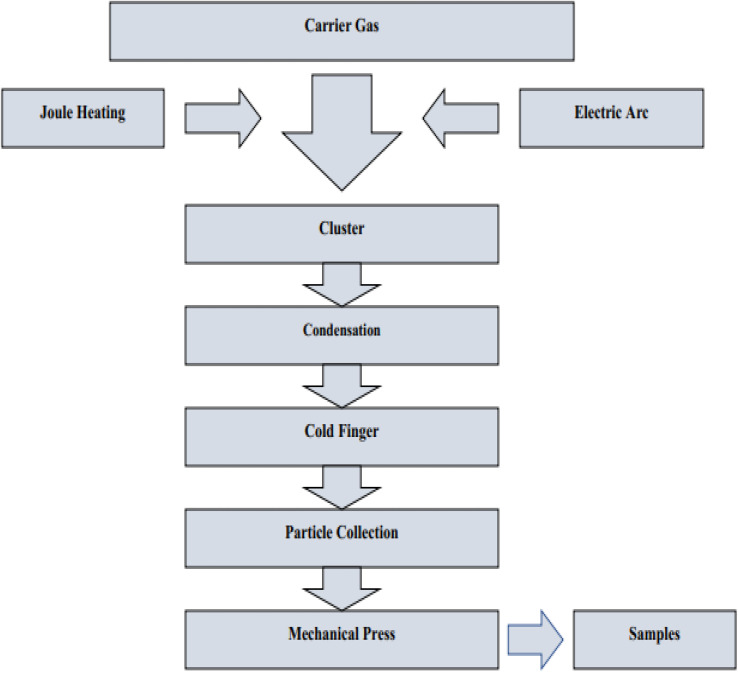
Flow chart gas condensation method.

Zinc compound is introduced into a chamber that is under vacuum. By utilising induced current and keeping the vacuum pressure and vaporisation temperature constant, the substance is melted and then evaporated into gas before being vaporised. An inert gas and material vapour have a collision inside of a vacuum chamber. After that, it travels to a collecting surface that is cooled to a low temperature, where it produces nanoparticles as it settles. We are able to simply manage the pressure and temperature by maintaining optimal conditions within the vacuum chamber. This is possible due to the fact that the temperature of the collection surface rises when liquid nitrogen flows continuously through the collector while it is located inside the vacuum chamber. Nanoscale production of metal nanoparticles begins with nucleation of the particles. The nanoparticles are amassed on the surface of the collector by the processes of vaporisation and condensation.^255^Fig. 33 displaying the flow chart of gas condensation method.

#### 4.4.2. Physical fabrication

Evaporating the material is the first stage in this bottom-up technique to the synthesis of nanostructural materials. The second step, rapid controlled condensation, is used to acquire particle size and is the second step. The three processes that fall under the category of physical synthesis are high-energy ball milling; solid, chemical and physical vapour deposition and laser ablation.

##### 4.4.2.1. High energy ball milling technique

**Fig. 34:**
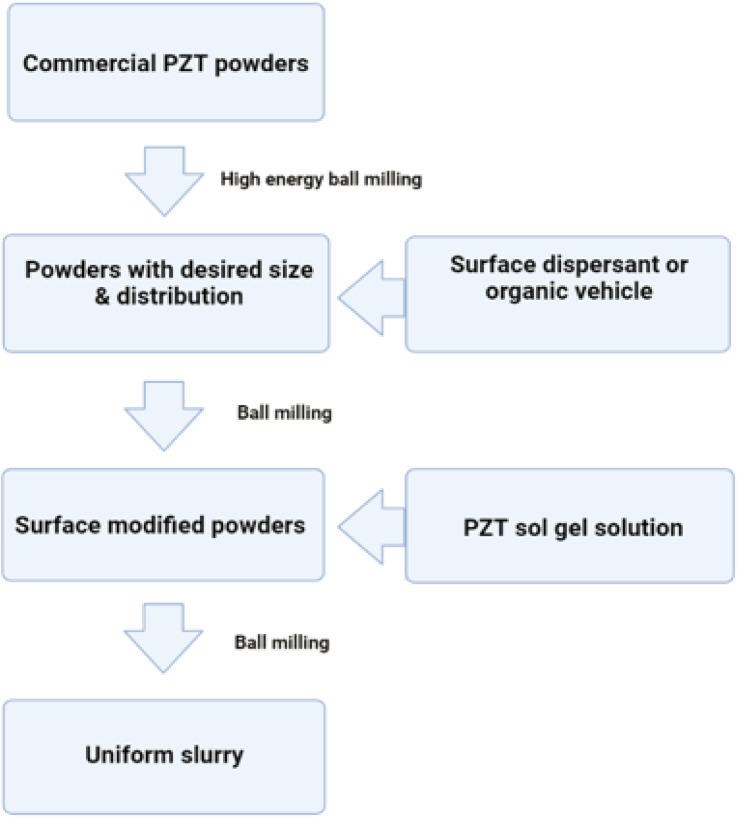
Flow chart high energy ball milling method.

The milling of ZnO powder takes anywhere from two hours to fifty hours, depending on the temperature and humidity of the surrounding air. Hardened steel balls are used in the milling process. In a horizontal oscillating mill, the milling process was carried out mechanically at a rate of 25 Hz. The ratio of zinc oxide powder to steel balls in the combination is 1 : 15, based on the weight of the individual components. The processing of the material was place without the use of any additional milling agents.^256^Fig. 34 displaying the flow chart of high energy ball milling method.

##### 4.4.2.2. Laser ablation

**Fig. 35:**
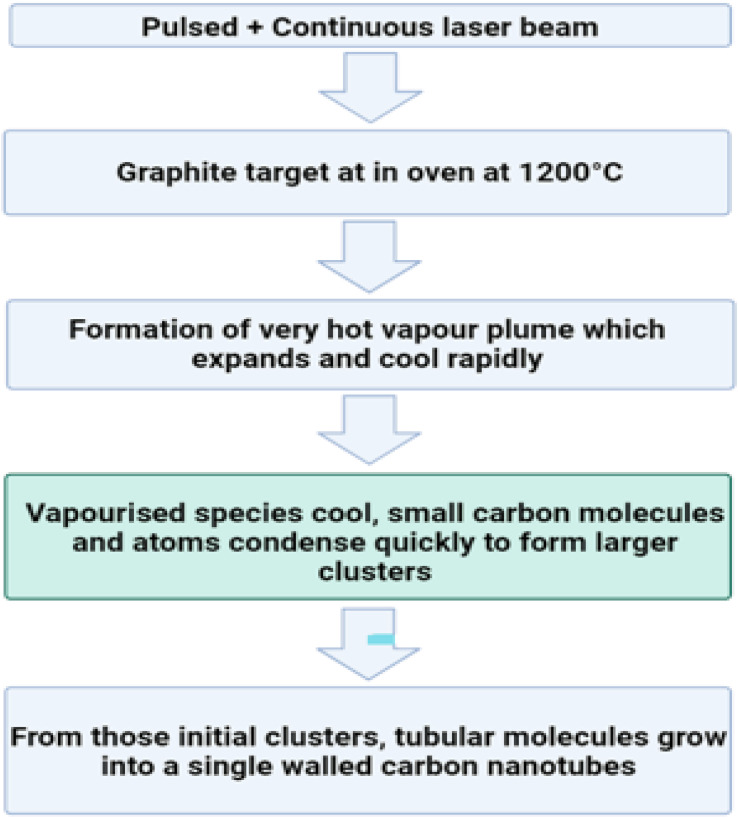
Flow chart carbon nanotubes laser ablation method.

First, prepare the solution by dissolving sodium dodecyl sulphate in double-distilled water. Next, irradiate a piece of zinc metal with Nd:YAG lasers at a frequency of 10 Hz for attentive output of secondary harmonics, with a focal length of 250 nm for 60 min and a total energy of 100 mJ. Nanoparticles of zinc were synthesized.^257^Fig. 35 displaying the flow chart of laser ablation method.

#### 4.4.3. Biological fabrication

This method refers to bioremediation, in which biological processes are used to degrade and metabolise chemical substances, restoring environmental quality. Plant-mediated and microbe-mediated biological synthesis are the two types of biological synthesis.

##### 4.4.3.1. Plant mediated technique

**Fig. 36:**
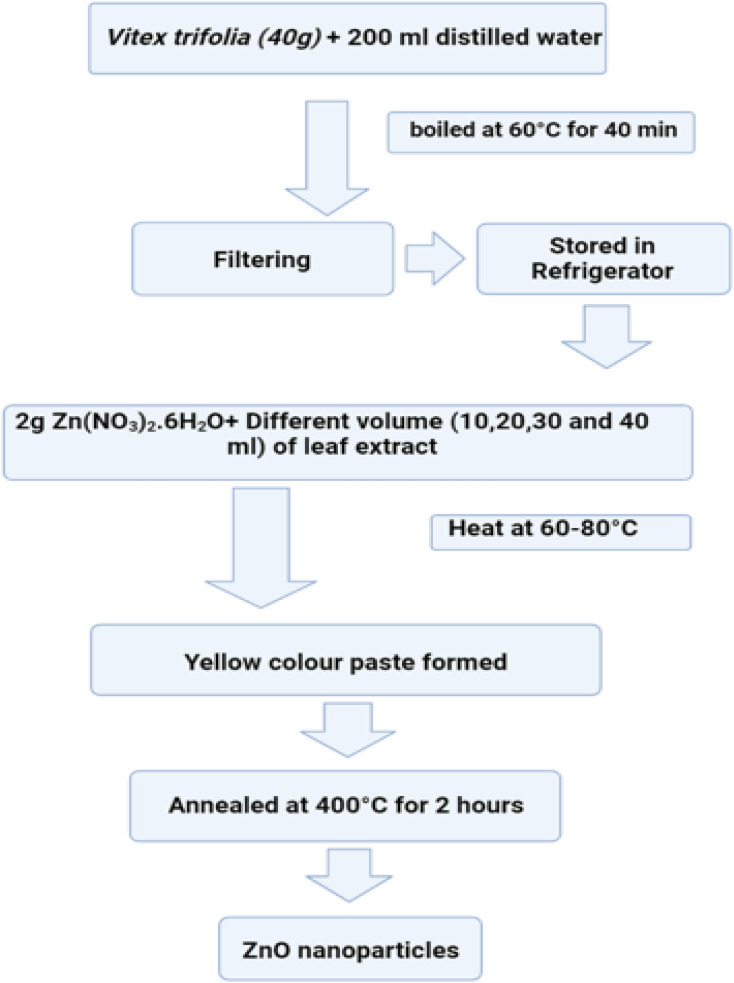
Flow chart ZnO plant mediated method.

In this process, nanoparticles are made by bioreducing metal ions to their most basic form utilising plants or plant components.^258^Fig. 36 displaying the flow chart of plant mediated method (Table 2).

**Table 2:** Synthesizing of different nanoparticles by using plants^259^

S. no	Plant name	Part of plant used	Type of NPs	Size	Applications
1	*Aloe vera*	Leaf	Gold and silver	10–30 nm	Optical coatings and cancer hyperthermia
2	*Syzygium aromaticum* (clove buds)	Leaf	Gold	5–10 nm	Detection and destruction of cancer cells
3	*Trifolium pratense*	Flower	ZnO	100–190 nm	Antimicrobial, antioxidant
4	*Annona squamosa*	Peel	TiO_2_	23 nm	Detection and destruction of cancer cells
5	*Citrus limon* (lemon)	Lemon extract	Silver	<50	Detection and destruction of cancer cells
6	*Azadirachta indica*	Leaves	ZnO	40 nm	Antimicrobial, antioxidant
7	*Aloe vera*	Leaf	TiO_2_	60 nm	Detection and destruction of cancer cells
8	*Albizia lebbeck*	Stem barn	ZnO	66.25 nm	Antimicrobial, antioxidant
9	*Psidium guajava*	Leaf	TiO_2_	32.58 nm	Detection and destruction of cancer cells
10	*Tinospora cordifolia*	Leaves	Cu	50–130 nm	Catalytic degradation

##### 4.4.3.2. Microbes mediated technique

**Fig. 37:**
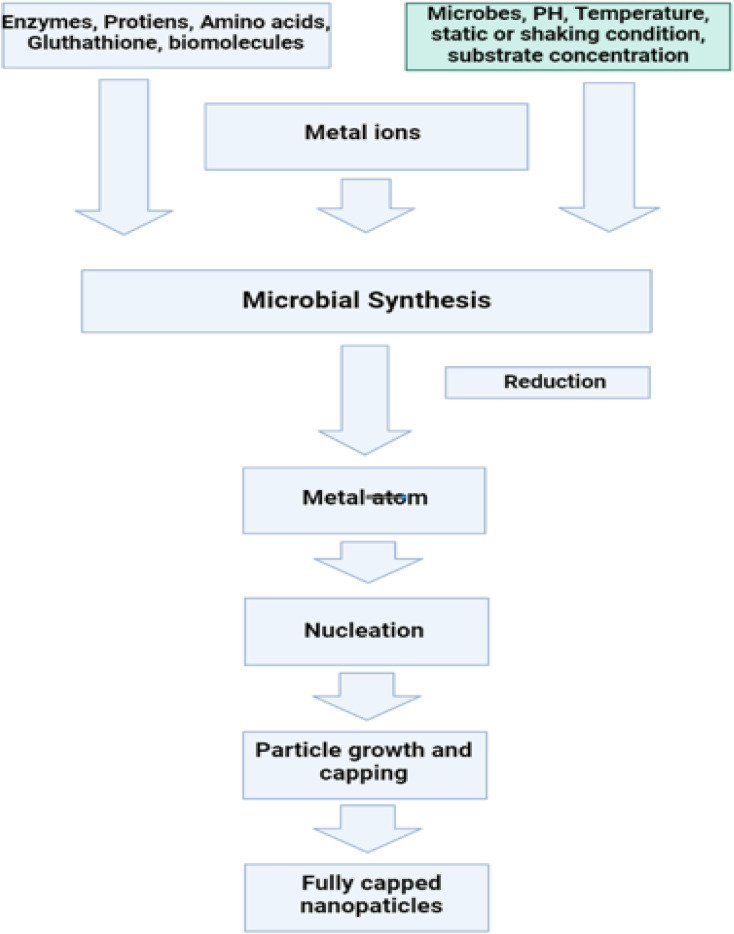
Flow chart of microbes mediated method.

The autoclave is used to make and sterilise the nutrition broth. Then, under aseptic conditions, bacterial strains are introduced, and the temperature is maintained overnight. The appearance of turbidity confirms bacterial growth, after which the supernatant and pellet were separated and studied under FTIR, XRD, UV spectrophotometer, and SEM.^260^Fig. 37 displaying the flow chart of microbes mediated method.

### 4.5. Application of ZnO

**Fig. 38:**
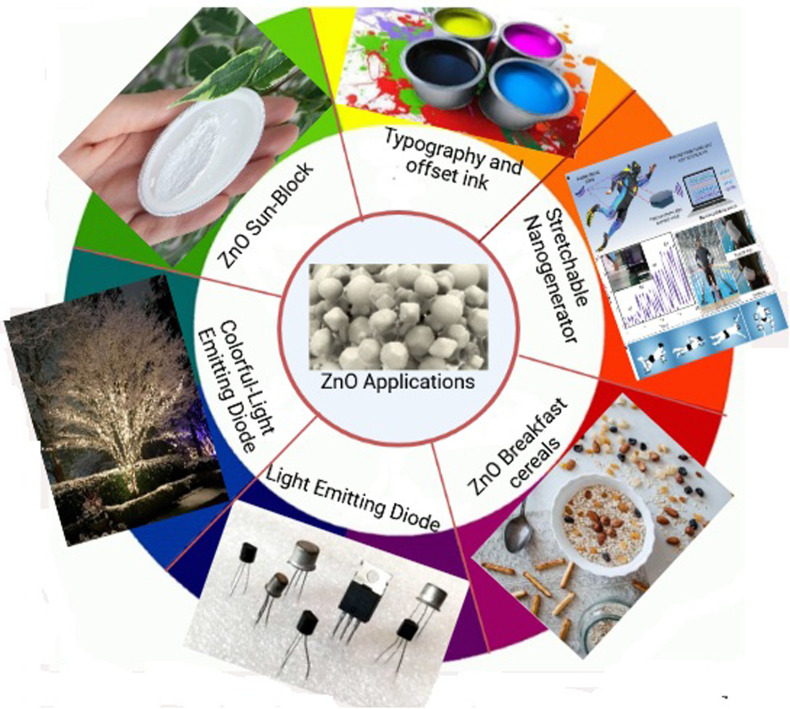
Schematic illustration of applications of the ZnO discussed below.

Zinc oxide's vast range of useful chemical and physical properties have led to its application in a diverse range of industries. It has many different applications, ranging from ceramics to tyres, agriculture to pharmaceuticals, and chemicals to paints. It is also employed in a wide number of different industries (Fig. 38).

(1) The global production of zinc oxide is approximately 10^5^ tonnes per year, with the rubber sector consuming the majority of it for the creation of various cross-linked rubber products.^261^ It is possible to increase the heat conductivity of traditional pure silicone rubber by adding thermal conductivity fillers such as metal oxides, metal powders, and inorganic particles. Although traditional pure silicone rubber has a poor heat conductivity, this property can be improved. It is possible for certain types of thermal conductivity powders, such as AlN_3_, MgO, Al_2_O_3_, ZnO, and SiO_2_, to increase the thermal conductivity of silicone rubber while maintaining its high electrical resistance. Because of this, these powders are attractive options for high-performance engineering materials. It is feasible to achieve high thermal conductivity even at a low filler content by using nanoscale fillers, which are used in the manufacturing process. However, as a result of the weak connection that exists between the surface of the nanoparticles and the polymer matrix, the ZnO nanoparticles have a tendency to group together in the polymer matrix to form larger particles. Techniques that modify the surface are utilised in order to improve the interaction of nanoparticles with the polymer in order to solve this problem. Das *et al.*^262^ demonstrate how, during the curing process, a hydrosilylation procedure is used to incorporate unmodified and surface-modified ZnO nanoparticles into the silicone rubber. Both of these types of nanoparticles include the vinyl silane group. Both the silicone rubber/zinc oxide (SR/ZnO) and the silicone rubber/silicon dioxide at zinc oxide (SR/SiVi@ZnO) nanocomposites were investigated in terms of their related structure, morphology, and properties. Yuan *et al.* employed a sol–gel approach to generate zinc oxide nanoparticles (with an average size of less than 10 nm). After that step was completed, the silicone coupling agent VTES was successfully incorporated onto the surface of the nanoparticles. Both the thermal conductivity and the mechanical properties of the SR/SiVi@ZnO nanocomposites were significantly enhanced as a result of the formation of a cross-linking structure within the silicone rubber matrix as well as an improvement in the dispersion of the nanocomposites within that matrix.

(2) Zinc oxide is an extremely efficient and often used crosslinker for carboxylated elastomers.^263,264^ It is possible to produce vulcanizates that have a high tensile strength, resistance to tearing, and level of hardness as well as hysteresis. The increased mechanical capabilities of ionic elastomers are primarily the result of their high stress relaxation capacity. This is because the enhanced mechanical capabilities of ionic elastomers are caused by the slippage of elastomer chain molecules on the surface of ionic clusters and the reformation of ionic bonds when the sample is deformed externally. In addition, ionic elastomers have thermoplastic properties, and when they are in a molten state, they can be handled just like any other thermoplastic polymer.^265^ However, zinc oxide-crosslinked carboxylic elastomers have a few drawbacks that need to be considered. Their scorchiness, complete absence of flex, and high compression set are the features that stand out the most. In order to prevent searchability, carboxylated nitrile elastomers are cross-linked utilising systems consisting of zinc peroxide or zinc peroxide/zinc oxide. Although the development of ionic crosslinks accounts for the vast majority of the vulcanization of XNBR with zinc peroxide, the action of the peroxide also results in the formation of covalent connections between the individual chains of the elastomer. However, longer vulcanization durations are required in order to achieve vulcanizates with equivalent tensile strength and crosslink density to zinc oxide-crosslinked vulcanizates. This is because zinc oxide is a crosslinking agent. In the case of XNBR vulcanization utilising zinc peroxide/zinc oxide systems, the curing process is comprised of at least three stages: the rapid synthesis of ionic crosslinks as a result of the initial zinc oxide present; peroxide crosslinking resulting in the development of covalent bonds (peroxide action); and ionic crosslinking as a result of the production of zinc oxide as a result of the peroxide decomposing. The third stage, which takes place gradually over long periods of time and results in degradation, very certainly involves the production of ionic species in some way. The vulcanization periods that can be achieved with XNBR that has been crosslinked with zinc oxide are substantially longer than those that can be achieved with XNBR that has been vulcanised using any other method. Despite the fact that it can cause burns in some situations, zinc oxide is nevertheless extensively utilised as a cross-linking agent in carboxylated nitrile rubbers. This is the case despite the fact that zinc oxide can induce burns. When it comes to the process of cross-linking, the parameters that are most important in determining its activity are the particle size, surface area, and shape of the zinc oxide. This is due to the fact that zinc oxide reacts with elastomer carboxylic groups to form carboxylic salts, which are also known as ionic crosslinks. The nature of the interphase that develops between the cross-linking agent and the elastomer chains is determined by these factors.^266^

(3) Hamed *et al.*^267^ used a solid-state pyrolytic process to synthesize nano zinc oxide. Studies on surface area and microscopic pictures showed that the generated zinc oxide had a surface area that ranged from 12 to 30 m^2^ g^−1^ and had particle sizes that ranged from 15 to 30 nm. The particle sizes ranged from 15 to 30 nm. The neoprene rubber that the researchers employed had a trace amount of zinc oxide, which served as a curing agent and was incorporated into the substance. It was demonstrated that a modest dosage of ZnO was optimal, particularly in comparison to the ZnO that is found in commercial items. Evaluations were conducted on the rubber's curing qualities as well as its mechanical properties, and the results were compared to those of conventionally cured rubbers made with zinc oxide. It was discovered that a low concentration of zinc oxide was sufficient to provide comparable curing and mechanical properties to neoprene rubber that utilised a higher concentration of commercial zinc oxide. This was discovered by comparing the properties of neoprene rubber that used a higher concentration of commercial zinc oxide. This was a finding that came about as a result of the observation that a low concentration of zinc oxide was sufficient. It all started with the observation that a small amount of zinc oxide was sufficient.

(4) Zinc oxide is commonly employed in the creation of several types of pharmaceuticals due to its drying, antibacterial, and disinfecting qualities.^268,269^ Historically, it was utilized in the treatment of epilepsy, and later on, it was utilized in the treatment of diarrhoea. At the moment, it is employed in the region, most frequently in the form of creams and ointments, but less frequently in the form of liquid powders and dusting powders. Dusting powders and liquid powders are utilized less frequently. Zinc oxide is frequently used in dermatological goods intended to treat inflammation and irritation because of its capacity to stimulate wound healing. These products can be found on the market. Peeling of the skin can occur when it is exposed to higher amounts of the substance. In addition to that, it is also available in suppositories. Additionally, it has use in the field of dentistry, primarily as a component of dental pastes and additionally for the production of temporary fillings. Zinc oxide is also utilized to give dietary zinc in a variety of dietary supplements and nutritional items. This is necessary because zinc is essential for human health.^270^

(5) Before the introduction of nanoparticles of TiO_2_ and ZnO, sun lotions had heavy formulations that did not rub well into the skin and were cosmetically unpleasant. Because of their ability to absorb UVA and UVB rays, these chemicals began to be used in creams. A new cream formula including a combination of TiO_2_ and ZnO solved the problem of an insufficiently white layer, resulting in a more clear, less sticky, and much simpler to rub into the skin medium.^271^ Titanium and zinc oxides have been demonstrated in a number of studies to be excellent sun cream media because they absorb UV rays, do not irritate the skin, and are quickly absorbed into the skin.^272–274^

(6) Zinc oxide is biocompatible for textile applications, and nanostructured zinc oxide coatings are more air-permeable and UV-blocking efficient than their bulk equivalents.^275^ As a result, ZnO nanostructures have attracted a lot of interest as UV-protective textile coatings. Using ZnO nanostructures, various ways for producing UV-protecting textiles have been reported. For example, UV-blocking capabilities of hydrothermally produced ZnO nanoparticles in SiO_2_-coated cotton fabric were outstanding.^276^ After synthesis in a homogeneous phase reaction at high temperatures, deposition of ZnO nanoparticles on wool and cotton fibres resulted in a significant increase in UV-absorbing activity.^277^ UV protection was equally impressive with zinc oxide nanorod arrays grown on a fibrous substrate utilising a low-temperature growing method.^278^

(7) Zinc oxide is a unique and crucial semiconductor with numerous electronic and electrotechnology applications.^279–281^ At ambient temperature, zinc oxide has a wide energy band of 3.37 eV and a high bond energy of 60 meV, making it useful in photoelectronics^282^ and electronic equipment,^283^ devices emitting a surface acoustic wave,^284^ field emitters,^285^ sensors,^286–289^ ultraviolet lasers,^290^ and solar cells.^291^ ZnO also shows luminescence (most notably photoluminescence—the production of light in the presence of electromagnetic radiation). It is employed in field emission display equipment, such as televisions, because to this property. It outperforms typical materials such as sulphur and phosphorus (phosphorescent compounds) in terms of UV resistance and electrical conductivity. The photoluminescent properties of zinc oxide are affected by the crystals size, the presence of defects in the crystalline structure, and the temperature.^292–295^ ZnO is a semiconductor, and thin films made of it have strong conductivity and visible light permeability. It can be used to make light-permeable electrodes for solar batteries because of these qualities. It's also a promising material for ultraviolet-emitting devices and could be used as a transparent electrode in photovoltaic and electroluminescent equipment.^296,297^

(8) The addition of zinc oxide cuts down on the amount of time needed for manufacture and boosts the resistance of concrete to the action of water. Additionally, the incorporation of zinc oxide into Portland cement causes a delay in the processes of hardening and quenching (*i.e.*, it causes a delay in the progressive generation of heat), and it also results in an improvement in the whiteness and final strength of the cement. Zinc silicates are compounds that are resistant to water and fire that is formed when zinc oxide is combined with silicates (for example, sodium silicate). These compounds are utilized as paint binders. These compounds, which are resistant to fire and act as adhesives, are put to use in the building industry to bind cements. A Cu/ZnO/Al_2_O_3_ catalyst is utilized in the production of methanol, which is the third most important product produced by the chemical industry. The active component of this catalyst is minute Cu particles, which are driven by their interaction with the zinc oxide substrate.^298^

(9) In addition, zinc oxide is utilized in the production of typographic as well as offset inks. It offers superior qualities as a printing medium (high fluidity). The utilization of zinc oxide helps to improve the covering power, pure shade, and longevity of the inks and it also prevents darkening from occurring. Zinc oxide is another ingredient that gives colours their shine.^299^

(10) It can be discovered in a wide variety of meals, such as breakfast cereals, among others. Zinc is utilized from zinc oxide as a source of zinc, which is an important nutrient. Due to the unique chemical and antifungal properties of ZnO and its derivatives, they are also utilized in the manufacturing and packing of animal products (such as fish and meat) and vegetable items. These applications include: (*e.g.*, peas and sweetcorn).^300^

(11) Fungi and moulds are hampered in their attempts to form and grow when exposed to ZnO or one of its derivatives. Zinc oxide is often used as a means of boosting the effectiveness of fungicides. As zinc oxide encourages healthy growth in animals, it is increasingly being used as an addition in animal feed. This trend is expected to continue. Additionally, it is utilized in the production of synthetic fertilizer.^301^

(12) In addition, zinc oxide can be utilized in the field of criminology, particularly in the process of mechanical fingerprint analysis. It is also included in cigarette filters because of its ability to selectively remove certain components of the smoke produced by tobacco products. To eliminate substantial quantities of H_2_S and HCN from tobacco smoke in a manner that does not result in the production of an offensive odour, filters are made of charcoal that has been loaded with zinc oxide and iron oxide. In addition to this, it is capable of extracting sulphur and compounds of sulphur from a wide variety of gases and liquids, most notably waste gases from industrial processes. In addition to this, zinc is capable of removing H_2_S from hydrocarbon gases and desulfurizing H_2_S together with other forms of sulphur.^302^

(13) In addition, zinc oxide and its derivatives find use in the automotive industry as a lubricant additive, where they help reduce fuel consumption and protect against oxygen corrosion. In addition, zinc oxide has been utilized in a range of lubricants, including solid lubricants, vibration-resistant lubricants, and EP additives, amongst other applications. In the not-too-distant future, the adhesive properties of ZnO could perhaps be utilized.^303^

(14) Arnold *et al.*^303^ used individual nanobelts to create field effect transistors (FETs). Using ultrasonication, large packets of zinc oxide nanobelts were diffused in ethanol until the majority of the nanobelts were separated. This process was repeated several times. In order to conduct an atomic force microscopy investigation, these distributed nanobelts were heated on a substrate made of SiO_2_/Si. Depositing zinc oxide nanobelt diffusions on SiO_2_/Si(p+) substrates and then heating them in an oxygen atmosphere at 800 °C for two hours was the process that was used to produce ZnO field effect transistors (FETs). These substrates were first spun coated with a polymer called poly methyl meth acrylate (PMMA), then baked, and finally exposed to electron-beam lithography so that electrode arrays could be developed and characterised. The leftover PMMA was removed using hot acetone, and then a 30 nm titanium layer was formed using electron-beam evaporation to act as the source and drain for these electrodes. By altering the gate voltage, this FET device would be able to control the amount of current that was flowing from the source to the drain.

(15) ZnO nanoparticles have excellent luminous characteristics. Costenaro *et al.*^304^ used co-precipitation method to create ZnO nanoparticles with varying amounts of aminopropyltriethoxy silane (APTS). They created LED devices using these ZnO nanoparticles and reported improved luminous characteristics. Liu *et al.*^305^ used a carbon cloth template hydrothermal technique to create a photodetector using flexible nanoparticle-assembled ZnO cloth. Under UV irradiation, more than 600 separate measurements of the device's conductance were taken, and the response and decay periods came out to approximately 3.2 and 2.8 s, respectively.

(16) Bagabas *et al.*^306^ advised ZnO nanoparticles for environmental applications. They spotted the photodegradation of cyanide ions while creating ZnO nanoparticles using cyclohexylamine in aqueous and ethanolic media. They found that the structure was essential to boosting the photocatalytic breakdown proficiency of cyanide ion, and they discovered this. Hong *et al.*^307^ used a precipitation process to create ZnO nanoparticles, which had a high photocatalytic activity.

(17) Because of their exceptional biocompatibility, ZnO nanoparticles have the potential to be utilized in various applications, including medication delivery and bioimaging^308^ and low cost.^309^ Nanoparticles with magnetic and luminous properties have potential as drug carriers and detecting probes, among other things.

(18) Matsuyama *et al.*^310^ suggested biomedical uses of silica-layered zinc oxide quantum dots with biotin as fluorophore for cell-labeling applications, as well as cautious destruction in malignant cell applications.^311^

(19) Zinc oxide nanomaterials have shown that they can be used to detect DSSC substances^312^ and are used as UV blockers in sunscreen lotion.^313^ Based on their drug transport, anticancer, bioimaging activity, antibacterial, and anti-inflammatory properties, ZnO nanoparticles have demonstrated potential for usage in a variety of biomedical applications.^314^

(20) Sun *et al.*^315^ proposed that ZnO nanostructures have the potential to be utilized in applications involving the collection of energy as a stretchable nanogenerator.

## Summary

The chemistry of nanomaterials (NMs) and nanoparticles (NPs) are a burgeoning field of research and a rapidly expanding technological sector in a wide variety of application domains. Nanoparticles are separated into their respective categories based on their morphology, which refers to their structure, as well as their size and shape. One-dimensional nanomaterials, two-dimensional nanomaterials, and three-dimensional nanomaterials are the three types of nanomaterials on the basis of their dimension. The most important innovations of the 21st century are the design and fabrication of nanoscale materials made of metal oxides, metals, carbon allotropes and chalcogenides. These materials are used in a vast range of fields, such as energy storage, catalysis and biosensors, conversion devices and biomedical applications. In particular, the unique physiochemical properties of semiconducting metal oxides, such as SnO_2_, ZnO, and TiO_2_, which vary depending on size and shape, have been extensively researched and exploited. One of the most stable n-type semiconducting materials for chemical and thermal applications is ZnO, which is available in a variety of forms including pellets, bulk crystal and thin film for use in everything from luminescent materials to batteries, supercapacitors and solar cells to biomedical and photocatalysis sensors. The variation of ZnO nanostructures is determined by the growth mechanism, the growth method, the synthesis conditions, and the type of substrate. Nanowires, Nanorods, nanotubes, nanocolumns, nanorings, nanobelts, nanosheet networks, nanoribbons, nanoflowers, hollow micro- and nanospheres and nanocombs are among the nanostructures. These nanostructures can be fabricated quite easily at very low temperature, and a variety of different growth techniques for ZnO nanostructures have been documented, including chemical, physical and biological techniques. The process of chemical fabrication can be divided into two distinct phases: the gas phase and the liquid phase. The liquid phase can be further subdivided into precipitation/co-precipitation technique, colloidal technique, sol–gel technique, oil microemulsion technique, hydrothermal technique, and solvothermal technique, while the gas phase can be further separated into pyrolysis and gas condensation techniques (CVD) and thermal evaporation. The three processes that fall under the category of physical synthesis are high-energy ball milling, solid, chemical and physical vapour deposition and laser ablation. Plant-mediated and microbe-mediated biological synthesis are the two types of biological synthesis. Because of the growth procedures, disciplines and applications that were discussed above, ZnO has the potential to become one of the most significant candidates for use in future research and applications. Zinc oxide's vast range of useful chemical and physical properties have led to its application in a diverse range of industries. It has many different applications, ranging from ceramics to tyres, agriculture to pharmaceuticals, and chemicals to paints. It is also employed in a wide number of different industries.

## Conflicts of interest

The authors declared no potential conflicts of interest.

## Supplementary Material

## References

[cit1] Khan R., Shigidi I., Otaibi S. A., Althubeiti K., Abdullaev S. S., Rahman N., sohail M., Khan A., Iqbal S., Del Rosso T., Zaman Q., Khan A. (2022). Room temperature dilute magnetic semiconductor response in (Gd, Co) co-doped ZnO for efficient spintronics applications. RSC Adv..

[cit2] United Nations , Questions About Nanotechnology, 2012, https://www.epa.gov/chemical-research/research-nanomaterials, accessed Aug 21, 2014

[cit3] Considering Whether an FDA-Regulated Product Involves the Application of Nanotechnology, Federal Drug Administration, USA, 2011, https://www.fda.gov/RegulatoryInformation/Guidances/ucm257698.html, accessed Jan 25, 2016

[cit4] ISO/TS 80004-1:2010 , Nanotechnology – Vocabulary – Part 1: Core Terms, International Organization for Standardization, Geneva, Switzerland, 2010, https://www.iso.org/standard/51240.html, accessed July 17, 2017

[cit5] Bleeker E. A. J. , Cassee F. R., Geertsma R. E., de Jong W. H., Heugens E. H. W., Koers-Jacquemijns M., van De Meent D., Oomen A. G., Popma J., Rietveld A. G. and Wijnhoven S. W. P., Interpretation and implications of the European Commission's definition on nanomaterials; Letter report 601358001, RIVM, Bilthoven, Netherlands, 2012

[cit6] Potocnik J. (2011). Off. J. Eur. Communities: Legis..

[cit7] Feynman R. P. , There's Plenty of Room at the Bottom, Engineering and Science Magazine, California Institute of Technology, 1960

[cit8] Laurent S. (2008). *et al.*, Magnetic iron oxide nanoparticles: Synthesis, stabilization, vectorization, physicochemical characterizations and biological applications. Chem. Rev..

[cit9] Tiwari J. N., Tiwari R. N., Kim K. S. (2012). Zero-dimensional, One dimensional, Two-dimensional and Three-dimensional Nanostructured Materials for Advanced Electrochemical Energy Devices. Prog. Mater. Sci..

[cit10] Tiwari D. K. , Behari J. and Sen P., Application of Nanoparticles in Waste Water Treatment, 2008, vol. 1

[cit11] Mansha M. (2017). *et al.*, Synthesis, Characterization and VisibleLight-Driven Photoelectrochemical Hydrogen Evolution Reaction of Carbazole-Containing Conjugated Polymers. Int. J. Hydrogen Energy.

[cit12] Rao J. P., Geckeler K. E. (2011). Polymer Nanoparticles: Preparation Techniques and Size-Control Parameters. Prog. Polym. Sci..

[cit13] Pantic I. (2010). Magnetic nanoparticles in cancer diagnosis and treatment: novel approaches. Rev. Adv. Mater. Sci..

[cit14] Valko M., Morris H., Cronin M. (2005). Metals, Toxicity and Oxidative Stress. Curr. Med. Chem..

[cit15] Kolosnjaj-Tabi J. (2015). *et al.*, The One Year Fate of Iron Oxide Coated Gold Nanoparticles in Mice. ACS Nano.

[cit16] Berry C. C. (2005). Possible exploitation of magnetic nanoparticle–cell interaction for biomedical applications. J. Mater. Chem..

[cit17] Dreaden E. C., Alkilany A. M., Huang X., Murphy C. J., El-Sayed M. A. (2012). The golden age: Gold nanoparticles for biomedicine. Chem. Soc. Rev..

[cit18] Salavati-Niasari M., Davar F., Mir N. (2008). Synthesis and Characterization of Metallic Copper Nanoparticles *via* Thermal Decomposition. Polyhedron.

[cit19] Faraday Michael (1857). The Bakerian Lecture. —Experimental relations of gold (and other metals) to light. Philos. Trans. R. Soc. London.

[cit20] Kumar H., Venkatesh N., Bhowmik H., Kuila A. (2018). Metallic nanoparticle: a review. Biomed. J. Sci. Technol. Res..

[cit21] Fan G., Dundas C. M., Zhang C., Lynd N. A., Keitz B. K. (2018). Sequence-Dependent Peptide Surface Functionalization of Metal-Organic Frameworks. ACS Appl. Mater. Interfaces.

[cit22] Tai C. Y., te Tai C., Chang M. H., Liu H. S. (2007). Synthesis of magnesium hydroxide and oxide nanoparticles using a spinning disk reactor. Ind. Eng. Chem. Res..

[cit23] Sathyanarayanan M. B., Balachandranath R., Genji Srinivasulu Y., Kannaiyan S. K., Subbiahdoss G. (2013). The Effect of Gold and Iron-Oxide Nanoparticles on Biofilm-Forming Pathogens. ISRN Microbiol..

[cit24] Salas P., Odzak N., Echegoyen Y., Kägi R., Sancho M. C., Navarro E. (2019). The role of size and protein shells in the toxicity to algal photosynthesis induced by ionic silver delivered from silver nanoparticles. Sci. Total Environ..

[cit25] Prasanna S. S. , Balaji K., Pandey S., and Rana S., Metal oxide-based nanomaterials and their polymer nanocomposites, in Nanomaterials and Polymer Nanocomposites, ed N. Karak, Elsevier Inc., Amsterdam, 2019, pp. 123–144

[cit26] Azam A., Ahmed A. S., Oves M., Khan M. S., Habib S. S., Memic A. (2012). Antimicrobial activity of metal oxide nanoparticles against Gram-positive and Gram-negative bacteria: A comparative study. Int. J. Nanomed..

[cit27] Sigmund W. (2006). *et al.*, Processing and structure relationships in electrospinning of ceramic fiber systems. J. Am. Ceram. Soc..

[cit28] Thomas S. C., Kumar Mishra P., Talegaonkar S. (2015). Ceramic Nanoparticles: Fabrication Methods and Applications in Drug Delivery. Curr. Pharm. Des..

[cit29] Fadeel B., Garćia-Bennett A. E. (2010). Better safe than sorry: understanding the toxicological properties of inorganic nanoparticles manufactured for biomedical applications. Adv. Drug Delivery Rev..

[cit30] Safeen A. (2022). Enhancing the physical properties and photocatalytic activity of TiO_2_ nanoparticles via cobalt doping. RSC Adv..

[cit31] Khan R., Tirth V., Ali A., Irshad K., Rahman N., Algahtani A., Sohail M., Isalm S. (2021). Effect of Sn-doping on the structural, optical, dielectric and magnetic properties of ZnO nanoparticles for spintronics applications. J. Mater. Sci.: Mater. Electron..

[cit32] Feng L. (2019). *et al.*, Synthesis and photoluminescence properties of silica-modified SiO2@ANA-Si-Tb@SiO2, SiO2@ANA-Si-Tb-L@SiO2 core–shell–shell nanostructured composites. R. Soc. Open Sci..

[cit33] Ijaz I., Gilani E., Nazir A., Bukhari A. (2020). Detail review on chemical, physical and green synthesis, classification, characterizations and applications of nanoparticles. Green Chem. Lett. Rev..

[cit34] Mostofizadeh A., Li Y., Song B., Huang Y. (2011). Synthesis, properties, and applications of low-dimensional carbon-related nanomaterials. J. Nanomater..

[cit35] Odom T. W., Huang J. L., Kim P., Lieber C. M. (1998). Atomic structure and electronic properties of single-walled carbon nanotubes. Nature.

[cit36] Zhang H., Grüner G., Zhao Y. (2013). Recent advancements of graphene in biomedicine. J. Mater. Chem. B.

[cit37] Yang K., Feng L., Shi X., Liu Z. (2013). Nano-graphene in biomedicine: Theranostic applications. Chem. Soc. Rev..

[cit38] Song L., Shi J., Lu J., Lu C. (2015). Structure observation of graphene quantum dots by single-layered formation in layered confinement space. Chem. Sci..

[cit39] Wang S., Cole I. S., Li Q. (2016). The toxicity of graphene quantum dots. RSC Adv..

[cit40] Li S. (2017). *et al.*, Exceptionally High Payload of the IR780 Iodide on Folic Acid-Functionalized Graphene Quantum Dots for Targeted Photothermal Therapy. ACS Appl. Mater. Interfaces.

[cit41] Chen F. (2017). *et al.*, Graphene quantum dots in biomedical applications: Recent advances and future challenges. Front. Med..

[cit42] Bhaviripudi S. (2007). *et al.*, CVD synthesis of single-walled carbon nanotubes from gold nanoparticle catalysts. J. Am. Chem. Soc..

[cit43] K. A.-J. of the C. Society , Isolation, separation and characterisation of the fullerenes C 60 and C 70: the third form of carbon, 1990, accessed: Feb. 04, 2022, https://pubs.rsc.org/en/content/articlehtml/1990/c3/c39900001423

[cit44] Kroto H. , Heath J., O'Brien S., Curl R. and R. S., nature, 1985, accessed: Feb. 04, 2022, https://www.nature.com/articles/318162a0

[cit45] Georgakilas V., Perman J. A., Tucek J., Zboril R. (2015). Broad Family of Carbon Nanoallotropes: Classification, Chemistry, and Applications of Fullerenes, Carbon Dots, Nanotubes, Graphene, Nanodiamonds, and Combined Superstructures. Chem. Rev..

[cit46] Yadav B. , R. K.-I. J., Structure, properties and applications of fullerenes, 2008, accessed: Feb. 04, 2022, https://www.academia.edu/download/44397281/Structure_properties_and_applications_of20160404-18129-nfful8.pdf

[cit47] M. D.-P. World , Carbon footballs bounce into the limelight, 1993, accessed: Feb. 04, 2022, https://iopscience.iop.org/article/10.1088/2058-7058/6/10/27/meta

[cit48] Matija L. , et al., *In vitro* and *in vivo* investigation of collagen-C60 (OH)24 interaction, Trans Tech Publ, 2004, accessed: Feb. 04, 2022, https://www.scientific.net/MSF.453-454.561.pdf

[cit49] Chen Z. , Ma L., Liu Y., Theranostics C. C., Applications of functionalized fullerenes in tumor theranostics, 2012, accessed: Feb. 04, 2022, https://www.ncbi.nlm.nih.gov/pmc/articles/pmc3326736/10.7150/thno.3509 PMC3326736 22509193

[cit50] Ajayan P. M., Ebbesen T. W. (1997). Nanometre-size tubes of carbon. Rep. Prog. Phys..

[cit51] Chico L., Crespi V. H., Benedict L. X., Louie S. G., Cohen M. L. (1996). Pure carbon nanoscale devices: Nanotube heterojunctions. Phys. Rev. Lett..

[cit52] Ibrahim K. S. (2013). Carbon nanotubes-properties and applications: a review. Carbon Lett..

[cit53] Aqel A. (2012). *et al.*, Carbon Nanotubes, Science and Technology Part (I) Structure, Synthesis and Characterisation. Arab. J. Chem..

[cit54] Terrones M. (2003). Science and Technology of the Twenty-First Century: Synthesis, Properties, and Applications of Carbon Nanotubes. Annu. Rev. Mater. Res..

[cit55] Dresselhaus M. , Dresselhaus G., and Eklund P., Science of fullerenes and carbon nanotubes: their properties and applications, 1996, accessed, Mar. 12, 2022, https://books.google.com.pk/books?hl=en&lr=&id=T8NLqyOMZ50C&oi=fnd&pg=PP2&dq=Dresselhaus,+M.+S

[cit56] Khan R., Ilyas Khan M., Khaloufa Almesfer M., Elkhaleefa A., Hassan Ali I., Ullah A., Rahman N., Sohail M., Khan A. A., Khan A. (2022). The structural and dilute magnetic properties of (Co, Li) co-doped-ZnO semiconductor nanoparticles. MRS Commun..

[cit57] Oberlin A. , Endo M. and T. K.-J., Filamentous growth of carbon through benzene decomposition, Elsevier, 1976, accessed, Mar. 12, 2022, https://www.sciencedirect.com/science/article/pii/0022024876901159

[cit58] Iijima S. (1991). Helical microtubules of graphitic carbon. Nature.

[cit59] Hamada N., Sawada S. I., Oshiyama A. (1992). New one-dimensional conductors: Graphitic microtubules. Phys. Rev. Lett..

[cit60] Saito R., Fujita M., Dresselhaus G., Dresselhaus M. S. (1992). Electronic structure of graphene tubules based on C60. Phys. Rev. B: Condens. Matter Mater. Phys..

[cit61] Saito R., Fujita M., Dresselhaus G., Dresselhaus M. S. (1992). Electronic structure of graphene tubules based on C60. Phys. Rev. B.

[cit62] Hirsch M. J. and Holcomb D. F., 18th International Conference on the Physics of Semiconductors: Stockholm, Sweden, August 11–15, 1986, Laboratory of Atomic and Solid State Physics, Cornell University, Ithaca, New York 14853, USA, vol. 2, World Scientific, 1987

[cit63] Ibrahim K. S. (2013). Carbon nanotubes–properties and applications: a review. Carbon Lett..

[cit64] Kim Y. A., Yang K.-S., Muramatsu H., Hayashi T., Endo M., Terrones M., Dresselhaus M. S. (2014). Double-walled carbon nanotubes: synthesis, structural characterization, and application. Carbon Lett..

[cit65] Winkin N., Gierth U., Schneider M. (2016). Nanomaterial-modified Flexible Micro-electrode Array by Electrophoretic Deposition of Carbon Nanotubes. J. Biochips Tissue Chips.

[cit66] Zhao Q., Gan Z., Zhuang Q. (2002). Electrochemical sensors based on carbon nanotubes. Electroanalysis.

[cit67] Geim A. K., Novoselov K. S. (2009). The rise of graphene. Nanosci. Nanotechnol..

[cit68] Geim A. K. (2009). Graphene: Status and prospects. Science.

[cit69] Novoselov K. S. (2004). *et al.*, Electric Field Effect in Atomically Thin Carbon Films. Science.

[cit70] Miller D. L. (2009). *et al.*, Observing the quantization of zero mass carriers in graphene. Science.

[cit71] Wang X., Zhi L., Müllen K. (2008). Transparent, conductive graphene electrodes for dye-sensitized solar cells. Nano Lett..

[cit72] Lee C., Wei X., Kysar J. W., Hone J. (2008). Measurement of the elastic properties and intrinsic strength of monolayer graphene. Science.

[cit73] Du X. , Skachko I., Barker A., E. A.-N., Approaching ballistic transport in suspended graphene, accessed: Feb. 04, 2022, https://www.nature.com/articles/nnano.2008.199 10.1038/nnano.2008.199 18685637

[cit74] Wang S., Ang P. K., Wang Z., Tang A. L. L., Thong J. T. L., Loh K. P. (2010). High mobility, printable, and solution-processed graphene electronics. Nano Lett..

[cit75] Jung J. H., Cheon D. S., Liu F., Lee K. B., Seo T. S. (2010). A graphene oxide based immuno-biosensor for pathogen detection. Angew. Chem., Int. Ed..

[cit76] He Q. (2010). *et al.*, Centimeter-long and large-scale micropatterns of reduced graphene oxide films: Fabrication and sensing applications. ACS Nano.

[cit77] Yin Z. (2010). *et al.*, Electrochemical
deposition of ZnO nanorods on transparent reduced graphene oxide electrodes for hybrid solar cells. Small.

[cit78] Hammel E. (2004). *et al.*, Carbon nanofibers for composite applications. Carbon.

[cit79] Wang K., Wang Y., Wang Y., Hosono E., Zhou H. (2009). Mesoporous carbon nanofibers for supercapacitor application. J. Phys. Chem. C.

[cit80] Rodriguez N. M. (1993). A review of catalytically grown carbon nanofibers. J. Mater. Res..

[cit81] Rauti R., Musto M., Bosi S., Prato M., Ballerini L. (2019). Properties and behavior of carbon nanomaterials when interfacing neuronal cells: How far have we come?. Carbon.

[cit82] Cheng H. Y., Zhu Y. A., Sui Z. J., Zhou X. G., Chen D. (2012). Modeling of fishbone-type carbon nanofibers with cone-helix structures. Carbon.

[cit83] International Carbon Black Association (ICBA) , Carbon Black User's Guide: Safety, Health, & Environmental Information, International Carbon Black Association (ICBA), 2004

[cit84] McCunney R. J. , Muranko H. J., Long C. M., Hamade A. K., Valberg P. A. and Morfeld P., Carbon black, in Patty's Toxicology, ed. Bingham E. and Cohrssen B., sixth edn, vol. 5, John Wiley & Sons, Inc., 2012, p. 429–453

[cit85] International Agency for Research on Cancer (IARC) , IARC Monographs on the Evaluation of Carcinogenic Risks to Humans, in Carbon Black, Titanium Dioxide, and Talc, World Health Organization (WHO), Lyon, France, 2010, vol. 93 PMC4781574 21449489

[cit86] Wang M.-J. , Gray C. A., Reznek S. A., Mahmud K., and Kutsovsky Y., Carbon Black, Kirk-Othmer Encyclopedia of Chemical Technology, 2003, 10.1002/0471238961.0301180204011414.A01.PUB2

[cit87] S. I. , nature, 1991, Helical microtubules of graphitic carbon, accessed: Feb. 05, 2022, https://www.nature.com/articles/354056a0

[cit88] Kuchibhatla S. , Karakoti A. and D. B.-P., One dimensional nanostructured material, Elsevier, 2007, accessed: Feb. 05, 2022, https://www.sciencedirect.com/science/article/pii/S0079642506000417

[cit89] Tiwari J. N., Tiwari R. N., Kim K. S. (2012). Zero-dimensional, one-dimensional, two-dimensional and three-dimensional nanostructured materials for advanced electrochemical energy devices. Prog. Mater. Sci..

[cit90] Hickey R. J., Meng X., Zhang P., Park S. J. (2013). Low-dimensional nanoparticle clustering in polymer micelles and their transverse relativity rates. ACS Nano.

[cit91] Gopi S., Amalraj A., Thomas S. (2016). Effective Drug Delivery System of Biopolymers Based on Nanomaterials and Hydrogels – A Review. Drug Des..

[cit92] Arroyo C. R. (2016). *et al.*, Reliable Tools for Quantifying the Morphological Properties at the Nanoscale. Biol. Med..

[cit93] Alivisatos P. (2004). The use of nanocrystals in biological detection. Nat. Biotechnol..

[cit94] Cui Y., Wei Q., Park H., Lieber C. M. (2001). Nanowire nanosensors for highly sensitive and selective detection of biological and chemical species. Science.

[cit95] Hangarter C. M. (2010). *et al.*, Conducting Polymer Nanowires for Chemiresistive and FET-based Bio/Chemical Sensors. J. Mater. Chem..

[cit96] Duan X., Huang Y., Cui Y., Wang J., Lieber C. M. (2001). Indium phosphide nanowires as building blocks for nanoscale electronic and optoelectronic devices. Nature.

[cit97] Cui Y., Lieber C. M. (2001). Functional nanoscale electronic devices assembled using silicon nanowire building blocks. Science.

[cit98] Xia Y. (2003). *et al.*, One-dimensional nanostructures: Synthesis, characterization, and applications. Adv. Mater..

[cit99] Xia Y., Yang P., Sun Y., Wu Y., Mayers B., Gates B., Yin Y., Kim F., Yan H. (2003). One-Dimensional Nanostructures: Synthesis, Characterization, and Applications. ChemInform.

[cit100] Machín A. (2021). *et al.*, One-Dimensional (1D) Nanostructured Materials for Energy Applications. Materials.

[cit101] Pan J., Shen H., Mathur S. (2012). One-dimensional SnO_2_ nanostructures: Synthesis and applications. J. Nanotechnol..

[cit102] Nasiri N., Clarke C. (2019). Nanostructured gas sensors for medical and health applications: Low to high dimensional materials. Biosensors.

[cit103] Al-Kaysi R. O., Ghaddar T. H., Guirado G. (2009). Fabrication of one-dimensional organic nanostructures using anodic aluminum oxide templates. J. Nanomater..

[cit104] Jun Y. , Seo J., Oh S., *et al.*, Recent advances in the shape control of inorganic nano-building blocks, Coord. Chem. Rev., 2005, accessed: Feb. 05, 2022, https://www.sciencedirect.com/science/article/pii/S0010854504003121

[cit105] Kim K. , *et al.*, Large-scale pattern growth of graphene films for stretchable transparent electrodes, accessed: Feb. 05, 2022, https://www.nature.com/articles/nature07719 10.1038/nature07719 19145232

[cit106] Bae S. , *et al.*, Roll-to-roll production of 30-inch graphene films for transparent electrodes, accessed: Feb. 05, 2022, https://www.nature.com/articles/nnano.2010.132 10.1038/nnano.2010.132 20562870

[cit107] Pradhan D., Leung K. T. (2008). Vertical growth of two-dimensional zinc oxide nanostructures on ITO-coated glass: Effects of deposition temperature and deposition time. J. Phys. Chem. C.

[cit108] Jibowu T. (2016). The Formation of Doxorubicin Loaded Targeted Nanoparticles Using Nanoprecipitation, Double Emulsion and Single Emulsion for Cancer Treatment. J. Nanomed. Nanotechnol..

[cit109] Rasch F. (2019). *et al.*, Wet-Chemical Assembly of 2D Nanomaterials into Lightweight, Microtube-Shaped, and Macroscopic 3D Networks. ACS Appl. Mater. Interfaces.

[cit110] Chen Y. (2018). *et al.*, Two-Dimensional Metal Nanomaterials: Synthesis, Properties, and Applications. Chem. Rev..

[cit111] Szuplewska A. (2019). *et al.*, Multilayered stable 2D nano-sheets of Ti2NT*x* MXene: Synthesis, characterization, and anticancer activity. J. Nanobiotechnol..

[cit112] Shen Q., Jiang L., Zhang H., Min Q., Hou W., Zhu J. J. (2008). Three-dimensional dendritic Pt nanostructures: Sonoelectrochemical synthesis and electrochemical applications. J. Phys. Chem. C.

[cit113] Teng X., Liang X., Maksimuk S., Yang H. (2006). Synthesis of porous platinum nanoparticles. Small.

[cit114] Sreelakshmy V., Deepa M. K., Mridula P. (2016). Green Synthesis of Silver Nanoparticles from Glycyrrhiza Glabra Root Extract for the Treatment of Gastric Ulcer. J. Dev. Drugs.

[cit115] Typical SEM image of the 3-D superlattice of Pd NPs. (a) SEM image of…|Download Scientific Diagram, https://www.researchgate.net/figure/Typical-SEM-image-of-the-3-D-superlattice-of-Pd-NPs-a-SEM-image-of-superlattice_fig4_227395223, accessed May 04, 2022

[cit116] Low and high magnification SEM images of the as-synthesized different…|Download Scientific Diagram, https://www.researchgate.net/figure/Low-and-high-magnification-SEM-images-of-the-as-synthesized-different-CuO-nanostructures_fig2_299382164, accessed May 04, 2022

[cit117] A clearly depicting needle shaped nanorods structure. Each needle…|Download Scientific Diagram, https://www.researchgate.net/figure/a-clearly-depicting-needle-shaped-nanorods-structure-Each-needle-shaped-nanorods-appears_fig1_223334377, accessed May 04, 2022

[cit118] Ruffini A. , Sprio S., Preti L., and Tampieri A., Synthesis of Nanostructured Hydroxyapatite *via* Controlled Hydrothermal Route, Biomaterial-supported Tissue Reconstruction or Regeneration, 2019, 10.5772/INTECHOPEN.85091

[cit119] Theerthagiri J. (2019). *et al.*, A review on ZnO nanostructured materials: Energy, environmental and biological applications. Nanotechnology.

[cit120] Sharma D. K., Shukla S., Sharma K. K., Kumar V. (2022). A review on ZnO: Fundamental properties and applications. Mater. Today: Proc.

[cit121] Liu W. (2010). *et al.*, Compact biocompatible quantum dots *via* RAFT-mediated synthesis of imidazole-based random copolymer ligand. J. Am. Chem. Soc..

[cit122] Hoshino A., Fujioka K., Oku T. (2004). *et al.*, Quantum dots targeted to the assigned organelle in living cells. Microbiol. Immunol..

[cit123] Weintraub B., Zhou Z., Li Y., Deng Y. (2010). Solution synthesis of one-dimensional ZnO nanomaterials and their applications. Nanoscale.

[cit124] Xia Y., Yang P., Sun Y. (2003). *et al.*, One-dimensional nano structures: synthesis, characterization, and applications. Adv. Mater..

[cit125] Yi G. C., Wang C., Park W. I. (2005). ZnO nanorods: synthesis, characterization and applications. Semicond. Sci. Technol..

[cit126] Banerjee D. (2003). *et al.*, Large-quantity free-standing ZnO nanowires. Appl. Phys. Lett..

[cit127] Hahn Y. B. (2011). Zinc oxide nanostructures and their applications. Korean J. Chem. Eng..

[cit128] Frade T., Melo Jorge M. E., Gomes A. (2012). One-dimensional ZnO nanostructured films: Effect of oxide nanoparticles. Mater. Lett..

[cit129] Wahab R., Ansari S. G., Kim Y. S., Seo H. K., Shin H. S. (2007). Room temperature synthesis of needle-shaped ZnO nanorods *via* sonochemical method. Appl. Surf. Sci..

[cit130] Kong X., Ding Y., Yang R., Wang Z. L. (2004). Single-crystal nanorings formed by epitaxial self-coiling of polar-nanobelts. Science.

[cit131] Pan Z. W., Dai Z. R., Wang Z. L. (2001). Nanobelts of semiconducting oxides. Science.

[cit132] Wu J. J., Liu S. C., Wu C. T., Chen K. H., Chenm L. C. (2002). Heterostructures of ZnO–Zn coaxial nanocables and ZnO nanotubes. Appl. Phys. Lett..

[cit133] Chen W. J., Liu W. L., Hsieh S. H., Tsai T. K. (2007). Preparation of nanosized ZnO using α brass. Appl. Surf. Sci..

[cit134] Liu J., Huang X., Duan J., Ai H., Tu P. (2005). A low-temperature synthesis of multiwhisker-based zinc oxide micron crystals. Mater. Lett..

[cit135] Huang Y., He J., Zhang Y., Dai Y., Gu Y., Wang S., Zhou C. (2006). Morphology, structures and properties of ZnO nanobelts fabricated by Zn-powder evaporation without catalyst at lower temperature. J. Mater. Sci..

[cit136] Nikoobakht B., Wang X., Herzing A., Shi J. (2013). Scable synthesis and device integration of self-registered one-dimensional zinc oxide nanostructures and related materials. Chem. Soc. Rev..

[cit137] Tien L. C., Pearton S. J., Norton D. P., Ren F. (2008). Synthesis and microstructure of vertically aligned ZnO nanowires grown by high-pressure-assisted pulsed-laser deposition. J. Mater. Sci..

[cit138] Cui J. (2012). Zinc oxide nanowires. Mater. Charact..

[cit139] Xu T., Ji P., He M., Li J. (2012). Growth and structure of pure ZnO micro/nanocombs. J. Nanomater..

[cit140] Chiua W. S., Khiew P. S., Clokea M., Isaa D., Tana T. K., Radimanb S., Abd-Shukorb R., Abd-Hamid M. A., Huangc N. M., Limd H. N. (2010). *et al.*, Photocatalytic study of two-dimensional ZnO nanopellets in the decomposition of methylene blue. Chem. Eng. J..

[cit141] Jose-Yacaman M., Gutierrez-Wing C., Miki M., Yang D. Q., Piyakis K. N., Sacher E. (2005). Surface diffusion and coalescence of mobile metal nanoparticles. J. Phys. Chem. B.

[cit142] Polshettiwar V., Baruwati B., Varma R. S. (2009). Self-assembly of metal oxides into three-dimensional nanostructures: Synthesis and application in catalysis. ACS Nano.

[cit143] Xie Q., Dai Z., Liang J., Xu L., Yu W., Qian Y. (2005). Synthesis of ZnO three-dimensional architectures and their optical properties. Solid State Commun..

[cit144] Liu J., Huang X., Li Y., Sulieman K. M., Sun F., He X. (2006). Selective growth and properties of zinc oxide nanostructures. Scr. Mater..

[cit145] Bitenc M., Orel Z. C. (2009). Synthesis and characterization of crystalline hexagonal bipods of zinc oxide. Mater. Res. Bull..

[cit146] Examples of zinc oxide structure: flower (a); rods (b); wires (c)…|Download Scientific Diagram, https://www.researchgate.net/figure/Examples-of-zinc-oxide-structure-flower-a-rods-b-wires-c-d-created_fig1_263005483, accessed May 04, 2022

[cit147] Schwartz D. A., Norberg N. S., Nguyen Q. P., Parker J. M., Gamelin D. R. (2003). Magnetic Quantum Dots: Synthesis, Spectroscopy, and Magnetism of Co^2+^- and Ni^2+^-Doped ZnO Nanocrystals. J. Am. Chem. Soc..

[cit148] (a) Single crystal seamless nanoring formed by loop-by-loop coiling…|Download Scientific Diagram, https://www.researchgate.net/figure/a-Single-crystal-seamless-nanoring-formed-by-loop-by-loop-coiling-of-a-polar-nanobelt_fig6_226912813, accessed Apr. 25, 2022

[cit149] Gao P. X., Wang Z. L. (2005). High-Yield Synthesis of Single-Crystal Nanosprings of ZnO. Small.

[cit150] Xie Q., Ma Y., Zeng D., Wang L., Yue G., Peng D. L. (2015). Facile fabrication of various zinc-nickel citrate microspheres and their transformation to ZnO-NiO hybrid microspheres with excellent lithium storage properties. Sci. Rep..

[cit151] FE-SEM images of (a) ZnO nanoparticles, (b) ZnO nanoparticles…|Download Scientific Diagram, https://www.researchgate.net/figure/FE-SEM-images-of-a-ZnO-nanoparticles-b-ZnO-nanoparticles-hydrothermal-c-ZnO_fig1_267742691, accessed May 03, 2022

[cit152] Zhang Y., Ram M. K., Stefanakos E. K., Goswami D. Y. (2012). Synthesis, characterization, and applications of ZnO nanowires. J. Nanomater..

[cit153] Hahn Y. B. (2011). Zinc oxide nanostructures and their applications. Korean J. Chem. Eng..

[cit154] Consonni V., Briscoe J., Kärber E., Li X., Cossuet T. (2019). ZnO nanowires for solar cells: A comprehensive review. Nanotechnology.

[cit155] Handbook of Semiconductor Nanostructures and Nanodevices, ed. A. A. Balandin and K. L. Wang, American Scientific Publishers, 2005

[cit156] Metal Oxide Nanostructures and Their Applications, ed. A. Umar and Y.-B. Hahn, American Scientific Publishers, 2010

[cit157] Reeber R. R. (1970). J. Appl. Phys..

[cit158] Zinc oxide – Wikipedia, https://en.wikipedia.org/wiki/Zinc_oxide, accessed May 04, 2022

[cit159] Piezoelectronic properties of ZnO and its potential to power nanotech’ by Faye Jones…|Van Heuvelen Lab, http://blogs.hmc.edu/vanheuvelen/the-elements/piezoelectronic-properties-of-zno-and-its-potential-to-power-nanotech-by-faye-jones/, accessed May 04, 2022

[cit160] Nahhas A. M. (2018). Recent Advances of ZnO Based Nanowires and Nanorods Devices. Am. J. Nanomater..

[cit161] Kuoni A., Holzherr R., Boillat M., de Rooij N. F. (2003). J. Micromech. Microeng..

[cit162] Sharma D. K., Shukla S., Sharma K. K., Kumar V. (2022). A review on ZnO: Fundamental properties and applications. Mater. Today: Proc..

[cit163] Dulub O., Boatner L. A., Diebold U. (2002). Surf. Sci..

[cit164] Phillips J. C. , Bonds and Bands in Semiconductors, Academic Press, New York, 1973

[cit165] Segawa Y., Ohtomo A., Kawasaki M., Koinuma H., Tang Z. K., Yu P., Wong G. K. L. (1997). Phys. Stat. Sol..

[cit166] Khan I., Khan S., Nongjai R., Ahmed H., Khan W. (2013). Hydrothermal synthesis of zinc oxide powders with controllable morphology. Opt. Mater..

[cit167] Xu H., Wang H., Zhang Y., He W., Zhu M., Wang B. (2004). *et al.*, Structural and optical properties of gelcombustion synthesized Zr doped ZnO nanoparticles. Ceram. Int..

[cit168] Kung S., Sreenivas K. (2016). Defect free C-axis oriented zinc oxide (ZnO) films grown at room temperature using RF magnetron sputtering. AIP Conf. Proc..

[cit169] Li Y., Bando Y., Golberg D. (2004). ZnO nanoneedles with tip surface perturbations: Excellent field emitters. Appl. Phys. Lett..

[cit170] Zhang M., Gao X., Barra A., Chang P., Huang L., Hellwarth R. (2015). *et al.*, Coreshell structured Si/ZnO photovoltaics. Mater. Lett..

[cit171] Sabah M., Hassan M., Naser M., Al-hardan H., Bououdina M. (2015). Fabrication of low-cost UV photo detector using ZnO nanorods grown onto nylon substrate. J. Mater. Sci..

[cit172] Pandya H., Chandra S., Vyas A. (2012). Integration of ZnO nanostructures with MEMS for ethanol sensor. Sens. Actuators, B.

[cit173] Taube A., Sochacki M., Kwietniewski N., Werbowy A., Gierałtowska S., Wachnicki L. (2017). *et al.*, Electrical properties of isotype and anisotype ZnO/4H-SiC heterojunction diodes. Appl. Phys. Lett..

[cit174] Baruah S., Dutta J. (2009). Hydrothermal growth of ZnO nanostructures. Sci. Technol. Adv. Mater..

[cit175] Porter F. , Zinc Handbook: Properties, Processing, and Use in Design, CRC Press, 1991

[cit176] Subramanyam T. K., Srinivasulu Naidu B., Uthanna S. (2000). Physical Properties of Zinc Oxide Films Prepared by DC Reactive Magnetron Sputtering at Different Sputtering Pressures. Cryst. Res. Technol..

[cit177] Gao P. X., Song J. H., Liu J., Wang Z. L. (2007). Nanowire piezoelectric nanogenerators on plastic substrates as flexible power sources for nanodevices. Adv. Mater..

[cit178] Janotti A., van de Walle C. G. (2009). Fundamentals of zinc oxide as a semiconductor. Rep. Prog. Phys..

[cit179] Djurišić A. B., Leung Y. H. (2006). Optical properties of ZnO nanostructures. Small.

[cit180] Z. W. , Zinc oxide nanostructures: growth, properties and applications, J. Phys.: Condens. Matter, 2004, accessed: Feb. 18, 2022, https://iopscience.iop.org/article/10.1088/0953-8984/16/25/R01/meta

[cit181] J. C.-M. , Zinc oxide nanowires, Elsevier, 2012, accessed: Feb. 18, 2022, https://www.sciencedirect.com/science/article/pii/S1044580311002701

[cit182] Willander M., Nur O., Zhao Q. X., Yang L. L., Lorenz M., Cao B. Q., Pérez J. Z., Czekalla C., Zimmermann G., Grundmann M., Bakin A., Behrends A., AlSuleiman M., El-Shaer A., Mofor A. C., Postels B., Waag A., Boukos N., Travlos A., Kwack H. S., Guinard J., Si Dang D. L. (2009). Zinc oxide nanorod based photonic devices: recent progress in growth, light emitting diodes and lasers. Nanotechnology.

[cit183] Li J. (2017). *et al.*, Structural and optical properties of nano-crystalline ZnO thin films synthesized by sol–gel method. J. Sol-Gel Sci. Technol..

[cit184] Kara R., Mentar L., Azizi A. (2020). Synthesis and characterization of Mg-doped ZnO thin-films electrochemically grown on FTO substrates for optoelectronic applications. RSC Adv..

[cit185] Kafle B. P. (2016). Thickness Dependence of Optical and Electrical Properties of Zinc Oxide Thin Films. Mater. Sci. Eng..

[cit186] Optical transmittance spectra of ZnO films of different thicknesses…|Download Scientific Diagram, https://www.researchgate.net/figure/Optical-transmittance-spectra-of-ZnO-films-of-different-thicknesses_fig2_264673085, accessed May 17, 2022

[cit187] Optical absorbance spectra of ZnO thin films…|Download Scientific Diagram, https://www.researchgate.net/figure/Color-online-Optical-absorbance-spectra-of-ZnO-thin-films_fig1_233883965, accessed May 17, 2022

[cit188] Elthair N. A., Mustafa E. M., Elbadawi A. A., Elthair N. A., Mustafa E. M., Elbadawi A. A. (2020). The Electrical and Optical Properties of Zn_0.5_Li_2x_Mg_0.5-*x*_Fe_2_O_4_ Lithium Doped Nanoparticle Prepared by Coprecipitation Method. Open J. Appl. Sci..

[cit189] Han S. J., Jang T. H., Kim Y. B., Park B. G., Park J. H., Jeong Y. H. (2003). Magnetism in Mn-doped ZnO bulk samples prepared by solid state reaction. Appl. Phys. Lett..

[cit190] Magnetization *versus* magnetic field (M-H) curves of ZnO films with…|Download Scientific Diagram, https://www.researchgate.net/figure/Magnetization-versus-magnetic-field-M-H-curves-of-ZnO-films-with-various-Gd_fig9_234984970, accessed May 17, 2022

[cit191] Kumar D. R., Ranjith K. S., Nivedita L. R., Kumar R. T. R. (2017). Effect of samarium doping on structural, optical and magnetic properties of vertically aligned ZnO nanorod arrays. J. Rare Earths.

[cit192] Yahmadi B., Kamoun O., Alhalaili B., Alleg S., Vidu R., Turki N. K. (2020). Physical Investigations of (Co, Mn) Co-Doped ZnO Nanocrystalline Films. Nanomaterials.

[cit193] Obeid M. M., Jappor H. R., Al-Marzoki K., Al-Hydary I. A., Edrees S. J., Shukur M. M. (2019). Unraveling the effect of Gd doping on the structural, optical, and magnetic properties of ZnO based diluted magnetic semiconductor nanorods. RSC Adv..

[cit194] The measured room temperature magnetization curve for the 0.1% Mn doped…|Download Scientific Diagram, https://www.researchgate.net/figure/The-measured-room-temperature-magnetization-curve-for-the-01-Mn-doped-ZnO-Blue-curve_fig4_259005255, accessed May 17, 2022

[cit195] Zhang X., Zhang W., Zhang X., Xu X., Meng F., Tang C. C. (2014). Defects induced room temperature ferromagnetism in ZnO thin films. Adv. Condens. Matter Phys..

[cit196] MPCVD-ZnO Zinc Oxide Nano-rod Synthesis System – Microphase Co., Ltd…|New materials, CNT applications, Others, http://www.microphase.jp/EN-equipment-402.html, accessed May 04, 2022

[cit197] Zhang Y., Ram M. K., Stefanakos E. K., Goswami D. Y. (2012). Synthesis, characterization, and applications of ZnO nanowires. J. Nanomater..

[cit198] Characterization of Zinc Oxide Nanorod Samples, https://www.azonano.com/article.aspx?ArticleID=2509, accessed Apr. 25, 2022

[cit199] Fang F. , Ng A. M. C., Chen X. Y., Djurišić A. B., and Chan W. K., Ce-doped ZnO nanorods by electrodeposition, INEC 2010, 2010 3rd International Nanoelectronics Conference, Proceedings, 2010, pp. 460–461, 10.1109/INEC.2010.5424805

[cit200] Nahhas A. M. (2018). Recent Advances of ZnO Based Nanowires and Nanorods Devices. Am. J. Nanomater..

[cit201] SEM images of the ZnO nanorods from the first group…|Download Scientific Diagram, https://www.researchgate.net/figure/SEM-images-of-the-ZnO-nanorods-from-the-first-group_fig5_269031383, accessed Apr. 25, 2022

[cit202] She G., Zhang X., Shi W., Fan X., Chang J. C. (2007). Electrochemical/chemical synthesis of highly-oriented single-crystal ZnO nanotube arrays on transparent conductive substrates. Electrochem. Commun..

[cit203] Yang K. (2009). *et al.*, ZnO nanotube arrays as biosensors for glucose. J. Phys. Chem. C.

[cit204] She G. W. (2008). *et al.*, Controlled synthesis of oriented single-crystal ZnO nanotube arrays on transparent conductive substrates. Appl. Phys. Lett..

[cit205] Liu Z., Liu C., Ya J., Lei E. (2011). Controlled synthesis of ZnO and TiO_2_ nanotubes by chemical method and their application in dye-sensitized solar cells. Renewable Energy.

[cit206] Wang H. (2010). *et al.*, Surfactant-assisted *in situ* chemical etching for the general synthesis of ZnO nanotubes array. Nanoscale Res. Lett..

[cit207] Maiti U. N., Nandy S., Karan S., Mallik B., Chattopadhyay K. K. (2008). Enhanced optical and field emission properties of CTAB-assisted hydrothermal grown ZnO nanorods. Appl. Surf. Sci..

[cit208] Zhai H. J., Wu W. H., Lu F., Wang H. S., Wang C. (2008). Effects of ammonia and cetyltrimethylammonium bromide (CTAB) on morphologies of ZnO nano- and micromaterials under solvothermal process. Mater. Chem. Phys..

[cit209] Dev A., Panda S. K., Kar S., Chakrabarti S., Chaudhuri S. (2006). Surfactant-Assisted Route to Synthesize Well-Aligned ZnO Nanorod Arrays on Sol–Gel-Derived ZnO Thin Films. J. Phys. Chem. B.

[cit210] Controlling the Growth and Luminescence Properties of Well-Faceted ZnO Nanorods…|N. Elizondo, https://www.academia.edu/29159840/Controlling_the_Growth_and_Luminescence_Properties_of_Well_Faceted_ZnO_Nanorods, accessed May 05, 2022

[cit211] Kim Y. H., Moon D. G., Han J. I. (2004). Organic TFT array on a paper substrate. IEEE Electron Device Lett..

[cit212] Manekkathodi A., Lu M. Y., Wang C. W., Chen L. J. (2010). Direct growth of aligned zinc oxide nanorods on paper substrates for low-cost flexible electronics. Adv. Mater..

[cit213] Pacholski C., Kornowski A., Weller H. (2002). Self-assembly of ZnO: from nanodots to nanorods. Angew. Chem..

[cit214] Elias J., Tena-Zaera R., Wang G. Y., Levy-Cl C. (2008). Conversion of ZnO nanowires into nanotubes with tailored dimensions. Chem. Mater..

[cit215] Han J., Fan F., Xu C. (2010). *et al.*, ZnO nanotube-based dye-sensitized solar cell and its application in self-powered devices. Nanotechnology.

[cit216] Soomro M. Y., Hussain I., Bano N., Hussain S., Nur O., Willander M. (2012). Enhancement of zinc interstitials in ZnO nanotubes grown on glass substrate by the hydrothermal method. Appl. Phys. A: Mater. Sci. Process..

[cit217] Schlur L., Calado J. R., Spitzer D. (2018). Synthesis of zinc oxide nanorods or nanotubes on one side of a microcantilever. R. Soc. Open Sci..

[cit218] ZnO nanotubes (a) top and (b) inclined views. Reprinted with permission…|Download Scientific Diagram, https://www.researchgate.net/figure/ZnO-nanotubes-a-top-and-b-inclined-views-Reprinted-with-permission-from-23b-B_fig4_260095210, accessed Apr. 25, 2022

[cit219] ZnO nanotubes…|Everything about solar energy, http://energyprofessionalsymposium.com/?p=10687, accessed Apr. 25, 2022

[cit220] Soomro M. Y. (2012). *et al.*, Growth, structural and optical characterization of ZnO nanotubes on disposable-flexible paper substrates by low-temperature chemical method. J. Nanotechnol..

[cit221] Liang Z., Gao R., Lan J.-L., Wiranwetchayan O., Zhang Q., Li C., Cao G. (2013). Growth of vertically aligned ZnO nanowalls for inverted polymer solar cells. J. Nanotechnol..

[cit222] Pan Z. W., Dai Z. R., Wang Z. L. (2001). Nanobelts of semiconducting oxides. Science.

[cit223] Hughes W. L., Wang Z. L. (2004). Formation of piezoelectric single-crystal nanorings and nanobows. J. Am. Chem. Soc..

[cit224] Ding Y., Kong X. Y., Wang Z. L. (2004). Doping and planar defects in the formation of single-crystal ZnO nanorings. Phys. Rev. B: Condens. Matter Mater. Phys..

[cit225] Kong X. Y., Ding Y., Yang R., Wang Z. L. (2004). Single-Crystal Nanorings Formed by Epitaxial Self-Coiling of Polar Nanobelts. Science.

[cit226] Yousefi R. (2015). Effects of Sn atoms on formation of ZnO nanorings. CrystEngComm.

[cit227] Li S., Lin P., Lee C. (2004). *et al.*, Effect of Sn dopant on the properties of ZnO nanowires. J. Phys. D: Appl. Phys..

[cit228] Deng R., Zhang X., Zhang E., Liang Y., Liu Z., Xu H. Y., Hark S. (2007). J. Phys. Chem. C.

[cit229] Wang Z. L. (2004). Zinc oxide nanostructures: Growth, properties and applications. J. Phys.: Condens. Matter.

[cit230] SEM image recorded from an as-synthesized sample, showing the…|Download Scientific Diagram, https://www.researchgate.net/figure/a-SEM-image-recorded-from-an-as-synthesized-sample-showing-the-presence-of-ZnO_fig1_235540553, accessed Apr. 25, 2022

[cit231] Zinc Oxide Nanostructures: Growth, Properties and Applications, https://phys.org/news/2004-06-zinc-oxide-nanostructures-growth-properties.html, accessed Apr. 25, 2022

[cit232] Cao X., Wang N., Wang L., Guo L. (2009). Porous ZnO nanobelts: synthesis, mechanism, and morphological evolutions. J. Nanopart. Res..

[cit233] Zeng X., Yang J., Shi L., Li L., Gao M. (2014). Synthesis of multi-shelled ZnO hollow microspheres and their improved photocatalytic activity. Nanoscale Res. Lett..

[cit234] Wang Q., Li H., Chen L., Huang X. (2001). Monodispersed hard carbon spherules with uniform nanopores. Carbon.

[cit235] Sun X., Li Y. (2004). Colloidal Carbon Spheres and Their Core/Shell Structures with Noble-Metal Nanoparticles. Angew. Chem., Int. Ed..

[cit236] Yu L., Li Z. (2019). Synthesis of ZnxCd1-xSe@ZnO Hollow Spheres in Different Sizes for Quantum Dots Sensitized Solar Cells Application. Nanomaterials.

[cit237] Chen X., Jing X., Wang J., Liu J., Song D., Liu L. (2013). Self-assembly of ZnO nanoparticles into hollow microspheres *via* a facile solvothermal route and their application as gas sensor. CrystEngComm.

[cit238] Scanning electron microscope image (SEM) of the ZnO hollow spheres…|Download Scientific Diagram, https://www.researchgate.net/figure/Fig-Scanning-electron-microscope-image-SEM-of-the-ZnO-hollow-spheres_fig1_325091035, accessed May 03, 2022

[cit239] Cho J. (2012). *et al.*, Sulfur-doped zinc oxide (ZnO) Nanostars: Synthesis and simulation of growth mechanism. Springer.

[cit240] Andrade G. R. S., Nascimento C. C., Lima Z. M., Teixeira-Neto E., Costa L. P., Gimenez I. F. (2017). Star-shaped ZnO/Ag hybrid nanostructures for enhanced photocatalysis and antibacterial activity. Appl. Surf. Sci..

[cit241] Shi R. (2012). *et al.*, Growth of flower-like ZnO *via* surfactant -free hydrothermal synthesis on ITO substrate at low temperature. CrystEngComm.

[cit242] Wahab R. (2007). *et al.*, Low temperature solution synthesis and characterization of ZnO nano-flowers. Mater. Res. Bull..

[cit243] Naveed Ul Haq A., Nadhman A., Ullah I., Mustafa G., Yasinzai M., Khan I. (2017). Synthesis Approaches of Zinc Oxide Nanoparticles: The Dilemma of Ecotoxicity. J. Nanomater..

[cit244] Pergolese B., Muniz-Miranda M., Bigotto A. (2016). Optimization of process variables for the biosynthesis of silver nanoparticles by Aspergillus wentii using statistical experimental design. Langmuir.

[cit245] Purwaningsih S. Y., Pratapa S., Triwikantoro T., Darminto (2016). Nano-sized ZnO powders prepared by co-precipitation method with various pH. AIP Conf. Proc..

[cit246] Sharma G. (2019). *et al.*, Novel development of nanoparticles to bimetallic nanoparticles and their composites: A review. J. King Saud Univ., Sci..

[cit247] Asmatulu R. , Nanocoatings for corrosion protection of aerospace alloys, Corrosion Protection and Control Using Nanomaterials, 2012, pp. 357–374, 10.1533/9780857095800.2.357

[cit248] Pantelic I. and Cuckovic B., Alkyl Polyglucosides: An emerging class of sugar surfactants, Alkyl Polyglucosides: From Natural-Origin Surfactants to Prospective Delivery Systems, 2014, pp. 1–19, 10.1533/9781908818775.1

[cit249] Yildirim Ö. A., Durucan C. (2010). Synthesis of zinc oxide nanoparticles elaborated by microemulsion method. J. Alloys Compd..

[cit250] Aneesh P. M. , Vanaja K. A., and Jayaraj M. K., Synthesis of ZnO nanoparticles by hydrothermal method, Nanophotonic Materials IV, 2007, vol. 6639, p. 66390J, 10.1117/12.730364

[cit251] Pan Z., Wang Y., Huang H., Ling Z., Dai Y., Ke S. (2015). Recent development on preparation of ceramic inks in ink-jet printing. Ceram. Int..

[cit252] Ghoshal T., Biswas S., Paul M., De S. K. (2009). Synthesis of ZnO nanoparticles by solvothermal method and their ammonia sensing properties. J. Nanosci. Nanotechnol..

[cit253] Ghaffari-Moghaddam M., Hadi-Dabanlou R. (2014). Plant mediated green synthesis and antibacterial activity of silver nanoparticles using Crataegus douglasii fruit extract. J. Ind. Eng. Chem..

[cit254] Jung D. S., Ko Y. N., Kang Y. C., bin Park S. (2014). Recent progress in electrode materials produced by spray pyrolysis for next-generation lithium-ion batteries. Adv. Powder Technol..

[cit255] Chang H., Tsai M. H. (2008). Synthesis and characterization of ZnO nanoparticles having prism shape by a novel gas condensation process. Rev. Adv. Mater. Sci..

[cit256] Salah N. (2011). *et al.*, High-energy ball milling technique for ZnO nanoparticles as antibacterial material. Int. J. Nanomed..

[cit257] Singh S. C., Gopal R. (2007). Zinc nanoparticles in solution by laser ablation technique. Bull. Mater. Sci..

[cit258] Sharma G. (2019). *et al.*, Novel development of nanoparticles to bimetallic nanoparticles and their composites: A review. J. King Saud Univ., Sci..

[cit259] Ijaz I., Gilani E., Nazir A., Bukhari A. (2020). Detail review on chemical, physical and green synthesis, classification, characterizations and applications of nanoparticles. Green Chem. Lett. Rev..

[cit260] Gunalan S., Sivaraj R., Rajendran V. (2012). Green synthesized ZnO nanoparticles against bacterial and fungal pathogens. Prog. Nat. Sci.: Mater. Int..

[cit261] Kołodziejczak-Radzimska A. (2014). *et al.*, Zinc oxide—from synthesis to application: a review. Materials.

[cit262] Das A., Wang D. Y., Leuteritz A., Subramaniam K., Greenwell H. C., Wagenknecht U., Heinrich G. (2011). Preparation of zinc oxide free, transparent rubber nanocomposites using a layered double hydroxide filler. J. Mater. Chem..

[cit263] Yuan Z., Zhou W., Hu T., Chen Y., Li F., Xu Z., Wang X. (2011). Fabrication and properties of silicone rubber/ZnO nanocomposites *via in situ* surface hydrosilylation. Surf. Rev. Lett..

[cit264] Mandal U. K., Tripathy D. K., De S. K. (1996). Dynamic mechanical spectroscopic studies on plasticization of an ionic elastomer based on carboxylated nitrile rubber by ammonia. Polymer.

[cit265] Ibarra L., Marcos-Fernandez A., Alzorriz M. (2002). Mechanistic approach to the curing of carboxylated nitrile rubber (XNBR) by zinc peroxide/zinc oxide. Polymer.

[cit266] Chatterjee K., Naskar K. (2007). Development of thermoplastic elastomers based on maleated ethylene propylene rubber (n-EPM) and propylene (PP) by dynamic vulcanization. eXPRESS Polym. Lett..

[cit267] Hamed G. R., Hua K. C. (2004). Effect of zinc oxide particle size on the curing of carboxylated NBR and carboxylated SBR. Rubber Chem. Technol..

[cit268] Sabura Begum P. M., Mohammed Yusuff K. K., Joseph R. (2008). Preparation and use of nano zinc oxide in neoprene rubber. Int. J. Polym. Mater..

[cit269] Liu H., Yang D., Yang H., Zhang H., Zhang W., Fang Y., Liu Z., Tian L., Lin B., Yan J. (2013). *et al.*, Comparative study of respiratory tract immune toxicity induced by three sterilization nanoparticles: Silver, zinc oxide and titanium oxide. J. Hazard. Mater..

[cit270] Mirhosseini M., Firouzabadi F. (2012). Antibacterial activity of zinc oxide nanoparticle suspensions on food-borne pathogens. Int. J. Dairy Technol..

[cit271] Mason P. (2006). Physiological and medicinal zinc. Pharm. J..

[cit272] Newman M. D., Stotland M., Ellis J. I. (2009). The safety of nano sized particles in titanium dioxide and zinc oxide-based sunscreens. J. Am. Acad. Dermatol..

[cit273] Pirot F., Millet J., Kalia Y. N., Humbert P. (1996). *In vitro* study of percutaneous absorption, cutaneous bioavailability and bioequivalence of zinc and copper from five topical formulations. Skin Pharmacol..

[cit274] Lansdown A. B., Taylor A. (1997). Zinc and titanium oxides: Promising UV-absorbers, but what influence do they have on the intact skin?. Int. J. Cosmet. Sci..

[cit275] Cross S. E., Innes B., Roberts M. S., Tsuzuki T., Robertson T. A., McCormick P. (2007). Human skin penetration of sunscreen nanoparticles: *In vitro* assessment of novel micronized zinc oxide formulation. Skin Pharmacol..

[cit276] Yadav A., Prasad V., Kathe A. A., Raj S., Yadav D., Sundaramoorthy C., Vigneshwaran N. (2006). Functional finishing in cotton fabrics using zinc oxide nanoparticles. Bull. Mater. Sci..

[cit277] Mao Z., Shi Q., Zhang L., Cao H. (2009). The formation and UV-blocking property of needle-shaped ZnO nanorod on cotton fabric. Thin Solid Films.

[cit278] Becheri A., Maximilian D., Lo Nostro P., Baglioni P. (2008). Synthesis and characterization of zinc oxide nanoparticles: Application to textiles as UV-absorbers. J. Nanopart. Res..

[cit279] Wang R., Xin J. H., Tao X. M., Daoud W. A. (2004). ZnO nanorods grown on cotton fabrics at low temperature. Chem. Phys. Lett..

[cit280] Liu Y., Zhou J., Larbot A., Persin M. (2007). Preparation and characterization of nano-zinc oxide. J. Mater. Process. Technol..

[cit281] Mansouri S., Bourguiga R., Yakuphanoglu F. (2012). Analytic model for ZnO-thin film transistor under dark and UV illumination. Curr. Appl. Phys..

[cit282] Gunaratne K. D., Berkdemir C., Harmon C. L., Castelman Jr A. W. (2012). Investigating the relative stabilities and electronic properties of small zinc oxide clusters. J. Phys. Chem. A.

[cit283] Purica M., Budianu E., Rusu E. (2001). ZnO thin films on semiconductors substrate for large area photo-detector applications. Thin Solid Films.

[cit284] Aoki T., Hatanaka Y., Look D. C. (2000). ZnO diode fabricated by excimer-laser doping. Appl. Phys. Lett..

[cit285] Gorla C. R. (1999). *et al.*, Structural, optical, and surface acoustic wave properties of epitaxial ZnO films grown on (0112) sapphire by metalorganic chemical vapor deposition. J. Appl. Phys..

[cit286] Jo S. H., Lao J. Y., Ren Z. F., Farrer R. A., Baldacchini T., Fourkas J. T. (2003). Field-emission studies on thin films of zinc oxide nanowires. Appl. Phys. Lett..

[cit287] Arnold M. S., Avouris P., Pan Z. W., Wang Z. L. (2003). Field-effect transistors based on single semiconducting oxide nanobelts. J. Phys. Chem..

[cit288] Lin F. C., Takao Y., Shimizu Y., Egashira M. (1995). Hydrogen-sensing mechanism of zinc oxide varistor gas sensor. Sens. Actuators, B.

[cit289] Water W., Chen S. E., Meen T. H., Ji L. W. (2012). ZnO thin film with nanorod arrays applied to fluid sensor. Ultrasonics.

[cit290] Singh J. , Im J., Whitten J. E., Soares J. W., Meehan A. M. and Steeves D. M., Adsorption of mercaptosilanes on nanocrystalline and single crystal zinc oxide surfaces, in Proceedings of the SPIE 7030, Nanophotonic Materials V, 70300T, San Diego, CA, USA, 2008

[cit291] Yan H., He R., Johnson J., Law M., Saykally R. J., Yang P. (2003). Dendritic nanowire ultraviolet laser array. J. Am. Chem. Soc..

[cit292] Senoussaoui N., Krause M., Müller J., Bunte E., Brammer T., Stiebig H. (2004). Thin-film solar cells with periodic grating coupler. Thin Solid Films.

[cit293] Lima S. A. M., Sigoli F. A., Jafelicci Jr M., Davolos M. R. (2001). Luminescent properties and lattice correlation defects on zinc oxide. Int. J. Inorg. Mater..

[cit294] Mikrajuddin, Iskandar F., Okuyama K. (2001). Stable photoluminescence of zinc oxide quantum dots in silica nanoparticles matrix prepared by the combined sol-gel and spray drying method. J. Appl. Phys..

[cit295] Kim M. S., Nam G., Kim S., Kim D. Y., Lee D. Y., Kim J. S., Kim S. O., Kim J. S., Son J. S., Leem J. Y. (2012). Photoluminescence studies of ZnO thin films on R-plane sapphire substrates grown by sol-gel method. J. Lumin..

[cit296] Soares J. W., Whitten J. E., Oblas D. W., Steeves D. M. (2008). Novel photoluminescence properties of surface-modified nanocrystalline zinc oxide: Toward a reactive scaffold. Langmuir.

[cit297] Wang M., Wang X. (2008). Electrodeposition zinc-oxide inverse opal and its application in hybrid photovoltaics. Sol. Energy Mater. Sol. Cells.

[cit298] Wastermark K., Rensmo H., Lees A. C., Vos J. G., Siegbahn H. (2002). Electron spectroscopic studies of bis-(2,2′-bipyridine) -(4,4′-dicarboxy-2,2′-bipyridine)-ruthenium (II) and bis-(2,2′-bipyridine)-(4,4′-dicarboxy-2,2′-bipyridine)-osmium (II) absorbed on nanostructured TiO2 and ZnO. Surf. J. Phys. Chem..

[cit299] Wöll C. (2007). The chemistry and physics of zinc oxide surfaces. Prog. Surf. Sci..

[cit300] Espitia P. J. P., Soares N. F. F., Coimbra J. S. R., de Andrade N. J., Cruz R. S., Medeiros E. A. A. (2012). Zinc oxide nanoparticles: Synthesis, antimicrobial activity and food packaging applications. Food Bioprocess Technol..

[cit301] Moezzi A., McDonagh A. M., Cortie M. B. (2012). Zinc oxide particles: Synthesis, properties and applications. Chem. Eng. J..

[cit302] Klingshirn C. (2007). ZnO: From basics towards applications. Phys. Status Solidi B.

[cit303] Arnold M. S., Avouris P., Pan Z. W., Wang Z. L. (2003). Field-effect transistors based on single semiconducting oxide nanobelts. J. Phys. Chem. B.

[cit304] Costenaro D., Carniato F., Gatti G., Marchese L., Bisio C. (2013). Preparation of luminescent ZnO nanoparticles modified with aminopropyltriethoxy silane for optoelectronic applications. New J. Chem..

[cit305] Liu B. (2012). *et al.*, ZnO-nanoparticle-assembled cloth for flexible photodetectors and recyclable photocatalysts. J. Mater. Chem..

[cit306] Bagabas A., Alshammari A., Aboud M. F. A., Kosslick H. (2013). Room-temperature synthesis of zinc oxide nanoparticles in different media and their application in cyanide photodegradation. Nanoscale Res. Lett..

[cit307] Hong R. Y., Li J. H., Chen L. L., Liu D. Q., Li H. Z., Zheng Y., Ding J. (2009). Synthesis, surface modification and photocatalytic property of ZnO nanoparticles. Powder Technol..

[cit308] Zhang Y., Nayak T., Hong H., Cai W. (2013). Biomedical Applications of Zinc Oxide Nanomaterials. Curr. Mol. Med..

[cit309] Xiong H. M. (2013). ZnO nanoparticles applied to bioimaging and drug delivery. Adv. Mater..

[cit310] Matsuyama K., Ihsan N., Irie K., Mishima K., Okuyama T., Mutod H. (2013). Bioimaging application of highly luminescent silica-coated ZnO nanoparticle quantum dots with biotin. J. Colloid Interface Sci..

[cit311] Rasmussen J. W., Martinez E., Louka P., Wingett D. G. (2010). Zinc oxide nanoparticles for selective destruction of tumor cells and potential for drug delivery applications. Expert Opin. Drug Delivery.

[cit312] Al-Kahlout A. (2012). ZnO nanoparticles and porous coatings for dye-sensitized solar cell application: photoelectrochemical characterization. Thin Solid Films.

[cit313] Smijs T. G., Pavel S. (2011). Titanium dioxide and zinc oxide nanoparticles in sunscreens: focus on their safety and effectiveness. Nanotechnol. Sci. Appl..

[cit314] Jiang J., Pi J., Cai J. (2018). The Advancing of Zinc Oxide Nanoparticles for Biomedical Applications. Bioinorg. Chem. Appl..

[cit315] Sun H., Tian H., Yang Y., Xie D., Zhang Y.-C., Liu X., Ma S., Zhao H.-M., Ren T.-L. (2013). A novel flexible nanogenerator made of ZnO nanoparticles and multiwall carbon nanotube. Nanoscale.

